# Synthesis, Photophysical and TD-DFT Evaluation of Triphenylphosphonium-Labeled Ru(II) and Ir(III) Luminophores

**DOI:** 10.1021/acs.inorgchem.5c04913

**Published:** 2026-01-08

**Authors:** Alexandra R. Ibbott, Steffan Walker-Griffiths, Peter N. Horton, Joseph M. Beames, Catherine L. Andrews, Simon J. Coles, Simon J. A. Pope

**Affiliations:** † School of Chemistry, Main Building, 2112 Cardiff University, Cardiff CF10 3AT, Cymru/Wales, U.K.; ‡ School of Chemistry, 1724 The University of Birmingham, Edgbaston, Birmingham B15 2TT, U.K.; § UK National Crystallographic Service, Chemistry, Faculty of Natural and Environmental Sciences, 7423 University of Southampton, Highfield, Southampton SO17 1BJ, England, U.K.

## Abstract

The chemistry of 5-acetamido derivatized 1,10-phenanthroline was developed to yield a series of pro-ligands (**L** ^**1–4**^) and related triphenylphosphonium (TPP^+^) appended cationic variants (**L** ^**5–8**^). The resulting heteroleptic complexes [Ru­(bipy)_2_(**L** ^**1–8**^)]^*n*+^ (where bipy = 2,2′-bipyridine) and cyclometalated species [Ir­(tmq)_2_(**L** ^**1–8**^)]^*n*+^ (where tmq = 2,6,7-trimethyl-3-phenylquinoxaline) were isolated and fully characterized using a range of analytical and spectroscopic methods, including electrochemistry and time-resolved photophysics. Multinuclear NMR spectroscopies were used to characterize the compounds, including ^31^P NMR showing δ_P_ 21.3–24.4 ppm for the phosphonium species. Two X-ray crystal structures were successfully obtained on TPP^+^ functionalized Ru­(II) and Ir­(III) species: key features include the distorted octahedral coordination spheres, and the defined spatial relationships between the complex core and the TPP^+^ unit. All Ru­(II) and Ir­(III) complexes were phosphorescent in the red region with ^3^MLCT or ^3^MLCT/^3^LLCT character, respectively. Comparison across the series suggest the presence of the TPP^+^ unit induced moderate quenching of the complex phosphorescence. A comparison with quaternary ammonium analogues suggests this may be due to differences in ion pairing and solvation phenomena in the TPP^+^ complexes.

## Graphical abstract

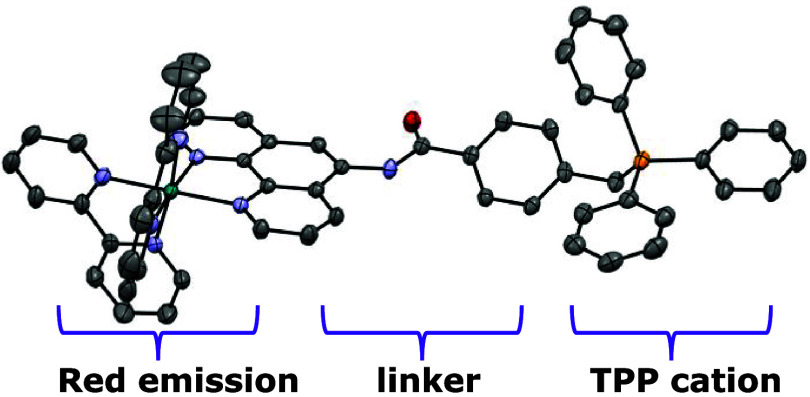

## Introduction

The triphenylphosphonium (TPP^+^) moiety is a well-known functional group that has been explored in a number of guises within various subdisciplines of chemistry research. Phosphonium salts have many industrial applications^1^ and are a key functional group in synthetic chemistry transformations.^2^ While TPP^+^ salts made from various inorganic acids have been known for many decades,^3^ the efficient and convenient synthesis of aryltriphenylphosphonium derived salts remains an active area of research.^4^ The cationic TPP^+^ moiety is highly lipophilic and has been extensively studied in pharmaceutical applications,^5^ including for drug design and drug delivery.^6^ Some of the very earliest studies established TPP^+^ cations as tools for studying the biology of mitochondria,^7^ and significant advances have developed since,^8^ including in therapeutic^9^ and diagnostic applications.^10^ Furthermore, radiolabeled phosphonium salts have been proposed as mitochondrial voltage sensors using positron emission tomography (PET) myocardial imaging.^11^

The combination of TPP^+^ cations with metal ion coordination complexes is attractive because of their combined utility in a biological context. For example, ^64^Cu-labeled radioimaging agents have been developed that incorporate a TPP^+^ functionality^12^ and have demonstrated high tumor-selectivity in mice studies.^13^ ^99m^Tc-labeled organometallic agents have also been functionalized with a TPP^+^ unit and proposed as potential radioimaging probes.^14^ Additional reports describe targeted magnetic resonance imaging (MRI) contrast agents based on Gd­(III) complexes^15^ of DOTA (1,4,7,10-tetraazacyclododecane-*N,N,N,N*-tetraacetic acid) ligands that integrate the TPP^+^ moiety into the peripheral ligand architecture. The clinical MRI agent [Gd­(DOTA)]^−^ is an extracellular agent; functionalization with TPP^+^ units repositions such species for intracellular localization,^16^ including related tetraazamacrocyclic Gd­(III) complexes for targeting tumors.^17^ A high molecular weight Gd­(III) macrocyclic complex adorned with a TPP^+^ moiety has also been investigated as an in vivo *T*_2_ MRI contrast agent for studying stem cell transplants.^18^ The broad utility of DOTA-like chelates^19^ is such that the radiosynthesis of related ^67^Ga-labeled phosphonium-tagged complexes has also been reported.^20^

Nonluminescent Pt­(II) complexes have been synthesized with N-heterocyclic carbene (NHC) ligands adorned with pendant TPP^+^ units and explored in biological studies;^21^ related phosphonium tethered NHC ligands have also been reported within Au­(I) complexes.^22^ A Pt­(IV) pro-drug species has been shown to target and accumulate in mitochondria, driven by the peripheral TPP^+^ groups.^23^ Potent cytotoxic Cu­(II)^24^ and V­(IV) TPP^+^ complexes^25^ have both been developed and, again, shown to target mitochondria.

Given the powerful utility of TPP^+^ cations it is surprising that luminescent bioimaging agents that incorporate them have not been evaluated in more detail. Very recent examples include mitochondrial-targeting fluorescent systems driven by aggregation induced emission character,^26^ and a fluorescent BODIPY-TPP^+^ conjugate proposed for mitochondrial targeted photodynamic therapy.^27^

The development of luminescent coordination complexes with TPP^+^ units is similarly underreported, which, given the myriad benefits presented by metal-based luminophores in a bioimaging context, is surprising.^28^ A luminescent N∧N∧C cyclometalated Pt­(II) complex has been reported which incorporates a tethered TPP^+^ unit and exhibited nucleolus-targeted behavior imaged via two-photon confocal fluorescence microscopy.^29^ Of direct relevance to the current study (Figure 1) are a polypyridyl Ru­(II) complex that has been positioned as a two-photon photodynamic therapeutic agent,^30^ and heteroleptic [Ir­(C∧N)_2_(L∧L)]^n+^ cyclometalated complexes that have shown viable cell imaging capability and mitochondrial targeting attributes.^31^

**Figure 1:**
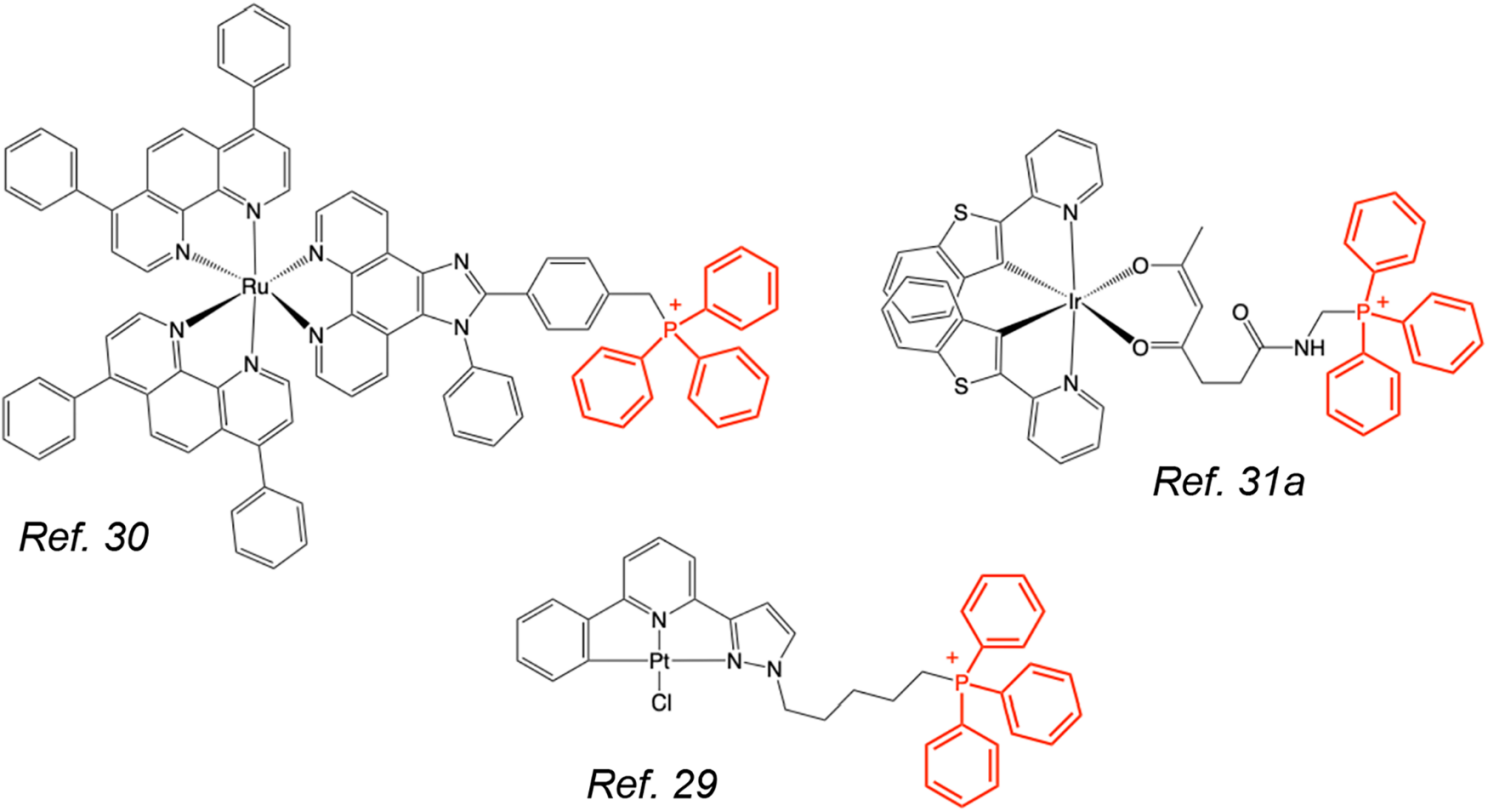
Molecular structures of three examples of photoluminescent metal complex covalently functionalized with a triphenylphosphonium cation (highlighted in red).

The aim of this work was to explore a series of luminescent Ru­(II) and Ir­(III) complexes that integrate a cationic TPP^+^ moiety linked to the parent complex in different ways (Figure 2). Given the potential of such species in a luminescence bioimaging context, we show that there is evidence for solvent dependent quenching of the triplet emitting character of the complexes, which is dependent upon the type of linker unit within the ligand architecture and solvent conditions.

**Figure 2:**
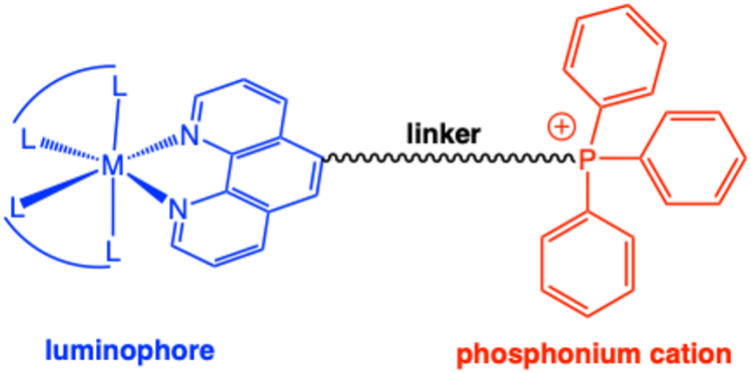
General schematic for the design of the luminescent complexes presented in this study (M = Ru^2+^, Ir^3+^).

## Results and Discussion

### Synthesis of the Ligands

The general design of the new complexes is represented in Figure 2 and utilizes a 1,10-phenanthroline chelator that is functionalized with a cationic TPP^+^ moiety via a linking tether. The isolation of the final TPP^+^ functionalized ligands (**L** ^**5**^–**L** ^**8**^) was achieved by first developing intermediate species with a terminating chloromethyl group; these intermediates were also utilized as pro-ligands (**L** ^**1**^–**L** ^**4**^) in their own right (Scheme 1). The ligands encompass different types of bridging group (e.g., benzamide, propanamide, acetamide, for **L** ^**1**^ **–L** ^**3**^, respectively, and a *N*-piperazinyl-benzamide for **L** ^**4**^) which generally offer restricted flexibility and should therefore modify the spatial relationship between the terminal –PPh_3_^+^ group and the coordination sphere. **L** ^**1**^–**L** ^**3**^ were easily isolated in a single step from reaction of 5-amino-1,10-phenanthroline and the relevant acid chloride; **L** ^**1**^–**L** ^**3**^ ^32^ were then transformed to the corresponding phosphonium species (**L** ^**5**^–**L** ^**7**^) by reacting with PPh_3_ in the presence of KI.

**Scheme 1:**
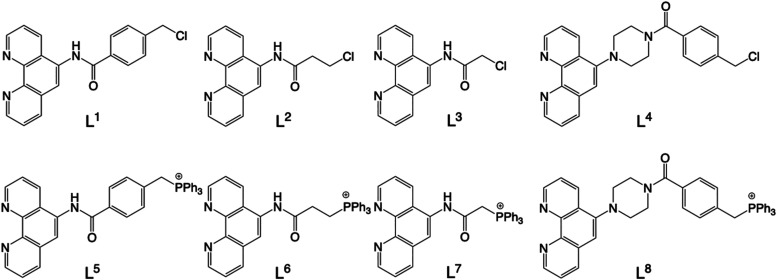
Structures of the Isolated Ligands, **L** ^**1**^ **–L** ^**8**^

The synthetic approach to the **L** ^**4**^/**L** ^**8**^ pair of ligands was different (Scheme 2) utilizing 5,6-epoxy-5,6-dihydro-1,10-phenanthroline as a starting material.^33^ First, reaction with excess piperazine initially formed a 6-amino substituted, 5,6-dihydro-1,10-phenanthrolin-5-ol species^34^ which was treated with NaH in THF to induce rearomatization.^35^ Further reaction of the piperazin-1-yl adduct (Scheme 2)^36^ with 4-(chloromethyl)­benzoyl chloride yielded **L** ^**4**^ which was then converted directly into **L** ^**8**^ in an analogous manner to that described above for **L** ^**5**^–**L** ^**7**^.

**Scheme 2:**
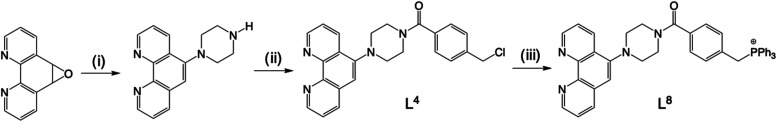
Synthetic Route to **L** ^**8**^ via Its Precursor **L** ^**4**^ ^a^

a Reagents and conditions: (i) piperazine, heat; then NaH in THF; (ii) 4-(chloromethyl)­benzoyl chloride, DCM; (iii) PPh_3_, KI, MeCN.

The ligands were fully characterized using a range of spectroscopic techniques and analyses. For **L** ^**1**^, **L** ^**3**^ and **L** ^**4** 1^H NMR spectra gave a characteristic singlet resonance for the chloromethyl unit in the range of 4.0–4.8 ppm (for **L** ^**2**^ the −C*H*_2_Cl environment appears as a triplet). In each case the unsymmetrical nature of the 1,10-phenanthroline unit generally induced distinguishable aromatic resonances for each aromatic proton environment; the furthest downfield signals (typically 9.0–9.3 ppm) were associated with the protons in the 2,9-positions of the phenanthroline ring. When observable, relevant N*H* resonances appeared ca. 9 ppm. The ^13^C­{^1^H} NMR spectra revealed a resonance for the chloromethyl fragment around 40–45 ppm, as well as numerous aromatic signals consistent with each species. Upon conversion to the cationic phosphonium species, the resultant −C*H*_2_PPh_3_^+^ resonance was shifted downfield (∼ 5.1 ppm), and in the cases of **L** ^**5**^ and **L** ^**8**^ appeared as a nicely resolved doublet (^2^ *J*_HP_). ^31^P­{^1^H} NMR spectroscopy was used to confirm a single phosphonium environment in **L** ^**5**^-**L** ^**8**^ noted at 21.9–24.7 ppm; the spectra indicate a downfield shift from, and an absence of, free PPh_3_ (−7 ppm). All relevant spectra are presented in the SI (Figures S1–S20).

For **L** ^**1**^–**L** ^**3**^ and **L** ^**5**^–**L** ^**7**^ the formation of the amido group was also indicated by the IR spectrum with an observable stretch ca. 1690 cm^–1^; **L** ^**1**^–**L** ^**4**^ also report a medium strength C–Cl vibrational stretch ca. 700–750 cm^–1^ which was absent in the corresponding phosphonium derivatives. High resolution mass spectra were obtained for each ligand showing *m*/*z* values consistent with [M + H]^+^ in the cases of **L** ^**1**^–**L** ^**4**^ and [M]^+^ for **L** ^**5**^–**L** ^**8**^.

### Synthesis of the Complexes

A total of 16 complexes were obtained for this study, with a primary focus upon long-wavelength phosphorescent characteristics that may be applicable to future bioimaging studies. Thus, ruthenium complexes of the form [Ru­(bipy)_2_(**L** ^**1–8**^)]^*n*+^ were synthesized according to previous procedures whereby reaction of well-known [RuCl_2_(bipy)_2_] with the relevant ligand (and excess NaPF_6_ in refluxing EtOH) resulted in the formation of the quintessentially orange-red colored solution typical of polypyridine Ru­(II) species. The analogous cyclometalated iridium complexes, [Ir­(tmq)_2_(**L** ^**1–8**^)]^*n*+^, (where tmq = 2,6,7-trimethyl-3-phenylquinoxaline) were obtained in accordance with previous studies^37^ where [Ir­(tmq)_2_(MeCN)_2_]^+^ was treated with 1 equiv of ligand to yield the heteroleptic targets (Scheme 3). The trimethylated version of 2-phenylquinoxaline provides two advantages as a cyclometalating ligand: it imparts excellent solubility upon the dimer precursor, and yields heteroleptic complexes with efficient red phosphorescence.

**Scheme 3:**
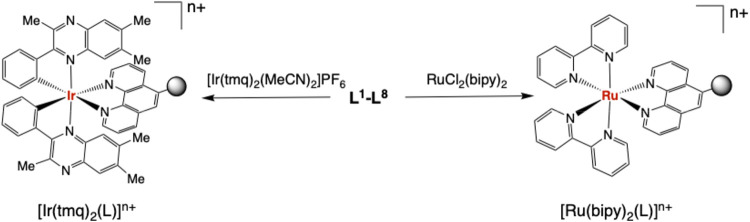
Final Synthetic Step to the Different Ru­(II) and Ir­(III) Complexes

The formation of [Ru­(bipy)_2_(**L** ^**1–8**^)]­(PF_6_)*_n_* was initially established using ^1^H NMR spectroscopy. In the complexes of **L** ^**1**^-**L** ^**4**^ the retention of the chloromethyl functionality was evidenced by the resonance around 4.5–5.0 ppm. As the unsymmetrical nature of the 1,10-phenanthroline ligand renders subtle inequivalence across the two bipyridine chelates, the aromatic region typically featured numerous overlapping signals, although in most cases these were quite well resolved (see SI for details). For the complexes featuring **L** ^**1**^-**L** ^**3**^, the N*H* resonance of the amide functionality was noted further downfield ca. 10.0–10.5 ppm. When converted to the corresponding phosphonium complexes, [Ru­(bipy)_2_(**L** ^**5–8**^)]­(PF_6_)_3_, the additional phenyl protons were mainly noted at 7.5–8.0 ppm and thus superimposed upon other ligand resonances. For the piperazine bridged species, [Ru­(bipy)_2_(**L** ^**4**^)]­(PF_6_)_2_ and [Ru­(bipy)_2_(**L** ^**8**^)]­(PF_6_)_3_ (Scheme 4) the additional aliphatic signals were noted between 3.0–4.5 ppm and were typically broadened in appearance (Figures S27 and S38). The −C*H*_2_–PPh_3_^+^ resonance was observed as a well-resolved doublet (^2^ *J*_HP_) for the complexes based on **L** ^**5**^ and **L** ^**8**^. ^31^P­{^1^H} NMR spectra for the Ru­(II) complexes gave two main features: first, a singlet phosphonium resonance at 22.7–24.4 ppm (and thus closely comparable to the free ligands), and second, a characteristic septet (^1^ *J*_PF_) ca. −144.6 ppm due to the hexafluorophosphate counterions. All relevant spectra are presented in the SI (Figures S21–S40).

**Scheme 4:**
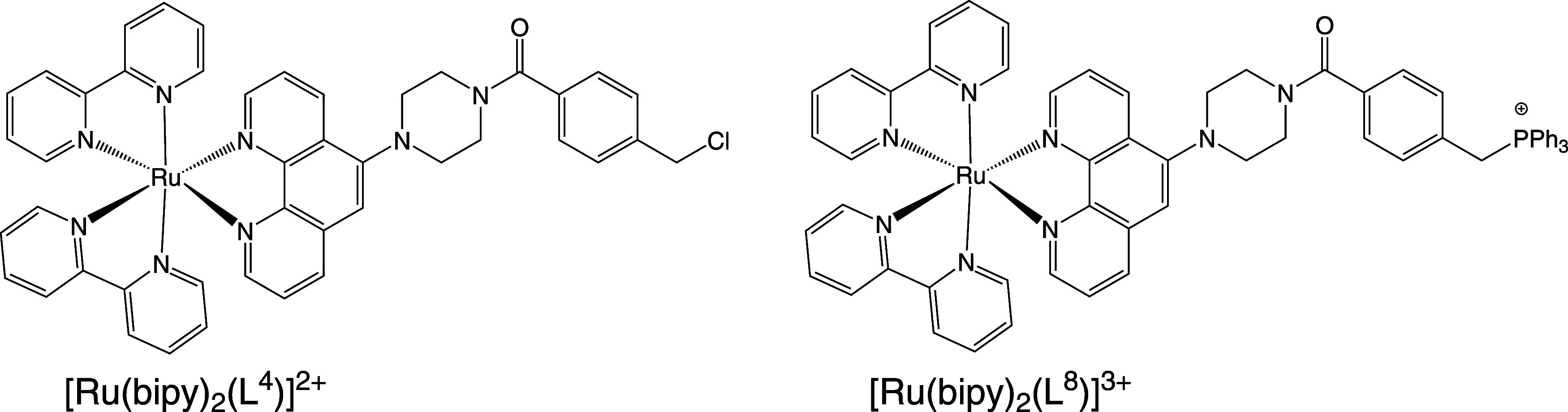
Comparison of the Structures of the Ru­(II) Complexes of **L^4^** and **L^8^**

Within the series of iridium complexes, the cyclometalation of the tmq ligands affords helpful spectroscopic handles with respect to the ^1^H NMR spectra. The Ir­(III) complexes typically showed a set of aliphatic resonances that were attributable to the six different methyl environments of the inequivalent tmq ligands (again, induced by unsymmetrical **L** ^**1**^–**L** ^**8**^). Across the eight Ir­(III) complexes the methyl resonances appeared (usually as three groupings of two singlets) in the range 1.5–3.5 ppm showing first, the different levels of shielding that are induced by the interligand spatial relationships within the complexes, and second, the subtle inequivalence of the tmq ligands. The ^31^P­{^1^H} NMR data for [Ir­(tmq)_2_(**L** ^**5–8**^)]­(PF_6_)_2_ again confirmed the phosphonium resonance at 21.3–24.3 ppm together with an upfield septet for PF_6_^–^. All relevant spectra are presented in the SI (Figures S41–S64).

### X-ray Crystal Structures of **L** ^**5**^ and [Ru­(bipy)_2_(**L** ^**5**^)]­(PF_6_)_3_

Two X-ray crystal structures were obtained from single yellow crystals of **L** ^**5**^ (grown from slow evaporation of CD_3_OD); these were either blade or rod-shaped in appearance. The structures were solved in the *P*2_1_/*c* and *P*2_1_/*n* space groups, respectively, and confirm the structure of the cationic fragment of the ligand salt; they differ only with respect to the identity of the counteranion: in one case a linear (174.528(5)°) tri-iodide ion (which may result from the trace impurity of iodine in KI) provides the charge balance, while in the other structure there is disorder of iodide and chloride ions at noninteger values (the latter structure is shown in Figure 3). The data collection parameters for both crystal structures of **L** ^**5**^ are shown in Table S1, SI. In both determined structures, the TPP^+^ fragment adopts an approximately tetrahedral geometry at phosphorus with bond angles in the range of 106.48(9)–112.10(9)°.

**Figure 3:**
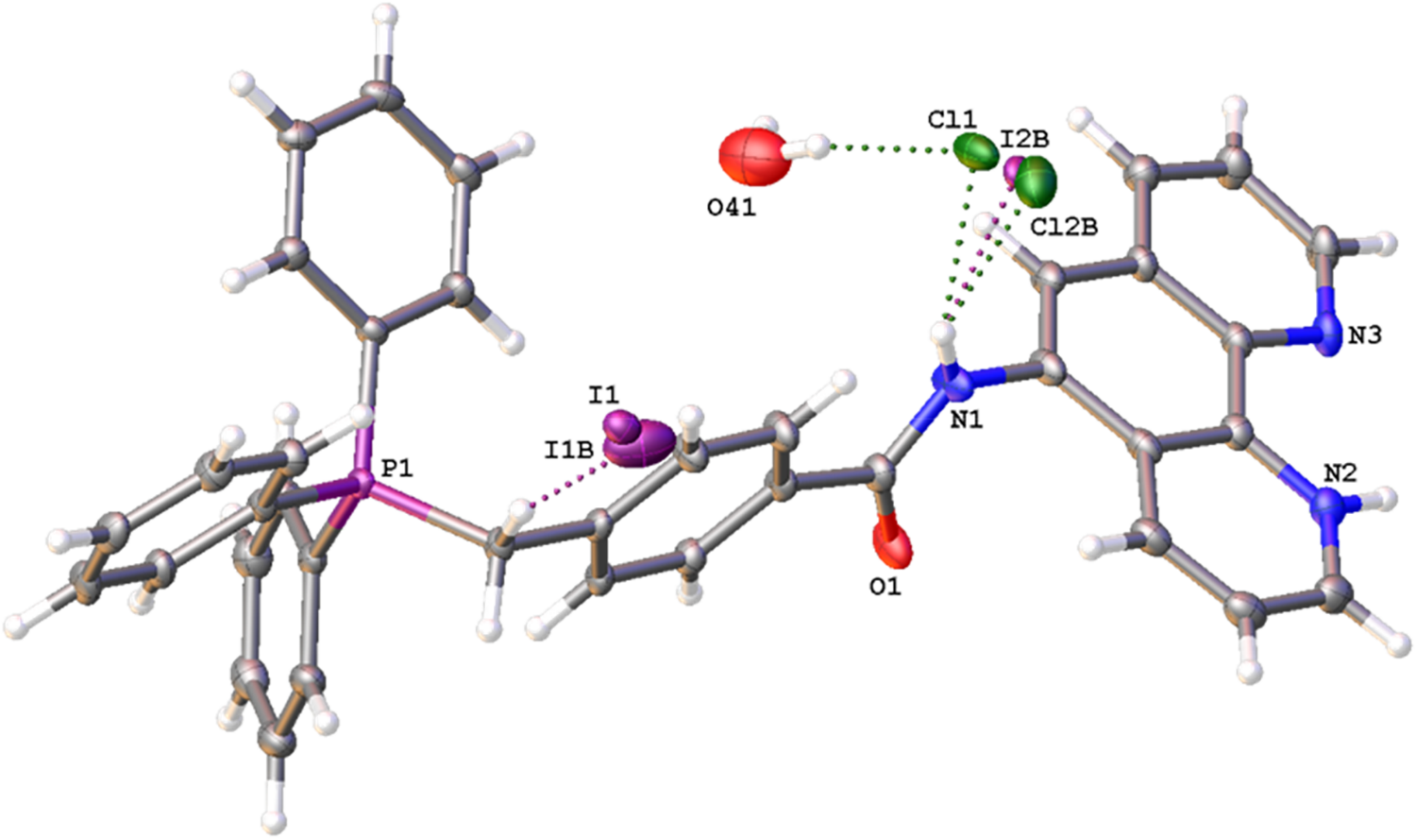
X-ray crystal structure of **L** ^**5**^. Ellipsoids drawn at 50%. There is disorder of iodide and chloride ions at noninteger values.

For [Ru­(bipy)_2_(**L** ^**5**^)]­(PF_6_)_3_, single orange blade-shaped crystals were obtained from a concentrated methanol solution of the complex. The structure (Figure 4) was solved in the *P*-1 space group; there is a single formula unit in the asymmetric unit, which is represented by the reported sum formula (Z is 2 and Z′ is 1). The data collection parameters are tabulated in the SI with selected bond lengths and angles shown in Table 1. The obtained structure was consistent with the proposed formulation and supporting spectroscopic and analytical data.

**Figure 4:**
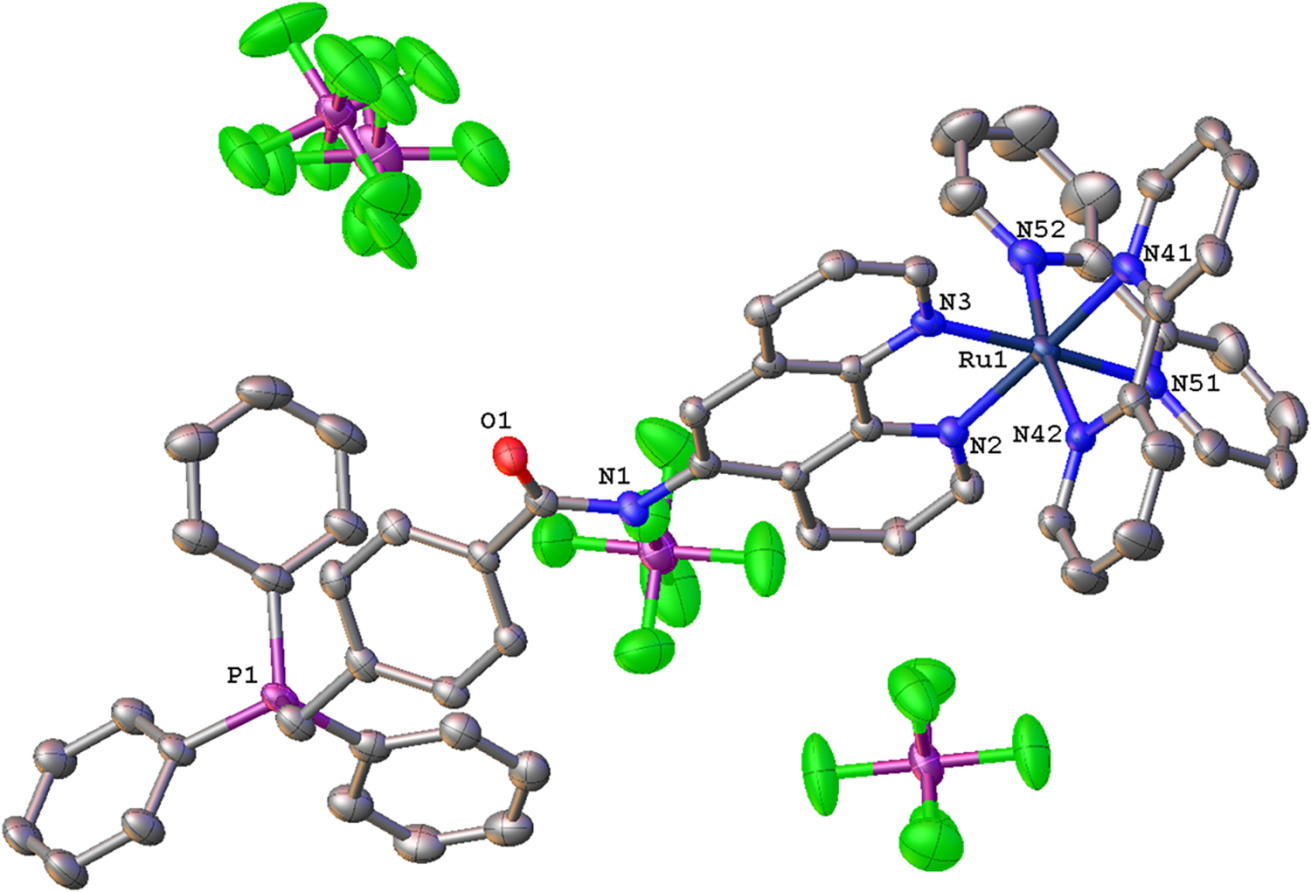
X-ray crystal structure of [Ru­(bipy)_2_(**L** ^**5**^)]­(PF_6_)_3_. Hydrogen atoms have been omitted for clarity. Ellipsoids drawn at 50%.

**Table 1:** Selected Bond Lengths and Bond Angles for [Ru­(bipy)_2_(**L** ^**5**^)]­(PF_6_)_3_

bond lengths (Å)		bond angles (°)			
Ru1–N2	2.063(3)	N2–Ru1–N3	79.17(10)	N42–Ru1–N51	95.44(12)
Ru1–N3	2.077(3)	N2–Ru1–N51	95.53(11)	N42–Ru1–N52	171.86(11)
Ru1–N41	2.063(3)	N41–Ru1–N2	174.43(12)	N51–Ru1–N3	174.34(10)
Ru1–N42	2.052(3)	N41–Ru1–N3	95.87(11)	N52–Ru1–N2	88.42(11)
Ru1–N51	2.063(3)	N41–Ru1–N51	89.51(11)	N52–Ru1–N3	99.14(12)
Ru1–N52	2.054(3)	N42–Ru1–N2	97.74(11)	N52–Ru1–N41	94.93(12)
		N42–Ru1–N3	87.29(11)	N52–Ru1–N51	78.60(13)
		N42–Ru1–N41	79.36(11)		

The bond angles that define the coordination sphere are typical of a distorted octahedral geometry and closely comparable with related polypyridine complexes of Ru­(II).^38^ The Ru–N bond lengths lie within a narrow range 2.042(3)–2.077(3) Å and are also consistent with previous relevant studies, including polycationic Ru­(II) variants.^39^ The ∠C–P–C angles that describe the TPP^+^ fragment lie in the range 106.97(16)–112.92(17)°, and are closely comparable with those measured for the corresponding free ligand. The amide group of **L** ^**5**^ has an angle of 9.2(3)° with respect to the plane of the 1,10-phenanthroline unit; the attached benzylic moiety is then twisted out of the plane defined by those two groups (31.61(14)° for benzylic to amide; 37.91(9)° for benzylic to phenanthroline). The three counterions are dispersed around the complex, with one PF_6_^–^ showing a N–H···F hydrogen bonding interaction at ca. 2.14 Å.

### X-ray Crystal Structure of [Ir­(tmq)_2_(**L** ^**6**^)]­(PF_6_)_2_

Single red blade-shaped crystals were grown from the vapor diffusion of diisopropyl ether into a concentrated acetonitrile solution of the complex. The structure was solved in the *P*1̅ space group, with two formula units in the asymmetric unit (Z is 4 and Z′ is 2). The structure obtained is not of the highest quality, but sufficient to show structural connectivity, with both [Ir­(tmq)_2_(**L** ^**6**^)]^2+^ cations disordered, one over the majority of the cation, the second just from the amide side arm of the **L** ^**6**^ ligand, which is used for the example measurements that follow. The collection parameters (Table S1) and selected bond lengths and angles (Table 2) are included for completion. Again, the resultant structure revealed the expected complex formulation (Figure 5).

**Figure 5:**
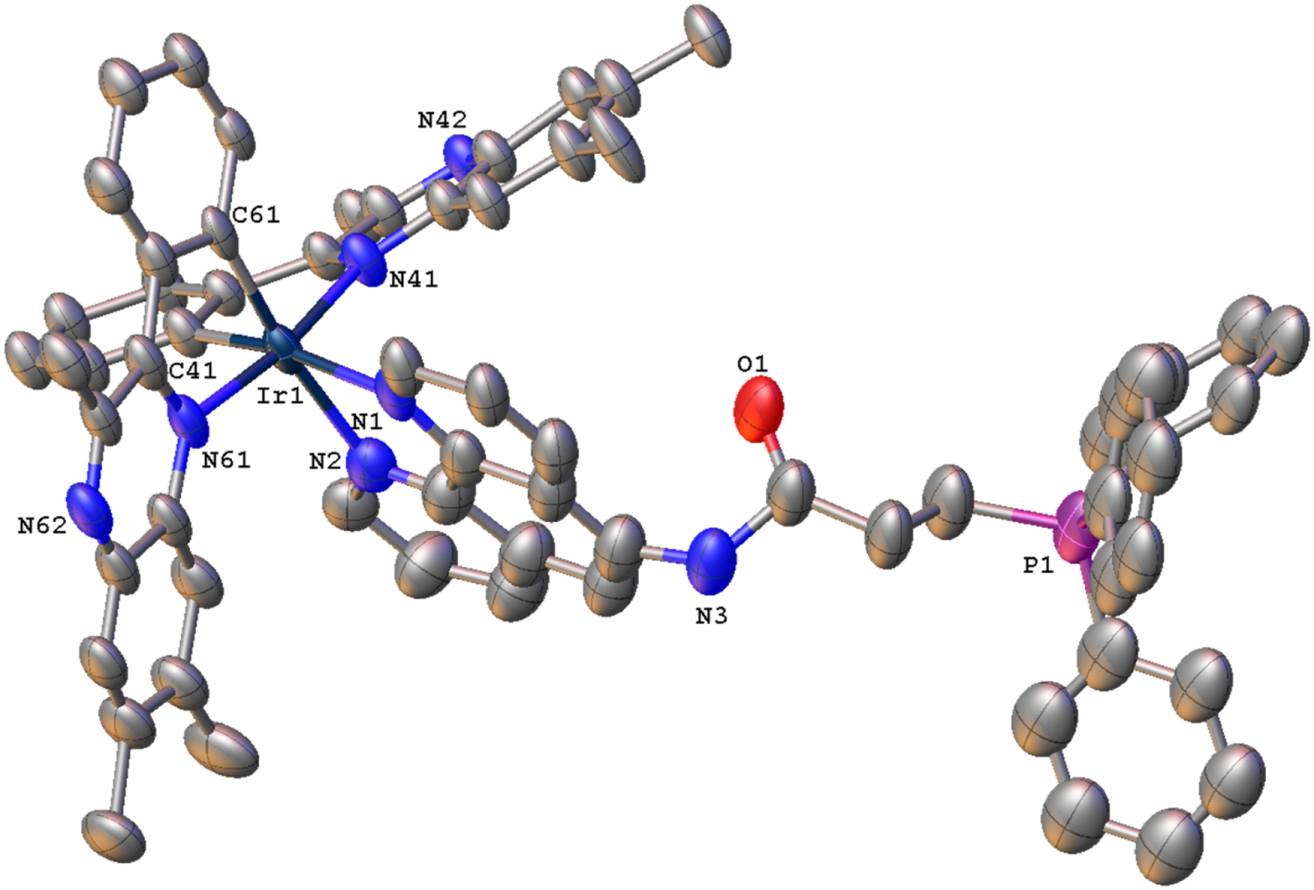
X-ray crystal structure of one of the two cations of [Ir­(tmq)_2_(**L** ^**6**^)]­(PF_6_)_2_. Only the primary disordered component is shown with hydrogen atoms and counterions omitted for clarity. Ellipsoids drawn at 30%.

**Table 2:** Selected Bond Lengths and Bond Angles for [Ir­(tmq)_2_(**L** ^**6**^)]­(PF_6_)_2_

bond lengths (Å)		bond angles (°)			
Ir1–N1	2.147(15)	N1–Ir1–N2	75.4(7)	C41–Ir1–N41	79.3(5)
Ir1–N2	2.24(2)	N41–Ir1–N1	102.4(5)	C41–Ir1–N61	95.5(5)
Ir1–N41	2.091(12)	N41–Ir1–N2	79.8(6)	C41–Ir1–C61	89.5(5)
Ir1–N61	2.060(13)	N61–Ir1–N1	84.0(5)	C61–Ir1–N1	101.0(6)
Ir1–C41	1.990(8)	N61–Ir1–N2	107.4(6)	C61–Ir1–N2	172.8(5)
Ir1–C41	2.040(10)	C41–Ir1–N1	169.0(6)	C61–Ir1–N41	95.1(5)
		C41–Ir1–N2	94.5(6)	C61–Ir1–61	78.2(5)

The coordination sphere of [Ir­(tmq)_2_(**L** ^**6**^)]­(PF_6_)_2_ can be described as a distorted octahedral geometry, with the two tmq ligands imposing a *cis*-C,C and *trans*-N,N arrangement at iridium. The Ir–N and Ir–C bond lengths are typical of closely related [Ir­(tmq)_2_(N^∧^N)]^*n*+^ species, including tricationic complexes.^40^ The packing diagram revealed several intermolecular interactions, which are best described by π–π contacts involving the tmq ligands on adjacent complexes, as well as slightly longer contacts between the phenyl ring of a phosphonium unit and a neighboring tmq ligand. Finally, the details of the ligand conformation are noteworthy: in contrast to [Ru­(bipy)_2_(**L** ^**5**^)]­(PF_6_)_3_, the amide carbonyl is significantly twisted out of the plane defined by the 1,10-phenanthroline unit, which may be due to the intermolecular contacts that support the packing in the crystalline form of [Ir­(tmq)_2_(**L** ^**6**^)]­(PF_6_)_2_.

### Electronic and Redox Properties of the Complexes

The redox properties of the complexes were investigated in deoxygenated MeCN using cyclic voltammetry. The Ru­(II) complexes typically showed (Figure S69, SI) one oxidation wave around +0.85 V assigned to the Ru­(II)/(III) couple,^41^ and closely comparable to the values for [Ru­(bipy)_3_]^2+^ and [Ru­(bipy)_2_(phen)]^2+^,^42^ which was generally reversible (especially for complexes of **L** ^**1**^–**L** ^**4**^) or quasi-reversible. This oxidation process was relatively insensitive to the addition of a cationic charge (via TPP^+^) in [Ru­(bipy)_2_(**L** ^**5–8**^)]­(PF_6_)_3_. The Ru­(II) complexes also gave two or three identifiable one electron processes in the cathodic region (especially between −1.5 to −2.25 V) and these are attributed to sequential ligand-based reduction processes, as previously noted for Ru­(II)-polypyridines.^43^ It is important to note that phosphonium salts are generally regarded as electrochemically inert across a wide redox window (e.g., application in ionic liquid electrolytes).^44^

The redox properties of the Ir­(III) complexes yielded a similar pattern. For reference, [Ir­(ppy)_2_(bipy)]^+^ (where ppy = 2-phenylpyridine) shows two quasi-reversible waves: one oxidative (Ir^3+/4+^) and one bipy-based reduction (note that reduction of anionic ppy is regarded as unfavorable).^45^ Here, an irreversible oxidation around +1.0 V was noted and likely relates to the Ir-centered process. Several ligand-based processes were also noted in the cathodic window, some of which were clearly reversible.^46^ Since quinoxaline is a better π-acceptor than pyridine (i.e., the difference between tmq and ppy), it is feasible that both phen and quinoxaline-based reductions are present for [Ir­(tmq)_2_(**L** ^**1–8**^)]^*n*+^. Overall, comparison of the data within each series shows pendant positive charges added to the chelating ligands causes only minor perturbations of the redox potentials, correlating with our previous observations on polycationic Ru­(II) and Ir­(III) species.^39,40^

The UV–vis. absorption spectra of the free ligands (**L** ^**1**^ **–L** ^**8**^) demonstrate absorption properties dominated by UV wavelength transitions. The ligands all show two intense absorptions <300 nm which can be attributed to spin allowed (S_0_ → S*_n_*) π → π* transitions that are localized on the phenanthroline moiety. For the phosphonium derivatives the relative intensity of a band ca. 240 nm was increased and thus assigned to π → π* transitions localized on the phenyl substituents.

[Ru­(bipy)_2_(**L** ^**1–8**^)]^*n*+^ gave spectra (Figure 6) where the UV region was dominated by the summative effect of overlapping ligand-based π → π* transitions, with a particularly strong band at 280 nm which is known to be associated with bipy and phen-based π → π* transitions. Additional absorptions in the visible region, most notably the spin-allowed ^1^MLCT ca. 450 nm (ε_MLCT_ > 1.5 × 10^4^ M^–1^ cm^–1^) are present; the relative intensities of the vibronic progressions within the MLCT band vary across the series.^47^ In particular, for the closely related complexes of **L** ^**1**^ and **L** ^**5**^, the most intense contribution to the MLCT band lies at ca. 425 nm suggesting the chemical nature of the amido functionality is influential. The MLCT band is relatively unperturbed by the presence of the TPP^+^ moiety; overall the spectra broadly resemble that of the benchmark species [Ru­(bipy)_3_]^2+^.^48^

**Figure 6:**
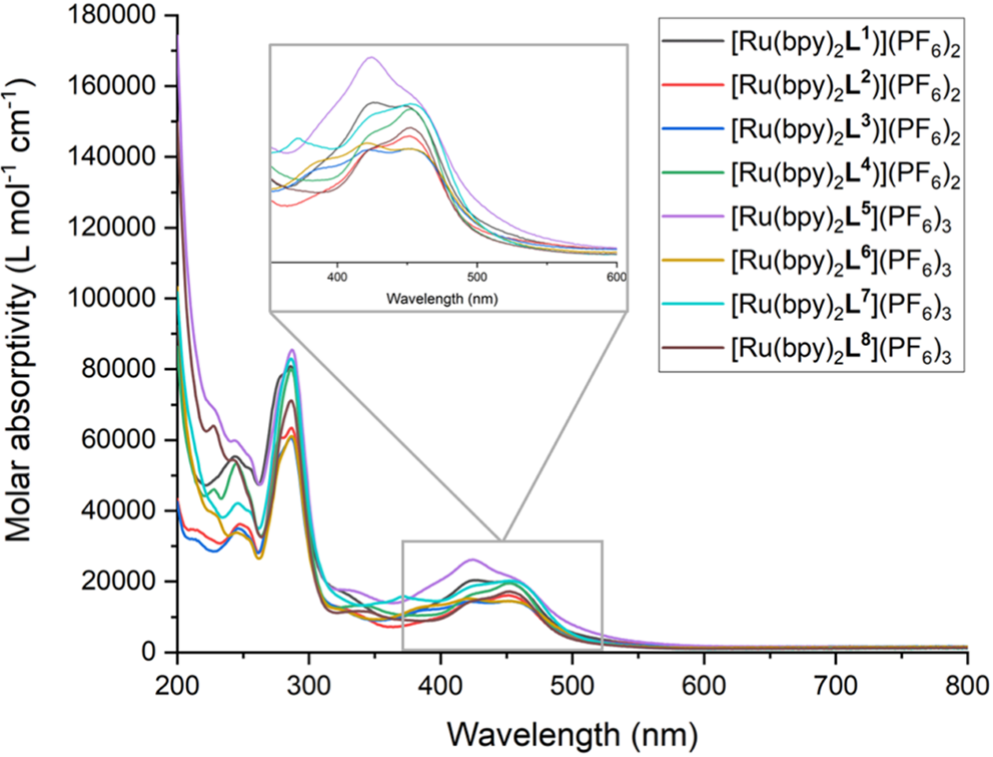
UV–vis. absorption spectra for the series of [Ru­(bipy)_2_(**L**)]­(PF_6_)_n_ complexes (293 K, aerated MeCN, 3.33 × 10^–6^ M). Inset: expansion of ^1^MLCT band envelope. the corresponding emission spectra (293 K, aerated MeCN, λ_ex_ = 450 nm).

For the Ir­(III) complexes, the experimental UV–vis absorption spectra showed a similar trend (Figure 7). First, intense ligand-based bands <300 nm can be assigned to π → π* transitions, as noted above. Absorption bands between 350–500 nm can be attributed to admixtures of charge transfer bands that include both spin allowed LLCT (likely phenyl to quinoxaline in character, and thus distinct from the LLCT observed in [Ir­(ppy)_2_(bipy)]^+^) and MLCT transitions. As noted previously,^49^ the long tail of the MLCT/LLCT band likely comprises a spin forbidden contribution (S_0_ → T_1_; ^3^MLCT/^3^LLCT) at lower intensities (ε < 1 × 10^3^ M^–1^ cm^–1^) that can be facilitated by the very high spin–orbit coupling constant of iridium.^50^ Overall, the appearance of the spectra are closely comparable across the series of Ir­(III) complexes, again showing the minimal influence of the TPP^+^ moiety.

**Figure 7:**
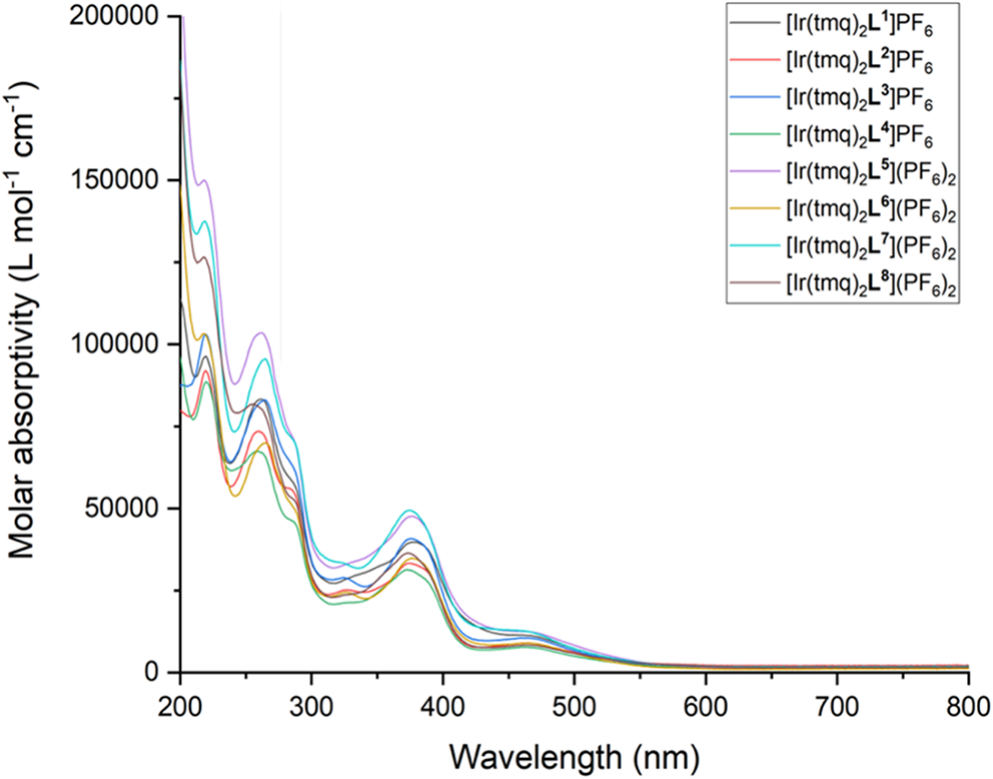
UV–vis absorption spectra for the series of [Ir­(tmq)_2_(**L**)]­(PF_6_)_n_ complexes (293 K, aerated MeCN, 3.33 × 10^–6^ M).

Supporting TD-DFT calculations were undertaken focusing upon the TPP^+^ derivatives. First, for [Ru­(bipy)_2_(**L** ^**5–8**^)]^3+^ the predicted spin allowed transitions involved in the visible absorption bands arise from occupied orbitals with ≥70% Ru­(4d) character (Tables 3, and S3–S5). Complexes of **L** ^**5**^, **L** ^**6**^ and **L** ^**7**^ were all closely comparable; a strong HOMO → LUMO transition dominates the longest wavelength absorption, where the LUMO appears distributed across all chelating ligands. For [Ru­(bipy)_2_(**L** ^**8**^)]^3+^ the change in the architecture of the functionalized phenanthroline ligand appears to alter the location of the HOMO, with HOMO – 1 becoming important in the lowest energy excitation. The LUMO and LUMO + 1 are mainly situated on the bipy ligands and are predicted to be relevant to the lowest energy excitations; the functionality of **L** ^**8**^ raises the energy of LUMO+3 in these calculations which is located on the phen part of the ligand.

**Table 3:** Description of the Calculated MO Contributions, Excited States Descriptions and Their Associated Transitions for [Ru­(bipy)_2_(**L** ^**5**^)]^3+^, Where X Corresponds to the Combined bipy and Phenanthroline Ligands, and Y to the Phenanthroline Substituent Onwards

	moiety contribution to orbital (%)			orbital contribution to excited state	
orbital	Ru(4d) (%)	X (%)	Y (%)	excited state	contributing transitions (>10%)
LUMO + 4	1	86	13	1 (492 nm *f* = 0.0013)	HOMO → LUMO (79.9%)
					HOMO → LUMO + 1 (14.2%)
LUMO + 3	2	88	10	2 (484 nm *f* = 0.0011)	HOMO → LUMO + 1 (75.3%)
LUMO + 2	7	93	0		HOMO → LUMO (18.1%)
LUMO + 1	6	92	2		
LUMO	2	97	1		
HOMO	73	27	0	3 (491 nm *f* = 0.002)	HOMO → LUMO + 2 (95.8%)
HOMO – 1	60	39	1	4 (456 nm *f* = 0.0133)	HOMO – 2 → LUMO (40.3%)
					HOMO – 2 → LUMO + 1 (25.3%)
HOMO – 2	66	34	0	5 (453 nm *f* = 0.0541)	HOMO – 1 → LUMO (68.3%)
					HOMO – 1 → LUMO + 1 (19.6%)
HOMO – 3	7	62	31	6 (451 nm *f* = 0.0099)	HOMO – 2 → LUMO (45.3%)
					HOMO – 1 → LUMO + 2 (35.7%)
HOMO – 4	0	3	97		

The calculated S_0_ → S_1_ values for the Ru­(II) complexes correlate reasonably with the experimental spectra and lie in the range 491–500 nm (Table 4) and are confirmed to be MLCT in nature. As expected, the TD-DFT suggests that the TPP^+^ unit does not host any orbital contributions relevant to the visible region excitations. The calculated Kohn–Sham orbitals for [Ru­(bipy)_2_(**L** ^**5–8**^)]^3+^ are pictorially represented in Figures 8 and S70–72.

**Figure 8:**
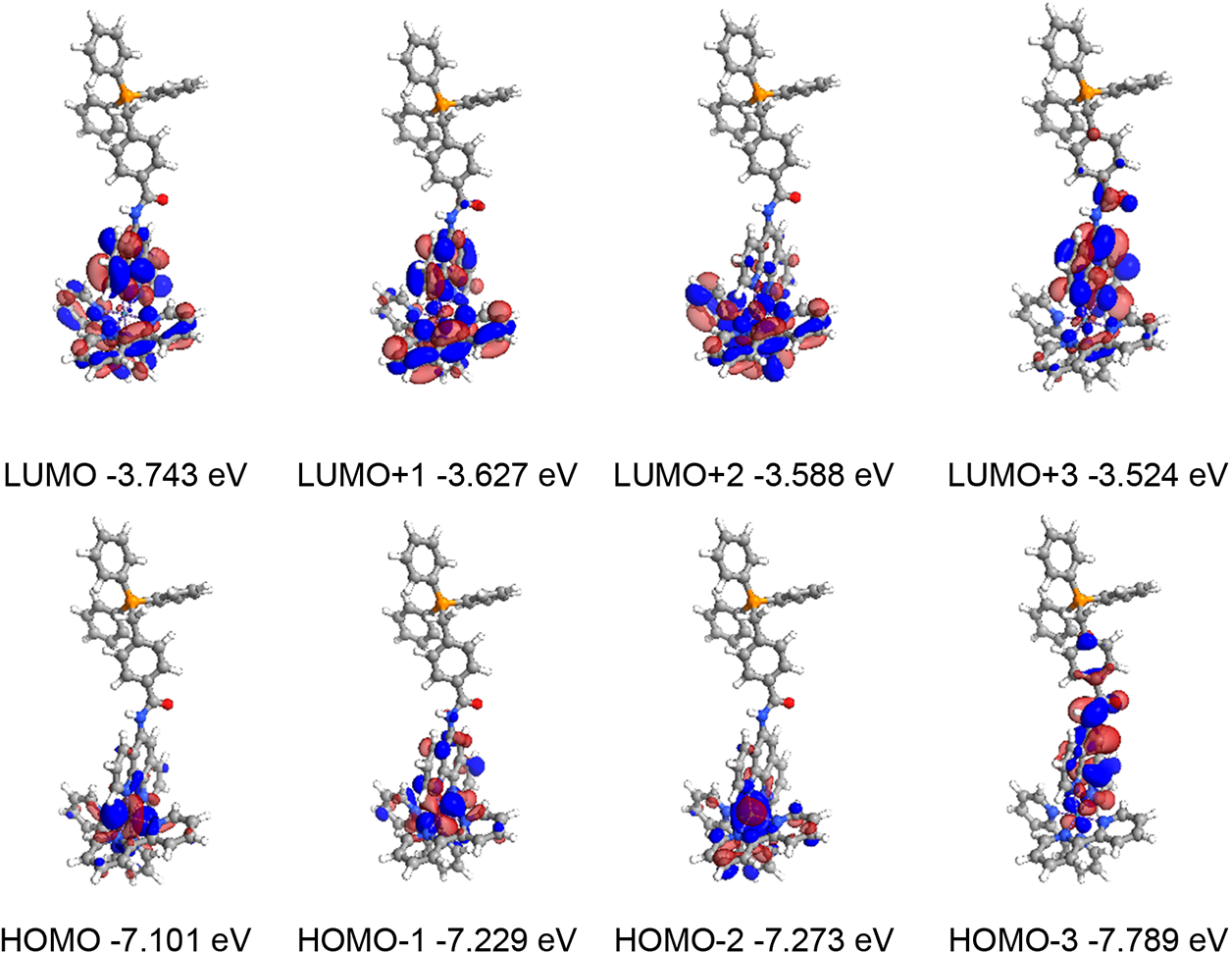
Calculated Kohn–Sham molecular orbitals for [Ru­(bipy)_2_(**L** ^**5**^)]^3+^.

**Table 4:** Selected Computed Values for the Various Energy Gaps Obtained from Vertical TD-DFT Calculations on the TPP^+^ Complexes

complex	S_0_ → S_1_/nm	T_1_ → S_0_/nm
[Ru(bipy)_2_(**L** ^**5**^)]^3+^	492	659
[Ru(bipy)_2_(**L** ^**6**^)]^3+^	496	665
[Ru(bipy)_2_(**L** ^**7**^)]^3+^	500	667
[Ru(bipy)_2_(**L** ^**8**^)]^3+^	491	678
[Ir(tmq)_2_(**L** ^**5**^)]^2+^	502	642
[Ir(tmq)_2_(**L** ^**6**^)]^2+^	505	675
[Ir(tmq)_2_(**L** ^**7**^)]^2+^	510	648
[Ir(tmq)_2_(**L** ^**8**^)]^2+^	493	645

The TD-DFT calculations for [Ir­(tmq)_2_(**L** ^**5–7**^)]^2+^ described a Ir­(5d) contribution to HOMO and HOMO – 1 which is less (32% or below) than in the Ru­(II) systems; the cyclometalating ligands (especially the phenyl components) become increasingly important to these orbitals (Tables 5, and S6–S8). The different LUMO, LUMO + 1 and LUMO + 2 levels (which localize on different combinations of phen, phen/tmq and tmq ligands, respectively) are predicted to be quite close in energy and thus important to the visible region excitations predicted at 462–510 nm, which compare nicely with experimental data; the overall analysis suggests combined MLCT and LLCT character to these excitations in [Ir­(tmq)_2_(**L** ^**5–7**^)]^2+^. [Ir­(tmq)_2_(**L** ^**8**^)]^2+^ also shares similar traits, with the variation in ligand structure not significantly impacting upon the important HOMO → LUMO transitions that are likely to dictate the optical properties of the complex.

**Table 5:** Description of the Calculated MO Contributions, Excited States Descriptions and Their Associated Transitions for [Ir­(tmq)_2_(**L** ^**5**^)]^2+^, Where X Corresponds to the Combined bipy and Phenanthroline Ligands, and Y from Branching Nitrogen on the Phenanthroline Onwards

	moiety contribution to orbital (%)			orbital contribution to excited state	
orbital	Ir (5d) (%)	X (%)	Y (%)	excited state	contributing transitions (>10%)
LUMO + 4	0	18	82	1 (502 nm *f* = 0.0413)	HOMO → LUMO (92.3%)
LUMO + 3	2	88	10	2 (476 nm *f* = 0.0177)	HOMO → LUMO + 1 (95.1%)
LUMO + 2	4	95	1		
LUMO + 1	2	88	10		
LUMO	4	95	1		
HOMO	31	69	0	3 (469 nm *f* = 0.0297)	HOMO → LUMO + 2 (95.1%)
HOMO – 1	23	77	0	4 (444 nm *f* = 0.0062)	HOMO–1 → LUMO (91.1%)
HOMO – 2	12	87	0	5 (430 nm *f* = 0.0247)	HOMO → LUMO + 3 (89.8%)
HOMO – 3	16	82	2	6 (421 nm *f* = 0.0285)	HOMO–1 → LUMO + 1 (88.6%)
HOMO – 4	22	72	6		

Again, none of the important MOs are located on the TPP^+^ fragment in these complexes. The calculated Kohn–Sham orbitals for [Ir­(tmq)_2_(**L** ^**5–8**^)]^2+^ are pictorially represented in Figures 9 and S73–75.

**Figure 9:**
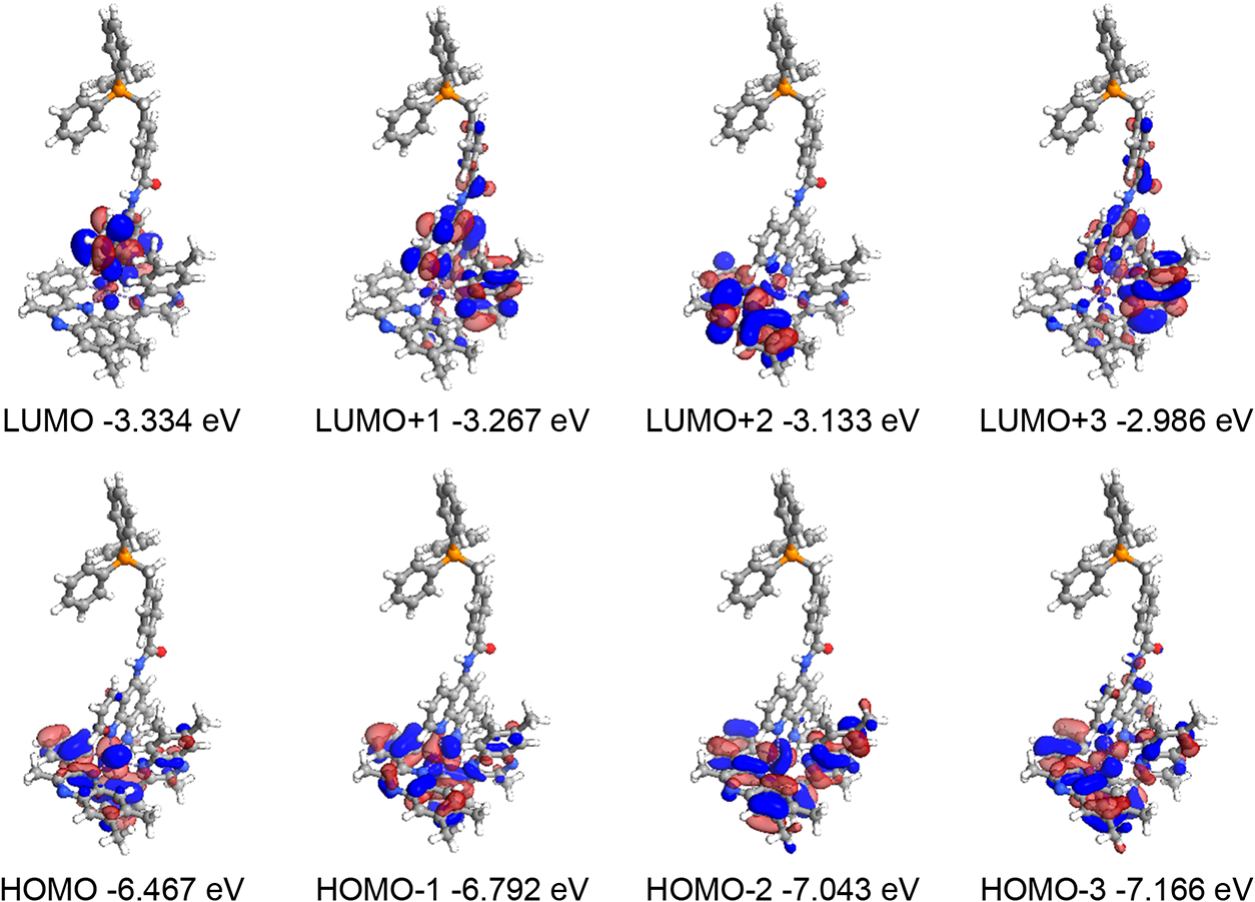
Calculated Kohn–Sham molecular orbitals for [Ir­(tmq)_2_(**L** ^**5**^)]^2+^.

The solution state photoluminescence properties (Table 6) of the Ru­(II) complexes show an emission maximum at 603–610 nm which was broad and structureless in appearance (Figure 10). The aerated emission lifetimes were in the range 0.132–0.147 μs and these extended to just below a microsecond upon degassing showing the sensitivity to dissolved oxygen and the triplet nature of the emission. Quantum yield values were generally consistent with related Ru­(II)-polypyridine species and were enhanced upon degassing, in a similar manner to that reported for [Ru­(bipy)_3_]­(PF_6_)_2_ (9.5% when degassed): these Ru­(II) complexes appear to be classical ^3^MLCT emitters. When comparing the degassed emission data of the complexes of **L** ^**1**^-**L** ^**4**^ with **L** ^**5**^-**L** ^**8**^, it was evident that the presence of the TPP^+^ unit results in a relative quenching of the ^3^MLCT state as evidenced by (an averaged) ∼ 25% reduction in lifetime for complexes of **L** ^**5**^-**L** ^**7**^ vs. **L** ^**1**^-**L** ^**3**^; in the more extended structure of **L** ^**8**^, where the TPP^+^ fragment is positioned further away from the coordination sphere, the quenching effect appeared less pronounced. It is noteworthy, therefore, that the calculated values of the nonradiative decay constant (*k*_nr_) are uniformly larger for the TPP^+^ complexes.

**Figure 10:**
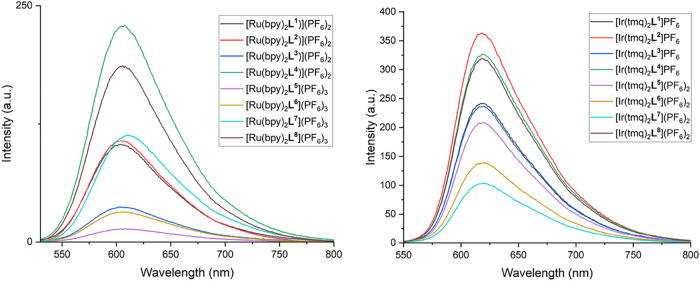
Steady state emission spectra (293 K, aerated 3.33 × 10^–6^ M MeCN, λ_ex_ = 450 nm) for the Ru­(II) (left) and Ir­(III) complexes.

**Table 6:** Photoluminescence Data for the Ru­(II) and Ir­(III) Complexes^a^

complex	emission, λ_em_/nm^b^	lifetime, τ/μs^c^	quantum yield, Φ/%^d^	*k*_r_/s^–1^	*k*_nr_/s^–1^
[Ru(bipy)_2_(**L** ^**1**^)][PF_6_]_2_	605	0.147 (0.806)	1.2 (5.0)	6.20 × 10^04^	1.18 × 10^06^
[Ru(bipy)_2_(**L** ^**2**^)][PF_6_]_2_	603	0.147 (0.901)	2.1 (11.0)	1.22 × 10^05^	9.88 × 10^05^
[Ru(bipy)_2_(**L** ^**3**^)][PF_6_]_2_	604	0.132 (0.809)	0.5 (4.0)	4.94 × 10^04^	1.19 × 10^06^
[Ru(bipy)_2_(**L** ^**4**^)][PF_6_]_2_	605	0.139 (0.930)	1.9 (13.0)	1.40 × 10^05^	9.35 × 10^05^
[Ru(bipy)_2_(**L** ^**5**^)][PF_6_]_3_	606	0.137 (0.639)	0.1 (1.8)	2.82 × 10^04^	1.54 × 10^06^
[Ru(bipy)_2_(**L** ^**6**^)][PF_6_]_3_	605	0.134 (0.666)	0.3 (3.0)	4.50 × 10^04^	1.46 × 10^06^
[Ru(bipy)_2_(**L** ^**7**^)][PF_6_]_3_	610	0.137 (0.571)	0.9 (4.0)	7.01 × 10^04^	1.68 × 10^06^
[Ru(bipy)_2_(**L** ^**8**^)][PF_6_]_3_	606	0.147 (0.842)	1.5 (15.0)	1.78 × 10^05^	1.01 × 10^06^
[Ir(tmq)_2_(**L** ^**1**^)]PF_6_	616	0.399 (2.568)	2.0 (21.0)	8.18 × 10^04^	3.08 × 10^05^
[Ir(tmq)_2_(**L** ^**2**^)]PF_6_	618	0.442 (2.347)	3.9 (37.0)	1.58 × 10^05^	2.68 × 10^05^
[Ir(tmq)_2_(**L** ^**3**^)]PF_6_	619	0.319 (2.463)	2.2 (29.0)	1.18 × 10^05^	2.88 × 10^05^
[Ir(tmq)_2_(**L** ^**4**^)]PF_6_	619	0.342 (2.927)	4.2 (56.0)	1.91 × 10^05^	1.50 × 10^05^
[Ir(tmq)_2_(**L** ^**5**^)][PF_6_]_2_	619	0.252 (1.319)	1.6 (17.0)	1.29 × 10^05^	6.29 × 10^05^
[Ir(tmq)_2_(**L** ^**6**^)][PF_6_]_2_	619	0.302 (1.713)	1.5 (18.0)	1.05 × 10^05^	4.79 × 10^05^
[Ir(tmq)_2_(**L** ^**7**^)][PF_6_]_2_	621	0.263 (1.678)	0.8 (16.0)	9.54 × 10^04^	5.01 × 10^05^
[Ir(tmq)_2_(**L** ^**8**^)][PF_6_]_2_	619	0.320 (2.642)	3.7 (66.0)	2.50 × 10^05^	1.29 × 10^05^

a All measurements obtained in MeCN at 293 K, 3.33 × 10^–6^ M solutions.

b Maximal phosphorescence emission wavelength.

c Phosphorescence lifetimes.

d Phosphorescence quantum yields (λ_ex_ = 450 nm); using [Ru­(bipy)_3_]­[PF_6_]_2_ in aerated MeCN (Φ = 0.018) or degassed MeCN (values in parentheses) as a reference (Φ = 0.095),^51^ errors are estimated at 15%. Estimates of *k*_r_ and *k*_nr_ from degassed data using *k*_r_ = Φ/τ and *k*_nr_ = (1 – Φ)/τ.

For the analogous series of Ir­(III) complexes a similar pattern emerges. First, all complexes were emissive in the red region (616–621 nm) with an unstructured, broad band; these species show a small bathochromic shift in emission versus the Ru­(II) series. The emission wavelengths are also red-shifted compared to the archetypal cationic [Ir­(ppy)_2_(bipy)]­PF_6_ (in aerated MeCN, λ_em_ = 602 nm),^45^ but closely comparable to benchmark compounds, such as [Ir­(tmq)_2_(bipy)]­PF_6_ (in aerated MeCN, λ_em_ = 617 nm, τ = 450 ns, Φ = 5.1%) suggesting that variation in the ancillary ligand does not strongly perturb the emission energy. This is reasonable as previous studies have consistently shown that the cyclometalating ligands dictate the emission character in 2-phenylquinoxaline complexes of Ir­(III).^37,52^ The luminescence lifetimes were noted in the range 0.252–0.442 μs and these extended into the microsecond domain upon degassing (e.g., 2.927 μs for [Ir­(tmq)_2_(**L** ^**4**^)]­PF_6_ was the longest recorded within the series). Lifetime values in cyclometalated Ir­(III) complexes can be highly sensitive to changes in ligand structure.^53^ The Ir­(III) complexes generally show slightly improved emission efficiency compared to the Ru­(II) analogues with quantum yield values of 0.8–4.2% when aerated and dramatically improved values when degassed. Again, these observations reveal the triplet nature of the emission throughout the series of Ir­(III) complexes which comprises a likely admixture of ^3^MLCT/^3^LLCT states. As in the Ru­(II) series, the emission data clearly establish that augmenting the structures with the TPP^+^ moiety leads to quenching of the emission (an average 35% reduction in lifetime was noted for the Ir­(III) complexes of **L** ^**5**^-**L** ^**7**^ vs. **L** ^**1**^-**L** ^**3**^); again, the calculated values of *k*_nr_ are typically larger for the TPP^+^ complexes. Akin to the Ru­(II) series, the extended ligand architecture of **L** ^**8**^ appeared to lessen quenching for [Ir­(tmq)_2_(**L** ^**8**^)]­(PF_6_)_2_ versus [Ir­(tmq)_2_(**L** ^**4**^)]­(PF_6_). Therefore, the emerging pattern for both series of complexes appears consistent in that augmenting the structures with the TPP^+^ unit can lead to a partial quenching of the emission, which can be negated by spatially distancing the TPP^+^ cation through structural alteration.

### A Photophysical Comparison of –PPh_3_^+^ versus –NEt_3_^+^ Derivatives

To further investigate the photophysical behavior of the TPP^+^ complexes, tetraalkylammonium analogues of **L** ^**7**^ were synthesized (see Experimental Section) to give two new cationic complexes, [Ru­(bipy)_2_(**L** ^**9**^)]­(PF_6_)_3_ and [Ir­(tmq)_2_(**L** ^**9**^)]­(PF_6_)_2_ (Figure 11). Steady state luminescence data showed that the change from –PPh_3_^+^ (**L** ^**7**^) to –NEt_3_^+^ (**L** ^**9**^) did not strongly influence λ_em_ in either acetonitrile (MeCN) or dichloromethane (DCM) solvent (Table 7).

**Figure 11:**
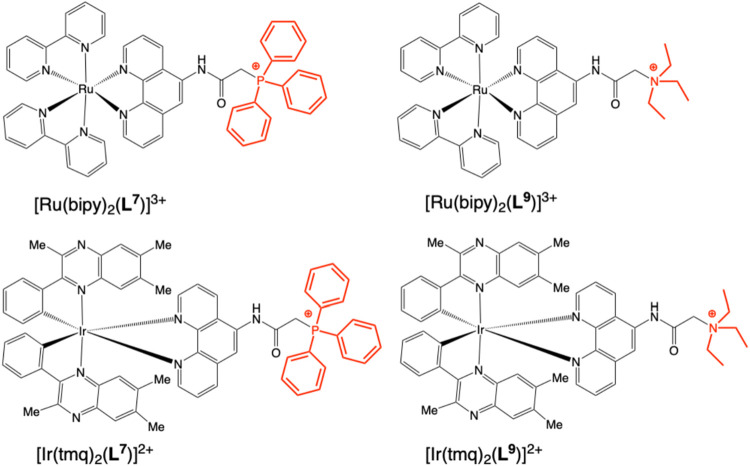
Comparison of the complex structures isolated for the phosphonium (**L** ^**7**^) versus the triethylammonium analogue (**L** ^**9**^).

**Table 7:** Photoluminescence Data Comparing –PPh_3_^+^ (**L** ^**7**^) to –NEt_3_^+^ (**L** ^**9**^) Derivatives in Both MeCN and DCM^a^

complex	emission, λ_em_/nm^b^	Lifetime, τ/μs^c^	quantum yield, Φ/%^d^
*In MeCN*			
[Ru(bipy)_2_(**L** ^**7**^)][PF_6_]_3_	610	0.137 (0.571)	0.9 (4.0)
[Ru(bipy)_2_(**L** ^**9**^)][PF_6_]_3_	614	0.148 (0.834)	0.9 (17.0)
[Ir(tmq)_2_(**L** ^**7**^)][PF_6_]_2_	621	0.263 (1.678)	0.8 (16.0)
[Ir(tmq)_2_(**L** ^**9**^)][PF_6_]_2_	621	0.294 (2.420)	0.8 (40.0)
*In DCM*			
[Ru(bipy)_2_(**L** ^**7**^)][PF_6_]_3_	595	0.302 (0.485)	3.8 (10.0)
[Ru(bipy)_2_(**L** ^**9**^)][PF_6_]_3_	591	0.313 (0.504)	4.9 (12.0)
[Ir(tmq)_2_(**L** ^**7**^)][PF_6_]_2_	615	0.579 (3.035)	5.7 (37.0)
[Ir(tmq)_2_(**L** ^**9**^)][PF_6_]_2_	615	0.608 (3.032)	6.1 (34.0)

a All measurements obtained at 293 K, 3.33 × 10^–6^ M solutions.

b Maximal phosphorescence emission wavelength.

c Phosphorescence lifetimes, degassed values in parentheses.

d Phosphorescence quantum yields (λ_ex_ = 450 nm) using [Ru­(bipy)_3_]­[PF_6_]_2_ in MeCN (Φ = 0.018, or Φ = 0.095 for degassed)^52^ and degassed values in parentheses, errors are estimated at 15%.

Within each pair of complexes, lifetime and quantum yield values in aerated solvent broadly sit within a ±10% range, implying that the extent of oxygen quenching is comparable in complexes of **L** ^**7**^ versus **L** ^**9**^ despite the bulkier TPP^+^ unit. In deoxygenated DCM the data also suggest that there was very little change in these photophysical parameters for **L** ^**7**^ versus **L** ^**9**^ (Table 7). The DCM data therefore shows that the TPP^+^ unit does not directly contribute to quenching of the emissive state in the Ru­(II) and Ir­(III) complexes. However, in deoxygenated MeCN the lifetimes for the –NEt_3_^+^ species [Ru­(bipy)_2_(**L** ^**9**^)]­(PF_6_)_3_ and [Ir­(tmq)_2_(**L** ^**9**^)]­(PF_6_)_2_ are longer (ca. 45%) and the quantum yields are higher. In this case, given that MeCN, as a more polar solvent, can more rapidly facilitate ion pair separation compared to DCM,^54^ it is possible that the photophysical behavior of these complexes is strongly influenced by the overall charge and thus intricate interplay of ion pairing and solvation as noted in other Ru­(II)-polypyridines.^55,56^

## Conclusion

This study has shown that the triphenylphosphonium moiety can be incorporated into a range of related ligand architectures based upon a functionalized 1,10-phenanthroline chelate. In so doing, a series of heteroleptic Ru­(II) and Ir­(III) complexes have been synthesized and fully characterized using a range of methods, including X-ray diffraction. Each of the complexes is photoluminescent in the visible red region at 603–621 nm, which is ascribed to a triplet excited state of significant ^3^MLCT (for the Ru­(II) species) or ^3^MLCT/^3^LLCT character (for the Ir­(III) species). Critically, the study shows that the presence of the pendant TPP^+^ moiety does not directly lead to quenching of the emissive states. However, under specific solvent conditions partial quenching can be observed where the linker unit is relatively short, which may relate to solvent dependent ion pairing phenomena. Therefore contributions to excited state quenching must be considered when TPP^+^ units are conjugated specific metal-based luminophores. Given the biological significance of the TPP^+^ moiety, and its demonstration in targeted bioimaging applications, the long-lived red emission characteristics of the series of complexes presented herein suggests significant promise. Future studies will focus upon the utility of the complexes, and related derivatives, as cellular imaging agents via confocal fluorescence microscopy.

## Experimental Section

^1^H, ^13^C­{^1^H} NMR spectra were recorded on an NMR-FT Bruker 500 MHz and spectrometer and recorded in CDCl_3_, methanol-*d_4_*, acetonitrile-*d*_3_ and acetone-*d*_6_. ^1^H and ^13^C­{^1^H} NMR chemical shifts (δ) were determined relative to residual solvent peaks with digital locking and are given in ppm. Coupling constants are quoted in Hz. High-resolution mass spectra were obtained by the staff at Cardiff University. UV–vis studies were performed on a Shimadzu UV-1800 spectrophotometer as MeCN solutions (3.3 × 10^–6^ M). Photophysical data were obtained on a JobinYvon–Horiba Fluorolog spectrometer fitted with a JY TBX picosecond photodetection module as MeCN or CH_2_Cl_2_ solutions. The pulsed source was a Nano-LED configured for 295 nm output operating at 1 MHz or 500 kHz. Luminescence lifetime profiles were obtained using the JobinYvon–Horiba FluoroHub single photon counting module and the data fits yielded the lifetime values using the provided DAS6 deconvolution software.

### Cyclic Voltammetry

Cyclic voltammetry was performed by using a PalmSens4 potentiostat. Experiments were performed using high-performance liquid chromatography-grade MeCN with an analyte concentration of 1 mM at 293 K using triply recrystallized [*n*Bu_4_N]­[PF_6_] as the supporting electrolyte at 0.1 M concentration. A three-electrode setup was used, consisting of a platinum disc working electrode, a platinum wire counter electrode, and a silver wire pseudo reference. Solutions were sparged for 10 min with MeCN-saturated stream of nitrogen gas. Voltammograms were referenced to the ferrocene/ferrocenium redox couple measured using the same conditions.

### X-ray Crystallography

#### Data Collection and Processing

Suitable crystals of **L** ^**5**^(*I*_*3*_), **L** ^**5**^(*ICl*), [Ru­(bipy)_2_(**L** ^**5**^)]­[PF_6_]_3_ and [Ir­(tmq)_2_(**L** ^**6**^)]­[PF_6_]_2_ were selected and data collected following a standard method.^57^ For each a suitable crystal was selected and mounted on a MITIGEN holder in oil on a Rigaku FRE+ diffractometer with Arc)­Sec VHF Varimax confocal mirrors, a UG2 goniometer and HyPix 6000HE detector. The crystal was kept at a steady *T* = 100(2) K during data collection. The structures were solved with the ShelXT^58^ structure solution program using the Intrinsic Phasing solution method and by using Olex2 ^59^ as the graphical interface. The model was refined with version 2018/3 of ShelXL^60^ using Least Squares minimization. CCDC 2492252–2492255 contains supplementary X-ray crystallographic data for **L** ^**5**^(*I*_*3*_), **L** ^**5**^(*ICl*), [Ru­(bipy)_2_(**L** ^**5**^)]­[PF_6_]_3_ and [Ir­(tmq)_2_(**L** ^**6**^)]­[PF_6_]_2_ respectively. This data can be obtained free of charge via http://www.ccdc.cam.ac.uk/conts/retrieving.html, or from the Cambridge Crystallographic Data Centre, Union Road, Cambridge, CB2 1EZ; fax­(+44) 1223–336–033 or email: deposit@ccdc.cam.ac.uk.

#### Computational Methods

Electronic structure calculations were performed using density functional theory within the ORCA 6.0 software package.^61^ All calculations were performed using the B3LYP functional with Grimme’s D3 (BJ) dispersion correction and the def2-TZVP basis set, employing the conductor-like polarizable continuum model (CPCM) to simulate solvent effects.

All geometry optimizations were performed using DEFGRID3 integration grid, and tight convergence criteria. Ground-state (S_0_) optimizations were confirmed to correspond to true energy minima through harmonic vibrational frequency calculations within ORCA, with no imaginary frequencies observed. The optimized S_0_ geometries were subsequently used in single-point time-dependent DFT (TD-DFT) calculations to compute vertical excitation energies, as well as for the optimization of the first singlet (S_1_), and first triplet (T_1_) excited state geometries. Optimized S_1_ states represent true energy minima show very little difference when overlaid with their respective S_0_ geometries (Table S9). Excitation spectra were computed over 10,000 points, using a Gaussian line shape in the orca_mapspc program.

Due to the absence of analytical frequency methods for excited states in ORCA, direct verification of these geometries was computationally intractable. Instead S_1_ geometries were further validated by reoptimization and vibrational frequency analysis in the Gaussian 09 software package,^62^ again confirming the absence of imaginary frequencies. The optimized geometries obtained from ORCA and Gaussian 09 were confirmed to be identical through structural overlap analysis using the ChimeraX software package.^63^

Phosphorescence properties were investigated using unrestricted density functional theory, to characterize the first triplet state (T_1_), using identical conditions to those applied to the singlet states. The T_1_ states were confirmed to correspond to true energy minima through harmonic vibrational frequency calculations within ORCA, with no imaginary frequencies observed. Decomposition of the molecular orbital character was performed using the MultiWFN software.^64^ The superposition of the singlet and triplet geometries for all complexes was carried out using ChimeraX.

### Synthesis of Ligands

#### Synthesis of 4-Chloromethyl-*N*-(1,10-phenanthrolin-5-yl)­benzamide (**L^1^**)

5-amino-1,10-phenanthroline (0.2 g, 0.76 mmol) and 4-chloromethyl-benzoyl chloride (0.143 g, 0.76 mmol) were combined and dissolved in DCM (15 mL). The mixture was heated to reflux for 24 h. Once cooled, the solvent was removed under vacuum and the residue was dissolved in MeOH (5 mL) and Et_2_O (15 mL) was added, the precipitated was retrieved through filtration and washed with Et_2_O (3 × 5 mL). This yielded a crystalline yellow powder (1.5 g, 84%). ^1^H NMR (300 MHz, CD_3_OD, 298 K) δ_H_ (ppm): 9.29 (1H, dd, *J* = 4.7, 1.5 Hz), 9.22 (1H, dd, *J* = 5.1, 1.5 Hz), 9.04 (1H, dd, *J* = 8.4, 1.5 Hz), 8.95 (1H, dd, *J* = 8.6, 1.5 Hz), 8.46 (1H, s), 8.21 (1H, dd, *J* = 8.3, 5.1 Hz), 8.17–8.11 (3H, m), 7.69–7.64 (2H, m), 4.77 (2H, s). FTIR (solid, ATR) ν/cm^–1^: 3275, 3102, 3032, 2969, 2565, 2365, 16545, 1628, 1612, 1595, 1558, 1541, 1526, 1491, 1479, 1420, 1391, 1325, 1304, 1279, 1244, 1182, 1132, 1028, 1016, 920, 887, 872, 829, 808, 766, 727, 706, 689, 664, 633, 623, 588, 538, 503, 482, 447, 434, 420, 411, 401. UV–vis (CH_3_OH): λ_max_/nm (ε/L mol^–1^cm^–1^): 232 (34882), 269 (30489), 310 (6520). HRMS (ES+) found *m*/*z* 348.0905 [M + H]^+^, calculated *m*/*z* 348.0904 for [C_20_H_15_N_3_OCl]

#### Synthesis of 3-Chloro-*N*-(1,10-phenanthrolin-5-yl)­propenamide (**L^2^**)

5-amino-1,10-phenanthroline (0.5 g, 2.56 mmol) and triethylamine (0.4 mL, 2.56 mmol) were suspended in dry THF (30 mL). 3-chloropropionyl chloride (0.244 mL, 2.56 mmol) was suspended in dry THF (2 mL) and added dropwise. The reaction mixture was stirred overnight at room temperature. The white precipitate was retrieved through vacuum filtration and washed with deionized water. The product was further purified via a gradient flash column chromatography (DCM:MeOH; 100:0, 99:1, 97:3, 95:5). The product was obtained as resin-like orange solid. (0.582 g, 80%). ^1^H NMR (300 MHz, CD_3_OD, 298 K) δ_H_ (ppm): 9.17 (1H, dd, *J* = 4.6, 1.5 Hz), 9.08 (1H, dd, *J* = 5.0, 1.5 Hz), 8.82 (2H, ddd, *J* = 9.9, 8.4, 1.5 Hz), 8.31 (1H, s), 8.02 (2H, app. td, *J* = 8.1, 4.8 Hz), 4.01 (2H, t, *J* = 6.2 Hz), 3.15 (2H, t, J = 6.2 Hz). ^13^C­{^1^H} NMR (101 MHz, CD_3_OD, 298 K) δ_C_ (ppm): 150.8, 150.5, 137.7, 133.3, 124.8, 124.3, 122.3, 40.9 (*C*H_2_), 40.1 (*C*H_2_). H FTIR (solid, ATR) ν/cm^–1^: 3362, 3208, 2978, 2604, 2531, 2496, 1667, 1624, 1589, 1537, 1477, 1445, 1422, 1396, 1385, 1317, 1229, 1198, 1173, 1152, 1111, 1070, 1036, 986, 874, 851, 826, 804, 739, 656, 623, 471, 463, 438, 420, 409. UV–vis (CH_3_OH): λ_max_/nm (ε/L mol^–1^cm^–1^): 229 (12732), 232 (12727), 270 (11136), 312 (2270). HRMS (ES+) found *m*/*z* 286.0751 [M + H]^+^, calculated *m*/*z* 286.0747 for [C_15_H_13_N_3_OCl]

#### Synthesis of 2-Chloro-*N*-(1,10-phenanthrolin-5-yl)­acetamide (**L^3^**)

Using an adapted method,^65^ 5-amino-1,10-phenanthroline (0.3 g, 1.5 mmol) and triethylamine (0.209 mL, 1.5 mmol) were suspended in dry MeCN (20 mL) and cooled in an ice bath. Chloroacetyl chloride (0.122 mL, 1.5 mmol) was added dropwise to the suspension and stirred for 5 h at room temperature over which time a white precipitate was formed. The precipitate was retrieved through filtration under vacuum and washed with MeCN (3 × 10 mL). The product was obtained as a white powder (0.331g, 81%).^1^H NMR (300 MHz, CDCl_3_, 298 K) δ_H_ (ppm): 9.22 (1H, dd, *J* = 4.3, 1.6 Hz), 9.15 (1H, dd, *J* = 4.3, 1.7 Hz), 8.98 (1H, s), 8.36–8.27 (2H, m), 8.24 (1H, dd, *J* = 8.2, 1.7 Hz), 7.70 (1H, dd, *J* = 8.4, 4.3 Hz), 7.64 (1H, dd, *J* = 8.1, 4.3 Hz), 4.41 (2H, s). ^13^C­{^1^H} NMR (101 MHz, CDCl_3_, 298 K) δ_C_ (ppm): 150.4, 150.2, 136.1, 129.1, 123.6, 123.1, 119.7, 43.5 (*C*H_2_). FTIR (solid, ATR) ν/cm^–1^: 3225, 3186, 3148, 3067, 3042, 3005, 1684, 1622, 1589, 1566, 1537, 1508, 1479, 1456, 1422, 1408, 1387, 1317, 1304, 1288, 1267, 1250, 1227, 1217, 1153, 1130, 1109, 1067, 974, 943, 922, 903, 895, 835, 824, 804, 795, 739, 714, 652, 635, 625, 604, 571, 561, 515, 463, 424, 419, 413. UV–vis (CH_3_OH): λ_max_/nm (ε/L mol^–1^cm^–1^): 228(25079), 233 (25758), 269 (21679), 314 (3537). HRMS (ES+) found *m*/*z* 272.0595 [M + H]^+^, calculated *m*/*z* 272.0591 for [C_14_H_11_N_3_OCl].

#### Synthesis of 1-((1,10-Phenanthrolin-5-yl)­piperazin-1-yl)­(4-(chloromethyl)­benzoyl (**L^4^**)

5-(piperazin-1-yl)-1,10-phenthanthroline (0.2 g, 0.76 mmol) and 4-chloromethyl-benzoyl chloride (0.143 g, 0.76 mmol) were dissolved in DCM (15 mL). The mixture was heated to reflux for 24 h. Once cool, the solvent was removed under vacuum and the residue was redissolved in MeOH (5 mL). Et_2_O (15 mL) was added, and the resultant precipitate was retrieved through filtration and washed with Et_2_O (3 × 5 mL) yielding a crystalline brown-orange powder (0.128 g, 40%). ^1^H NMR (300 MHz, CD_3_OD, 298 K) δ_H_ (ppm): 9.26 (1H, dd, *J* = 4.7, 1.5 Hz), 9.09 (1H, dd, *J* = 5.2, 1.5 Hz), 9.06 (1H, dd, *J* = 8.5, 1.5 Hz), 8.99 (1H, dd, *J* = 8.4, 1.4 Hz), 8.15 (2H, ddd, *J* = 14.2, 8.4, 4.9 Hz), 7.80 (1H, s), 7.60–7.51 (4H, m), 4.7 (2H, s), 4.1 (2H, br. s), 3.8 (2H, br. s), 3.32 (4 H, br. s).^13^C­{^1^H} NMR (101 MHz, CD_3_OD, 298 K) δ_C_ (ppm): 148.3, 143.3, 139.8, 133.2, 127.3, 126.0, 123.4, 123.2, 111.3, 51.3 (*C*H_2_), 43.8 (*C*H_2_). FTIR (solid, ATR) ν/cm^–1^: 3368, 3096, 3057, 2907, 2826, 1611, 1591, 1541, 1499, 1458, 1437, 1339, 1279, 1263, 1213, 1180, 1157, 1128, 1086, 1053, 1005, 912, 852, 808, 779, 727, 660, 621, 457, 441, 432, 420, 409. UV–vis (CH_3_OH): λ_max_/nm (ε/L mol^–1^cm^–1^): 229 (45807), 278 (19010), 316 (5315). HRMS (ES+) found *m*/*z* 417.1481 [M + H]^+^, calculated *m*/*z* 417.1482 for [C_24_H_22_N_4_OCl].

#### Synthesis of (4-((1,10-Phenanthrolin-5-yl)­carbamoyl)­benzyl)­triphenylphosphonium Iodide (**L^5^**)

**L** ^**1**^ (0.2 g, 0.58 mmol), triphenylphosphine (0.151 g, 0.58 mmol) and potassium iodide (0.096 g, 0.58 mmol) were combined in degassed MeCN (15 mL) and heated to reflux for 24 h. During this time a precipitate formed and was retrieved through filtration and washed with MeCN (3 × 10 mL) to give an orange resin-like solid (0.389 g, 97%). ^1^H NMR (300 MHz, CD_3_OD, 298 K) δ_H_ (ppm): 9.31 (1H, dd, *J* = 4.7, 1.5 Hz), 9.24 (1H, dd, *J* = 5.1, 1.5 Hz), 9.07 (1H, dd, *J* = 8.4, 1.5 Hz), 8.96 (1H, dd, *J* = 8.5, 1.5 Hz), 8.48 (1H, s), 8.23 (1H, dd, *J* = 8.3, 5.1 Hz), 8.16 (1H, dd, *J* = 8.5, 4.8 Hz), 8.05–7.99 (2H, m), 7.98–7.90 (3H, m), 7.83–7.70 (12 H, m), 7.28 (2H, dd, *J* = 8.5, 2.5 Hz), 5.16 (2H, d). ^13^C­{^1^H} NMR (101 MHz, CD_3_OD, 298 K) δ_C_ (ppm): 169.0, 149.9, 147.0, 144.2, 140.1, 138.1, 137.7, 136.7, 136.6, 135.5, 135.4, 135.2, 133.9, 133. 8, 132.73, 132.68, 131.6, 131.5, 131.3, 129.79, 129.76, 128.4, 126.8, 126.7, 123.3, 119.3, 118.5, 31.0, 30.5. ^31^P­{^1^H} NMR (162 MHz, MeOD) δ_P_ (ppm): 22.97. FTIR (solid, ATR) ν/cm^–1^: 3649, 6429, 3379, 3169, 2980, 2903, 2847, 2779, 1661, 1612, 1595, 1535, 1520, 1499, 1483, 1454, 1437, 1416, 1400, 1381, 1368, 1327, 1279, 1252, 1238, 1207, 1157, 1111, 1032, 995, 951, 897, 874, 862, 829, 818, 808, 783, 752, 725, 718, 691, 635, 890, 557, 530, 498, 474, 440, 419. UV–vis (CH_3_OH): λ_max_/nm (ε/L mol^–1^cm^–1^): 226 (60208), 269 (32167), 270 927706), 313 (6201). HRMS (ES+) found *m*/*z* 574.2048 [M]^+^, calculated *m*/*z* 574.2048 for [C_38_H_29_N_3_OP].

#### Synthesis of (3-((1,10-Phenanthrolin-5-yl)­carbamoyl)­propyl)­triphenylphosphonium Iodide (**L^6^**)

As for **L** ^**5**^, but using **L** ^**2**^ (0.2 g, 0.7 mmol), triphenylphosphine (0.184 g, 0.7 mmol) and potassium iodide (0.116 g, 0.7 mmol). The product was retrieved as a pink powder (0.130 g, 45%). ^1^H NMR (300 MHz, CDCl_3_) δ_H_ (ppm): 10.35 (1H, s), 9.17–9.09 (3H, m), 8.16 (1H, dd, *J* = 8.2, 1.7 Hz), 8.11 (1H, s), 7.86–7.66 (16H, m), 7.61 (1H, dd, *J* = 8.1, 4.4 Hz), 7.32 (1H, d, *J* = 1.8 Hz), 3.86–3.72 (2H, m), 3.70–3.56 (2H, m). ^13^C­{^1^H} NMR (101 MHz, CDCl_3_, 298 K) δ_C_ (ppm): 149.8, 148.9, 136.6, 135.5, 135.5, 133.73, 133.65, 133.57, 132.04, 131.97, 130.8, 130.7, 128.5, 128.4, 123.4, 123.1, 119.3, 50.8. ^31^P­{^1^H} NMR (202 MHz, CDCl_3_) δ_P_ (ppm): 24.67. FTIR (solid, ATR) ν/cm^–1^: 3645, 3418, 3213, 3171, 2988, 2870, 2799, 1668, 1622, 1587, 1531, 1506, 1481, 1437, 1422, 1404, 1381, 1335, 1345, 1260, 1223, 1206, 1165, 1146, 1113, 1072, 1028, 997, 974, 897, 887, 833, 824, 810, 764, 748, 737, 725, 714, 689, 559, 523, 507, 490, 449, 442, 436, 420, 411. UV–vis (CH_3_OH): λ_max_/nm (ε/L mol^–1^cm^–1^): 225 (46769), 270 (21703), 275 (20822), 313 (4294). HRMS (ES+) found *m*/*z* 512.1891 [M]^+^, calculated *m*/*z* 512.1892 for [C_33_H_27_N_3_OP].

#### Synthesis of (2­((1,10-Phenanthrolin-5-yl)­carbamoyl)­methyl)­triphenylphosphonium Iodide (**L^7^**)

As for **L** ^**5**^, but using **L** ^**3**^ (0.1 g, 0.37 mmol), triphenylphosphine (0.0965 g, 0.37 mmol) and potassium iodide (0.061 g, 0.37 mmol). The product was retrieved as an orange resin-like solid (0.124 g, 54%). ^1^H NMR (500 MHz, CD_3_OD, 298 K) δ_H_ (ppm): 9.09 (1H, dd, *J* = 4.4, 1.6 Hz), 9.03 (1H, dd, *J* = 4.4, 1.7 Hz), 8.37–8.31 (1H, m), 8.29 (1H, dd, *J* = 8.4, 1.6 Hz), 8.00–7.87 (10H, m), 7.80–7.70 (8H, m), 4.70–4.50 (2H, br). ^13^C­{^1^H} NMR (126 MHz, CD_3_OD, 298 K) δ_C_ (ppm): 150.9, 150.7, 137.5, 136.21, 136.19, 135.05, 134.96, 132.6, 131.2, 131.1, 124.9, 124.2, 122.4. ^31^P­{^1^H} NMR (202 MHz, MeOD, 298 K) δ_P_ (ppm): 21.87. FTIR (solid, ATR) ν/cm^–1^: 3429, 3171, 3057, 2990, 1679, 1624, 1587, 1541, 1508, 1491, 1456, 1437, 1422, 1387, 1317, 1223, 1190, 1111, 1028, 997, 168, 901, 883, 854, 804, 739, 718, 687, 625, 540, 500, 467, 434, 413, 405. UV–vis (CH_3_OH): λ_max_/nm (ε/L mol^–1^cm^–1^): 226 (49798), 269 (23582), 274 (21372), 298 (8081), 315 (4510), 359 (1455). HRMS (ES+) found *m*/*z* 498.1736 [M]^+^, calculated *m*/*z* 498.1735 for [C_32_H_25_N_3_OP].

#### Synthesis of (4-(4-(1,10-Phenanthrolin-5-yl)­piperazine-1 carbamoyl)­benzyl)­triphenylphosphonium Iodide (**L^8^**)

As for **L** ^**5**^, but using **L** ^**4**^ (0.07 g, 0.17 mmol), triphenylphosphine (0.044 g, 0.17 mmol) and potassium iodide (0.028 g, 0.17 mmol). A reprecipitation from MeOH and Et_2_O was performed to give an orange resin-like solid (0.129 g, 53%). ^1^H NMR (400 MHz, CD_3_OD, 298 K) δ_H_ (ppm): 9.25 (1H, dd, *J* = 4.7, 1.5 Hz), 9.11–9.02 (2H, m), 8.97 (1H, dd, *J* = 8.4, 1.5 Hz), 8.12 (2H, td, *J* = 8.5, 4.9 Hz), 7.96–7.86 (3H, m), 7.80 (1H, s), 7.79–7.65 (12H, m), 7.39 (2H, d, *J* = 7.9 Hz), 7.17 (2H, dd, *J* = 8.2, 2.5 Hz), 5.06 (2H, d, *J* = 15.2 Hz), 4.11 (2H, s), 3.78 (2H, s), 3.35 (4H, d, *J* = 0.5 Hz). ^13^C­{^1^H} NMR (101 MHz, CD_3_OD, 298 K) δ_C_ (ppm): 149.8, 144.8, 143.1, 137.2, 136.31, 136.28, 135.2, 135.1, 132.4, 132.32, 131.26, 131.1, 128.61, 128.58, 126.3, 126.1, 114.3, 66.6. ^31^P­{^1^H} NMR (162 MHz, MeOD, 298 K) δ_P_ (ppm): 23.07. FTIR (solid, ATR) ν/cm^–1^: 1684, 1541, 1522, 1508, 1466, 1447, 1423, 1387, 1315, 1271, 1242, 1161, 1111, 835, 762, 741, 731, 723, 689, 662, 648, 556, 471, 451, 436, 424, 411, 405. UV–vis (CH_3_OH): λ_max_/nm (ε/L mol^–1^cm^–1^): 227 (30295), 270 (7898), 277 (8457), 286 (7062), 323 (2218). HRMS (ES+) found *m*/*z* 643.2630 [M]^+^, calculated *m*/*z* 643.2627 for [C_42_H_36_N_4_OP].

### Complex Synthesis

#### General Procedure for the Synthesis of Ru­(II) Complexes

Ru­(bipy)_2_Cl_2_ (1 equiv) and ligand (1 equiv) were combined in EtOH. N_2_ gas was bubbled through the solution for 30 min after which time the solution was heated to reflux for varying times as required. The reaction mixture was then concentrated under vacuum and a saturated solution of NH_4_PF_6_ was added to the solution. The product was then extracted into DCM and washed with deionized water (3 × 10 mL). The DCM was dried over MgSO_4_ and filtered. The filtrate was taken and the solvent removed under vacuum to yield the complex as an orange solid.

##### Synthesis of [Ru­(bipy)_2_(L^1^)]­(PF_6_)_2_

Ru­(bipy)_2_Cl_2_ (0.046 g, 0.1 mmol), **L** ^**1**^ (0.033 g, 0.1 mmol) and EtOH (10 mL) were heated for 16 h. MeCN:Et_2_O reprecipitation was performed to yield the product as an orange solid (0.052 g, 52%). ^1^H NMR (500 MHz, (CD_3_)_2_CO, 298 K) δ_H_ (ppm): 9.91 (1H, s), 9.00 (1H, ddd, *J* = 9.8, 8.6, 1.2 Hz), 8.87–8.79 (4H, m), 8.79–8.74 (2H, m), 8.47 (1H, ddd, *J* = 5.2, 2.5, 1.1 Hz), 8.35 (1H, app. dt, *J* = 5.2, 1.5 Hz), 8.25 (2H, app. td, *J* = 8.1, 1.7 Hz), 8.18–8.12 (4H, m), 7.96–7.87 (5H, m), 7.65–7.61 (2H, m), 7.42–7.36 (2H, m), 4.01 (2H, t, *J* = 6.2 Hz), 3.18 (2H, t, *J* = 6.2 Hz). ^13^C­{^1^H} NMR (126 MHz, (CD_3_)_2_CO, 298 K) δ_C_ (ppm): 153.4, 152.72, 152.70, 152.3, 138.7, 138.6, 137.2, 133.0, 128.49, 128.47, 128.4, 128.3, 127.2, 126.5, 125.1, 125.05, 124.98, 40.8, 40.0. FTIR (solid, ATR) ν/cm^–1^: 1674, 1634, 1605, 1506, 1466, 1447, 1423, 1385, 1314, 1273, 1163, 1018, 835, 762, 725, 648, 557, 420, 415, 407. UV–vis (CH_3_CN): λ_max_/nm (ε/L mol^–1^cm^–1^): 244 (55284), 256 (51744), 279 (78187), 286 (80862), 331 (16072), 387 (12436), 425 (20390), 450 (19949). HRMS (ES+) found *m*/*z* 380.5529 [M – 2PF_6_]^2+^, calculated *m*/*z* 380.5626 for [C_40_H_30_ClN_7_ORu]^2+^.

##### Synthesis of [Ru­(bipy)_2_(L^2^)]­(PF_6_)_2_

Ru­(bipy)_2_Cl_2_ (0.049 g, 0.1 mmol), **L** ^**2**^ (0.029 g, 0.1 mmol) and EtOH (10 mL) were heated for 16 h. An additional MeCN:Et_2_O reprecipitation was performed to yield the product as an orange solid (0.041 g, 41%). ^1^H NMR (500 MHz, (CD_3_)_2_CO, 298 K) δ_H_ (ppm): 10.24 (1H, s), 9.02 (1H, dd, *J* = 8.5, 1.2 Hz), 8.85 (2H, app. ddt, *J* = 8.2, 2.4, 1.1 Hz), 8.81 (2H, app. ddt, *J* = 8.2, 3.8, 1.1 Hz), 8.78 (1H, dd, *J* = 8.3, 1.2 Hz), 8.71 (1H, s), 8.47 (1H, dd, *J* = 5.2, 1.1 Hz), 8.38 (1H, dd, *J* = 5.2, 1.2 Hz), 8.25 (2H, app. tt, *J* = 7.8, 1.6 Hz), 8.22–8.12 (6H, m), 7.95–7.89 (4H, m), 7.71–7.67 (2H, m), 7.63 (2H, app. ddd, *J* = 7.3, 5.7, 1.3 Hz), 7.44–7.39 (2H, m), 4.84 (2H, s). ^13^C­{^1^H} NMR (126 MHz, (CD_3_)_2_CO, 298 K) δ_C_ (ppm): 153.5, 152.7, 152.64, 152.57, 138.7, 138.6, 137.2, 134.0, 129.63, 129.61, 129.0, 128.5, 128.4, 128.3, 127.2, 126.4, 125.07, 125.06, 124.99, 122.1, 45.8. FTIR (solid, ATR) ν/cm^–1^: 3645, 3401, 3250, 3096, 1686, 1630, 1605, 1582, 1533, 1481, 1466, 1447, 1423, 1315, 1242, 1161, 833, 762, 741, 727, 660, 648, 556, 469, 453, 444, 430, 401. UV–vis (CH_3_CN): λ_max_/nm (ε/L mol^–1^cm^–1^): 209 (34552), 245 (35707), 251 (35763), 283 (61476), 285 (62841), 322 (12897), 383 (8465), 425 (14539), 452 (16148). HRMS (ES+) found *m*/*z* 349.5461 [M – 2PF_6_]^2+^, calculated *m*/*z* 349.5547 for [C_35_H_28_ClN_7_ORu]^2+^.

##### Synthesis of [Ru­(bipy)_2_(L^3^)]­(PF_6_)_2_

Ru­(bipy)_2_Cl_2_ (0.045 g, 0.1 mmol), **L** ^**3**^ (0.025 g, 0.1 mmol) and EtOH (10 mL) were heated for 16 h. An additional MeCN:Et_2_O reprecipitation was performed to yield the product as an orange solid (0.042 g, 42%). ^1^H NMR (500 MHz, (CD_3_)_2_CO, 298 K) δ_H_ (ppm): 10.07 (1H, s), 8.93 (1H, dd, *J* = 8.6, 1.2 Hz), 8.84 (2H, app. ddt, *J* = 8.2, 2.1, 1.0 Hz), 8.82–8.76 (3H, m), 8.70 (1H, s), 8.47 (1H, dd, *J* = 5.2, 1.1 Hz), 8.37 (1H, dd, *J* = 5.2, 1.2 Hz), 8.27–8.23 (2H, m), 8.18–8.12 (4H, m), 7.94 (1H, dd, *J* = 8.5, 5.2 Hz), 7.92–7.88 (3H, m), 7.63 (2H, ddd, *J* = 7.2, 5.6, 1.3 Hz), 7.42–7.36 (2H, m), 4.53 (2H, d, *J* = 1.0 Hz).^13^C­{^1^H} NMR (126 MHz, (CD_3_)_2_CO, 298 K) δ_C_ (ppm): 153.5, 152.72, 152.69, 152.59, 138.8, 138.6, 137.2, 132.9, 128.48, 128.47, 128.4, 128.3, 127.2, 126.6, 125.07, 125.05, 124.99, 124.97, 121.1, 43.8. FTIR (solid, ATR) ν/cm^–1^: 3394, 3093, 1682, 1647, 1634, 1601, 1558, 1541, 1526, 1506, 1485, 1464, 1466, 1425, 1314, 1269, 1242, 1182, 1165, 1107, 836, 804, 760, 723, 662, 555, 496, 424, 422. UV–vis (CH_3_CN): λ_max_/nm (ε/L mol^–1^cm^–1^): 212 (31979), 245 (34877), 253 (32830), 279 (56239), 284 (59704), 328 (11676), 385 (11837), 422 (14396), 450 (14530). HRMS (ES+) found *m*/*z* 830.0571 [M - PF_6_]^+^, calculated *m*/*z* 830.0573 for [C_34_H_26_ClN_7_OF_6_PRu]^+^.

##### Synthesis of [Ru­(bipy)_2_(L^4^)]­(PF_6_)_2_

Ru­(bipy)_2_Cl_2_ (0.058 g, 0.1 mmol), **L** ^**4**^ (0.05 g, 0.1 mmol) and EtOH (10 mL) were heated for 16 h. An additional MeCN:Et_2_O reprecipitation was performed to yield the product as an orange solid (0.041 g, 41%). ^1^H NMR (500 MHz, (CD_3_)_2_CO, 298 K) δ_H_ (ppm): 8.76 (1H, dd, *J* = 8.5, 1.3 Hz), 8.55–8.47 (4H, m), 8.44 (1H, dd, *J* = 8.3, 1.2 Hz), 8.11–8.07 (2H, m), 8.05 (1H, dd, *J* = 5.2, 1.2 Hz), 7.99 (2H, td, *J* = 7.9, 1.5 Hz), 7.93–7.90 (1H, m), 7.86–7.80 (2H, m), 7.71 (1H, dd, *J* = 8.5, 5.2 Hz), 7.68 (1H, s), 7.63 (1H, dd, *J* = 8.3, 5.2 Hz), 7.56 (2H, app. dddd, *J* = 5.7, 2.3, 1.5, 0.8 Hz), 7.54–7.40 (6H, m), 7.24 (2H, app. dddd, *J* = 7.7, 5.8, 4.6, 1.3 Hz), 4.52 (1H, s), 4.11–3.58 (4H, m), 3.40 (4H, s), 1.20 (2H, t, *J* = 7.0 Hz), 1.12 (1H, t, *J* = 7.0 Hz). ^13^C­{^1^H} NMR (126 MHz, (CD_3_)_2_CO, 298 K) δ_C_ (ppm): 153.1, 152.83, 152.77, 152.75, 152.70, 151.3, 138.6, 138.52, 138.50, 136.5, 134.4, 129.7, 128.39, 128.36, 128.35, 128.29, 128.2, 128.0, 127.0, 126.3, 125.07, 125.04, 124.98, 115.1, 53.8 (*C*H_2_), 45.4 (*C*H_2_). FTIR (solid, ATR) ν/cm^–1^: 3649, 1618, 1514, 1466, 1447, 1387, 1283, 1256, 1229, 1161, 1096, 1011, 835, 764, 731, 648, 557, 521, 446, 436, 426, 419, 407. UV–vis (CH_3_CN): λ_max_/nm (ε/L mol^–1^cm^–1^): 226 (45707), 243 (52690), 255 (44281), 280 (73419), 285 (79280), 336 (13240), 424 (16426), 452 (19559). HRMS (ES+) found *m*/*z* 975.1472 [M – PF_6_]^+^, calculated *m*/*z* 975.1473 for [C_44_H_37_ClN_8_OF_6_PRu]^+^.

##### Synthesis of [Ru­(bipy)_2_(L^5^)]­(PF_6_)_3_

Ru­(bipy)_2_Cl_2_ (0.069 g, 0.14 mmol), **L** ^**5**^ (0.1 g, 0.14 mmol) and EtOH (15 mL) were heated for 16 h. An additional MeCN:Et_2_O reprecipitation was performed to yield the product as an orange solid (0.109 g, 54%). ^1^H NMR (400 MHz, (CD_3_)_2_CO, 298 K) δ_H_ (ppm): 8.74–8.63 (6H, m), 8.44 (1H, s), 8.22 (1H, dd, *J* = 5.2, 1.2 Hz), 8.19–8.13 (3H, m), 8.06 (2H, td, *J* = 7.9, 1.4 Hz), 8.01–7.96 (2H, m), 7.96–7.89 (5H, m), 7.86–7.80 (2H, m), 7.80–7.68 (13H, m), 7.68–7.62 (2H, m), 7.54 (2H, ddd, *J* = 7.6, 5.6, 1.3 Hz), 7.33 (2H, app. dddd, *J* = 7.6, 5.6, 3.6, 1.3 Hz), 7.24 (2H, dd, *J* = 8.5, 2.5 Hz), 5.05 (2H, d, *J* = 15.5 Hz). ^13^C­{^1^H} NMR (101 MHz, CD_3_OD) δ_C_ (ppm): 210.2, 158.8, 158.5, 152.9, 152.8, 139.3, 139.2, 137.9, 136.7, 135.5, 135.4, 132.67, 132.61, 131.9, 131.6, 131.4, 129.7, 129.0, 128.9, 127.7, 127.2, 125.61, 125.55, 124.7, 119.3, 118.4, 54.8, 30.7. ^31^P­{^1^H} NMR (162 MHz, CD_3_OD) δ_P_ (ppm): 22.91, −144.61 (sept). FTIR (solid, ATR) ν/cm^–1^: 3630, 3387, 2918, 2849, 2008, 1628, 1603, 1464, 1439, 1422, 1385, 1314, 1269, 1244, 1161, 1111, 1020, 997, 827, 760, 723, 689, 556, 430, 422, 415, 409, 403. UV–vis (CH_3_CN): λ_max_/nm (ε/L mol^–1^cm^–1^): 226 (69701), 242 (59782), 253 (56038), 278 (74681), 285 (84203), 327 (17735), 391 (18496), 421 (25979), 450 (21818). HRMS (ES+) found *m*/*z* 329.4169 [M – 3PF_6_]^3+^, calculated *m*/*z* 329.4161 for [C_58_H_45_N_7_OPRu]^3+^.

##### Synthesis of [Ru­(bipy)_2_(L^6^)]­(PF_6_)_3_

Ru­(bipy)_2_Cl_2_ (0.036 g, 0.07 mmol), **L** ^**6**^ (0.047 g, 0.07 mmol) and EtOH (10 mL) were heated for 16 h. Additional purification was performed through flash column chromatography using MeCN:H_2_O:HNO_3_ (7:1:sat.) as the eluent to yield the complex as an orange solid (0.053 g, 53%). ^1^H NMR (500 MHz, CD_3_CN, 298 K) δ_H_ (ppm): 8.83 (1H, br. s), 8.58 (1H, dd, *J* = 8.6, 1.2 Hz), 8.55–8.51 (3H, m), 8.49 (2H, app. dddd, *J* = 8.3, 2.3, 1.3, 0.8 Hz), 8.43 (1H, s), 8.11 (1H, q, *J* = 1.1 Hz), 8.10–8.07 (2H, m), 8.02–7.97 (3H, m), 7.92–7.89 (2H, m), 7.88 (1H, app. dt, *J* = 2.0, 1.4 Hz), 7.84–7.72 (15H, m), 7.69 (1H, dd, *J* = 8.3, 5.2 Hz), 7.53 (2H, app. dddd, *J* = 5.6, 3.8, 1.5, 0.8 Hz), 7.44 (2H, app. dddd, *J* = 7.7, 5.7, 3.6, 1.3 Hz), 7.22 (2H, app. dddd, *J* = 7.7, 5.7, 1.3, 0.8 Hz), 3.69–3.62 (2H, m), 3.07–3.01 (2H, m). ^13^C­{^1^H} NMR DEPT135 (126 MHz, CD_3_CN, 298 K) δ_C_ (ppm): 151.5, 150.7, 150.67, 150.62, 150.5, 136.6, 136.5, 135.1, 134.1, 132.58, 132.50, 130.6, 129.2, 129.1, 126.31, 126.29, 126.17, 126.13, 125.0, 124.3, 123.0, 122.9, 16.4, 16.0. ^31^P­{^1^H} NMR (162 MHz, CD_3_CN) δ_P_ (ppm): 24.39, −144.62 (sept). FTIR (solid, ATR) ν/cm^–1^: 3395, 3238, 2914, 2849, 2154, 1694, 1632, 1605, 1493, 1464, 1439, 1423, 1315, 1242, 1188, 1111, 997, 827, 760, 741, 723, 689, 660, 554, 523, 505, 482, 436, 419, 403. UV–vis (CH_3_CN): λ_max_/nm (ε/L mol^–1^cm^–1^): 224 (40005), 243 (33662), 257 (30232), 278 (54380), 284 (59888), 323 (12217), 419 (15183), 450 (14486). HRMS (ES+) found *m*/*z* 308.7448 [M – 3PF_6_]^3+^, calculated *m*/*z* 308.7441 for [C_53_H_4_N_7_OPRu]^3+^.

##### Synthesis of [Ru­(bipy)_2_(L^7^)]­(PF_6_)_3_

Ru­(bipy)_2_Cl_2_ (0.036 g, 0.074 mmol), **L** ^**7**^ (0.046 g, 0.074 mmol) and EtOH (10 mL) were heated for 16 h. Additional purification was performed through flash column chromatography using MeCN:H_2_O:HNO_3_ (7:1:sat.) as the eluent to yield the complex as an orange solid (0.014 g, 14%). ^1^H NMR (500 MHz, (CD_3_)_2_CO, 298 K) δ_H_ (ppm): 8.95 (1H, dd, *J* = 8.5, 1.2 Hz), 8.88–8.78 (5H, m), 8.39 (1H, dd, *J* = 5.2, 1.2 Hz), 8.34 (1H, dd, *J* = 8.4, 1.2 Hz), 8.24 (2H, tdd, *J* = 8.0, 3.2, 1.5 Hz), 8.19–8.10 (5H, m), 8.00–7.78 (18H, m), 7.65–7.60 (3H, m), 7.43 (2H, app. dddd, *J* = 10.1, 7.4, 5.7, 1.3 Hz), 7.29 (1H, s), 5.09 (2H, d, *J* = 14.3 Hz). ^13^C­{^1^H} NMR (126 MHz, (CD_3_)_2_CO, 298 K) δ_C_ (ppm): 152.6, 152.4, 152.29, 152.22, 147.9, 138.2, 138.12, 138.09, 137.98, 135.88, 135.86, 135.84, 135.82, 134.5, 134.4, 134.3, 134.2, 134.1, 131.9, 130.76, 130.69, 130.65, 130.59, 127.90, 127.86, 127.84, 127.76, 126.2, 125.2, 124.7, 124.60, 124.55, 124.51, 124.49, 31.4 (*C*H_2_, d, *J =* 56.70 Hz*)*. ^31^P­{^1^H} NMR (162 MHz, CD_3_CN) δ_P_ (ppm): 21.57, −144.63 (sept). FTIR (solid, ATR) ν/cm^–1^: 3387, 3092, 2916, 2849, 1701, 1630, 1603, 1541, 1466, 1439, 2961, 1423, 1314, 1260, 1161, 1105, 1026, 827, 760, 723, 689, 660, 648, 556, 511, 459, 438, 430, 411, 407, 401. UV–vis (CH_3_CN): λ_max_/nm (ε/L mol^–1^cm^–1^): 225 (42696), 244 (41851), 253 (40202), 278 (72272), 285 (82480), 349 (13776), 369 (15619), 424 (18748), 452 (20271). HRMS (ES+) found *m*/*z* 304.5586 [M-COCH_2_PPh_3_^+^ and 2PF_6_]^2+^, calculated *m*/*z* 304.5602 for [C_32_H_25_N_7_Ru]^2+^.

##### Synthesis of [Ru­(bipy)_2_(L^8^)]­(PF_6_)_3_

Ru­(bipy)_2_Cl_2_ (0.019 g, 0.04 mmol), **L** ^**8**^ (0.03 g, 0.04 mmol) and EtOH (8 mL) were heated for 16 h. An additional MeCN:Et_2_O reprecipitation was performed to yield the product as an orange solid (0.034 g, 59%). ^1^H NMR (500 MHz, (CD_3_)_2_CO, 298 K) δ_H_ (ppm): 8.94 (1H, dd, *J* = 8.5, 1.2 Hz), 8.84 (2H, app. ddt, *J* = 8.2, 2.1, 1.0 Hz), 8.80 (2H, app. ddt, *J* = 8.3, 3.5, 1.1 Hz), 8.62 (1H, dd, *J* = 8.3, 1.2 Hz), 8.39 (1H, dd, *J* = 5.2, 1.2 Hz), 8.27–8.20 (3H, m), 8.17–8.08 (4H, m), 8.02–7.92 (3H, m), 7.91–7.87 (3H, m), 7.86 (1H, ddd, *J* = 5.6, 1.5, 0.7 Hz), 7.85–7.77 (13H, m), 7.62 (2H, app. dddd, *J* = 7.8, 5.6, 2.3, 1.3 Hz), 7.44–7.35 (4H, m), 7.22 (2H, dd, *J* = 8.3, 2.5 Hz), 5.17 (2H, d, *J* = 15.1 Hz), 3.89 (4H, br. d, *J* = 108.0 Hz), 3.36 (4H, br. d, *J* = 5.1 Hz). ^13^C­{^1^H} NMR (126 MHz, (CD_3_)_2_CO, 298 K) δ_C_ (ppm): 153.1, 152.68, 152.66, 152.6, 152.5, 151.3, 138.68, 138.65, 138.56, 138.54, 136.5, 136.08, 136.05, 134.95, 134.88, 134.87, 134.4, 131.79, 131.75, 131.0, 130.9, 128.55, 128.52, 128.47, 128.43, 128.36, 128.34, 127.0, 126.6, 125.04, 125.01, 124.96, 124.93, 115.2, 53.6. ^31^P­{^1^H} NMR (162 MHz, CD_3_CN) δ_P_ (ppm): 22.67, −144.61 (sept). FTIR (solid, ATR) ν/cm^–1^: 2916, 2849, 1620, 1514, 1464, 1439, 1423, 1387, 1283, 1256, 1161, 1113, 1009, 829, 762, 729, 689, 556, 525, 517, 505, 494, 459, 446, 424, 417, 407. UV–vis (CH_3_CN): λ_max_/nm (ε/L mol^–1^cm^–1^): 231 (61356), 244 (54203), 256 (42129), 277 (58070), 284 (69660), 343 (11569), 426 (14668), 450 (17150). HRMS (ES+) found *m*/*z* 352.4364 [M – 3PF_6_], calculated *m*/*z* 352.4354 for [C_62_H_51_N_8_OPRu]^3+^.

##### Synthesis of [Ru­(bipy)_2_(L^9^)]­(PF_6_)_3_

Ru­(bipy)_2_Cl_2_ (0.060 g, 0.124 mmol), **L** ^**9**^ (0.042 g, 0.124 mmol), NaPF_6_ (0.052 g, 0.310 mmol) and EtOH (10 mL) where heated for 24 h following the general procedure. The product was obtained as an orange solid (0.067 g, 71%). ^1^H NMR (500 MHz, (CD_3_)_2_CO, 298 K) δ_H_ (ppm): 10.38 (1H, s), 8.96 (1H, d, *J* = 8.5 Hz), 8.91–8.72 (7H, app. m), 8.49 (1H, dd, *J* = 5.2, 1.2 Hz), 8.42 (1H, dd, *J* = 5.2, 1.2 Hz), 8.27 (3H, t, *J* = 7.9 Hz), 8.21–8.12 (4H, app. m), 7.97–7.87 (1H, app. m), 7.64 (2H, ddd, *J* = 7.2, 5.6, 1.3 Hz), 7.46–7.35 (2H, app. m), 4.74 (2H, s), 3.90 (6H, q, *J* = 7.2 Hz), 1.54 (9H, t, *J* = 7.2 Hz). ^13^C­{^1^H} NMR (126 MHz, (CD_3_)_2_CO, 298 K) δ_C_ (ppm): 164.6, 158.5, 158.5, 158.3, 158.2, 154.1, 153.4, 153.12, 153.05, 149.0, 147.1, 139.2, 139.08, 139.06, 137.8, 133.40, 133.36, 131.6, 128.91, 128.89, 128.80, 128.76, 127.2, 125.5, 125.43, 125.40, 122.9, 57.5, 8.3. FTIR (solid, ATR) ν/cm^–1^: 407, 419, 554, 662, 729, 760, 820, 829, 883, 899, 1011, 1101, 1125, 1164, 1192, 1209.37, 1240, 1256, 1304, 1331, 1389, 1425, 1447, 1458, 1468, 1508, 1560, 1605, 1719, 2882, 2918. UV–vis (CH_3_CN): λ_max_/nm (ε/L mol^–1^cm^–1^): 244 (43122), 256 (38281), 287 (73538), 379 (17756), 426 (14730), 458 (15989).

#### General Procedure for the Synthesis of Ir­(III) Complexes

[Ir­(tmq)_2_(NCMe)_2_]­PF_6_ (1 equiv) and chosen ligand (1 equiv) were combined in DCM or MeOH. The solution was stirred for 48 h. The solvent was concentrated under vacuum and a saturated solution of NH_4_PF_6_ was added to the solution. The product was extracted into DCM and washed with deionized water (3 × 10 mL). The DCM layer was dried over MgSO_4_ and filtered. The filtrate was taken, and the solvent removed under vacuum to yield the complex.

##### Synthesis of [Ir­(tmq)_2_(L^1^)]­PF_6_

Using [Ir­(tmq)_2_(NCMe)_2_]­PF_6_ (0.078 g, 0.084 mmol) and **L** ^**1**^ (0.030 g, 0.084 mmol). MeCN:Et_2_O reprecipitation was performed to yield the product as an orange solid (0.053 g, 53%). ^1^H NMR (500 MHz, (CD_3_)_2_CO, 298 K) δ_H_ (ppm): 10.01 (1H, br. s), 8.99 (1H, app. s), 8.98 (1H, q, *J* = 1.3 Hz), 8.91 (1H, dd, *J* = 5.2, 1.4 Hz), 8.73 (1H, dd, *J* = 8.4, 1.4 Hz), 8.54 (2H, app. ddd, *J* = 8.2, 3.1, 1.2 Hz), 8.39 (1H, s), 8.24–8.17 (1H, m), 8.16 (1H, dd, *J* = 8.2, 5.2 Hz), 8.07 (1H, app. d, *J* = 1.8 Hz), 8.06 (1H, app. d, *J* = 1.9 Hz), 7.65–7.63 (m, 1H), 7.65–7.60 (m, 1H), 7.54 (d, *J* = 1.1 Hz, 1H), 7.52 (d, *J* = 1.1 Hz, 1H), 7.29 (2H, app. dddd, *J* = 8.4, 7.1, 1.4, 0.7 Hz), 7.22 (1H, d, *J* = 1.1 Hz), 7.14 (1H, d, *J* = 1.0 Hz), 6.88 (2H, tt, *J* = 7.2, 1.4 Hz), 6.84–6.77 (2H, m), 4.81 (2H, s), 3.36 (6H, s), 2.17 (6H, dd, *J* = 6.7, 1.0 Hz), 1.73–1.67 (6H, m). ^13^C­{^1^H} NMR (126 MHz, (CD_3_)_2_CO, 298 K) δ_C_ (ppm): 149.7, 148.9, 139.4, 136.2, 136.1, 136.0, 131.27, 131.24, 130.78, 130.75, 129.6, 128.9, 128.73, 128.68, 127.8, 127.0, 123.9, 123.8, 123.7, 123.6, 122.0, 27.3, 19.52, 19.48, 19.18, 19.16. FTIR (solid, ATR) ν/cm^–1^: 2980, 1682, 1628, 1578, 1524, 1506, 1479, 1454, 1429, 1383, 1344, 1321, 1269, 1217, 1165, 1136, 1061, 993, 841, 762, 731, 702, 629, 557, 474, 419, 411, 401. UV–vis (CH_3_CN): λ_max_/nm (ε/L mol^–1^cm^–1^): 222 (94091), 258 (82587), 288 (56555), 330 (29093), 357 (33363), 383 (39231), 461 (11436). HRMS (ES^+^) found *m*/*z* 1034.2928 [M – PF_6_]^+^, calculated *m*/*z* 1034.2925 for [C_54_H_44_N_7_OClIr]^+^.

##### Synthesis of [Ir­(tmq)_2_(L^2^)]­PF_6_

Using [Ir­(tmq)_2_(NCMe)_2_]­PF_6_ (0.082 g, 0.09 mmol) and **L** ^**2**^ (0.026 g, 0.09 mmol). MeCN:Et_2_O reprecipitation was performed to yield the product as an orange solid (0.029 g, 29%). ^1^H NMR (500 MHz, (CD_3_)_2_CO, 298 K) δ_H_ (ppm): 9.68 (1H, s), 8.99–8.94 (2H, m), 8.86 (1H, dt, *J* = 5.1, 1.2 Hz), 8.71 (1H, dd, *J* = 8.3, 1.4 Hz), 8.56–8.52 (3H, m), 8.44 (1H, s), 8.21 (1H, ddd, *J* = 8.5, 5.2, 2.1 Hz), 8.14 (1H, dd, *J* = 8.3, 5.1 Hz), 7.53–7.51 (2H, m), 7.30–7.26 (2H, m), 7.19 (1H, d, *J* = 3.1 Hz), 7.13–7.12 (1H, d, *J* = 2.2 Hz), 6.90–6.84 (3H, m), 6.79 (3H, app. ddd, *J* = 10.3, 7.6, 1.4 Hz), 3.92 (2H, t, *J* = 6.3 Hz), 3.37–3.34 (6H, m), 3.06 (2H, t, *J* = 6.3 Hz), 2.18–2.14 (6H, m), 1.73–1.67 (6H, m). ^13^C­{^1^H} NMR (126 MHz, (CD_3_)_2_CO, 298 K) δ_C_ (ppm): 149.7, 148.6, 139.30, 139.27, 136.0, 135.9, 135.2, 131.3, 131.2, 130.8, 130.7, 128.7, 127.8, 127.1, 127.0, 123.9, 123.8, 123.6, 40.6 (*C*H_2_), 39.9 (*C*H_2_), 27.4, 19.5, 19.2. FTIR (solid, ATR) ν/cm^–1^: 1699, 1628, 1580, 1526, 1481, 1454, 1427, 1404, 1344, 1321, 1267, 1217, 1165, 1061, 1026, 993, 841, 762, 731, 702, 629, 557, 409. UV–vis (CH_3_CN): λ_max_/nm (ε/L mol^–1^cm^–1^): 222 (1000859), 280 (66266), 255 (71966), 279 (56662), 333 (24846), 349 (25481), 370 (32600), 382 (32416), 460 (9027). HRMS (ES+) found *m*/*z* 972.2781 [M – PF_6_]^+^, calculated *m*/*z* 972.2769 for [C_49_H_42_N_7_OClIr]^+^.

##### Synthesis of [Ir­(tmq)_2_(L^3^)]­PF_6_

Using [Ir­(tmq)_2_(NCMe)_2_]­PF_6_ (0.063 g, 0.07 mmol) and **L** ^**3**^ (0.019 g, 0.07 mmol). The product was obtained as a red solid (0.054 g, 71%). ^1^H NMR (500 MHz, (CD_3_)_2_CO, 298 K) δ_H_ (ppm): 9.87 (1h, s), 8.99 (1H, d, *J* = 6.3 Hz), 8.90 (2H, app. q, *J* = 7.5 Hz), 8.73 (1H, d, *J* = 7.1 Hz), 8.54 (2H, dd, *J* = 8.8, 4.8 Hz), 8.37 (1H, d, *J* = 4.7 Hz), 8.22 (1H, d, *J* = 6.6 Hz), 8.16 (1H, d, *J* = 6.7 Hz), 7.52 (2H, d, *J* = 7.4 Hz), 7.28 (2H, app. q, *J* = 7.2 Hz), 7.18 (1H, d, *J* = 4.8 Hz), 7.10 (1H, d, *J* = 4.9 Hz), 6.87 (2H, app. t, *J* = 7.2 Hz), 6.79 (2H, app. q, *J* = 7.9 Hz), 4.42 (2H, app. t, *J* = 4.0 Hz), 3.40–3.31 (6H, m), 2.16 (6H, d, *J* = 7.8 Hz), 1.69 (6H, app. dt, *J* = 18.0, 4.3 Hz). ^13^C­{^1^H} NMR (126 MHz, (CD_3_)_2_CO, 298 K) δ_C_ (ppm): 149.8, 149.0, 139.4, 136.1, 136.0, 135.3, 131.27, 131.24, 130.8, 128.7, 127.8, 127.2, 123.9, 123.74, 123.66, 121.2, 43.6, 27.3, 19.5, 19.2. FTIR (solid, ATR) ν/cm^–1^: 3636, 3375, 3051, 2928, 1697, 1628, 1578, 1526, 1483, 1452, 1425, 1373, 1342, 1321, 1267, 1217, 1167, 1136, 1061, 993, 837, 795, 762, 700, 658, 627, 556, 476, 769, 451, 440, 432, 424, 417. UV–vis (CH_3_CN): λ_max_/nm (ε/L mol^–1^cm^–1^): 217 (100859), 254 (78091), 261 (82382), 287 (61892), 322 (28765), 373 (40553), 382 (39923), 459 (10575). HRMS (ES+) found *m*/*z* 958.2600 [M – PF_6_]^+^, calculated *m*/*z* 958.2612 for [C_48_H_40_N_7_OClIr]^+^.

##### Synthesis of [Ir­(tmq)_2_(L^4^)]­PF_6_

Using [Ir­(tmquin)_2_(NCMe)_2_]­PF_6_ (0.1 g, 0.1 mmol) and **L** ^**4**^ (0.046 g, 0.1 mmol) and a mixed solvent of DCM (10 mL) and MeOH (5 mL). The product was obtained as a red solid (0.064 g, 47%). ^1^H NMR (500 MHz, CD_3_CN, 298 K) δ_H_ (ppm): 8.83 (1H, d, *J* = 8.4 Hz), 8.75 (1H, d, *J* = 5.0 Hz), 8.59 (1H, d, *J* = 5.0 Hz), 8.55–8.43 (3H, m), 8.08–8.00 (1H, m), 7.94–7.87 (1H, m), 7.49 (6H, d, *J* = 12.2 Hz), 7.27 (2H, s), 7.15 (1H, s), 7.03 (1H, s), 6.85 (2H, d, *J* = 7.8 Hz), 6.70 (2H, app. t, *J* = 8.9 Hz), 4.67 (2H, s), 4.00 (2H, br. s), 3.70 (2H, br. s), 3.35 (6H, d, *J* = 4.1 Hz), 3.08 (41h, br. s), 2.17 (6H, d, *J* = 4.5 Hz), 1.67 (6H, d, *J* = 7.8 Hz). ^13^C­{^1^H} NMR (126 MHz, (CD_3_)_2_CO, 298 K) δ_C_ (ppm): 149.5, 147.6, 138.5, 136.4, 136.2, 136.0, 131.6, 130.92, 130.89, 129.6, 128.3, 128.2, 127.6, 127.1, 124.2, 123.91, 123.88, 114.9, 46.4, 27.4, 19.7, 19.4. FTIR (solid, ATR) ν/cm^–1^: 3644, 3406, 3053, 2160, 1614, 1578, 1524, 1483, 1447, 1429, 1371, 1344, 1317, 1281, 1256, 1217, 1161, 1132, 1061, 1009, 993, 835, 762, 700, 629, 556, 449, 442, 428, 415, 409, 403. UV–vis (CH_3_CN): λ_max_/nm (ε/L mol^–1^cm^–1^): 218 (87376), 256 (67088), 283 (46719), 324 (21157), 352 (25132), 370 (31035), 384 (29248), 461 (7786). HRMS (ES+) found *m*/*z* 1103.3500 [M – PF_6_]^+^, calculated *m*/*z* 1103.3504 for [C_58_H_51_N_8_OClIr]^+^.

##### Synthesis of [It­(tmq)_2_(L^5^)]­(PF_6_)_2_

Using [Ir­(tmq)_2_(NCMe)_2_]­PF_6_ (0.05 g, 0.06 mmol) and **L** ^**5**^ (0.039 g, 0.06 mmol) in EtOH (8 mL) and heated for 16 h. An additional MeCN:Et_2_O reprecipitation was performed to yield the product as an orange solid (0.056 g, 65%). ^1^H NMR (500 MHz, CDCl_3_, 298 K) δ_H_ (ppm): 10.01 (1s, br. s), 8.88 (1H, s), 8.53 (2H, dd, *J* = 18.2, 4.6 Hz), 8.42 (3H, dd, *J* = 15.9, 7.9 Hz), 8.33 (1H, s), 7.96 (1H, s), 7.79 (6 H, dd, *J* = 17.7, 9.2 Hz), 7.59 (14H, dd, *J* = 21.6, 10.9 Hz), 7.27 (4H, d, *J* = 19.4 Hz), 7.02 (3H, d, *J* = 13.1 Hz), 6.91–6.84 (3H, m), 6.63 (2H, dd, *J* = 16.2, 7.7 Hz), 4.76 (2H, d, *J* = 14.7 Hz), 3.39 (6H, d, *J* = 14.0 Hz), 2.17 (6H, t, *J* = 6.5 Hz), 1.67 (6H, s). ^13^C­{^1^H} NMR (126 MHz, (CD_3_)_2_CO, 298 K) δ_C_ (ppm): 149.7, 148.9, 139.4, 136.2, 136.1, 136.0, 135.9, 135.0, 134.8, 132.04, 131.96, 131.3, 131.2, 131.1, 130.89, 130.76, 129.09, 129.05, 128.69, 128.64, 127.8, 127.0, 123.9, 123.7, 123.6, 122.3, 29.6 (*C*H_2_, d, *J =* 48.11 *Hz)*, 27.3, 19.51, 19.46, 19.17, 19.15. ^31^P­{^1^H} NMR (162 MHz, CD_3_CN) δ_P_ (ppm): 22.24, −144.31 (sept). FTIR (solid, ATR) ν/cm^–1^: 3653, 3387, 3049, 2920, 1674, 1626, 1578, 1524, 1479, 1437, 1344, 1319, 1269, 1217, 1163, 1134, 1111, 1059, 993, 831, 739, 721, 689, 627, 556, 538, 494, 475, 449, 440, 420, 409. UV–vis (CH_3_CN): λ_max_/nm (ε/L mol^–1^cm^–1^): 214 (148487), 258 (102786), 282 (7433), 326 (33019), 349 (37181), 381 (46937), 386 (44741), 459 (12659). HRMS (ES+) found *m*/*z* 1260.4084 [M – PF_6_]^+^, calculated *m*/*z* 1260.4076 for [C_72_H_59_N_7_OPIr]^+^.

##### Synthesis of [It­(tmq)_2_(L^6^)]­(PF_6_)_2_

Using [Ir­(tmq)_2_(NCMe)_2_]­PF_6_ (0.082 g, 0.09 mmol) and **L** ^**6**^ (0.057 g, 0.09 mmol) in DCM (10 mL). An additional MeCN:Et_2_O reprecipitation was performed to yield the product as a red solid (0.088 g, 88%). ^1^H NMR (500 MHz, CD_3_CN, 298 K) δ_H_ (ppm): 8.71 (1H, dd, *J* = 5.2, 1.3 Hz), 8.63–8.56 (2H, m), 8.53–8.44 (3H, m), 8.42 (1H, dd, *J* = 8.4, 1.3 Hz), 8.09 (1H, s), 7.92 (1H, dd, *J* = 8.6, 5.1 Hz), 7.89–7.80 (4H, m), 7.78–7.66 (12H. m), 7.49 (2H, dd, *J* = 2.7, 1.1 Hz), 7.28 (2H, app. dddd, *J* = 8.4, 7.2, 3.6, 1.3 Hz), 7.03 (1H, s), 6.94 (1H, s), 6.90–6.83 (2H, m), 6.75 (2H, ddd, *J* = 10.8, 7.7, 1.2 Hz), 3.60–3.51 (2H, m), 3.30 (6H, d, *J* = 3.7 Hz), 2.92 (2H, dt, *J* = 12.9, 7.7 Hz), 2.14 (6H, dd, *J* = 5.6, 0.8 Hz), 1.62 (6H, d, *J* = 18.9 Hz). ^13^C­{^1^H} NMR (126 MHz, CD_3_CN, 298 K) δ_C_ (ppm): 149.8, 148.8, 139.3, 136.17, 136.12, 136.10, 136.05, 134.87, 134.63, 134.55, 131.58, 131.55, 131.2, 131.1, 130.87, 130.85, 128.7, 127.8, 127.1, 123.97, 123.91, 123.86, 120.1, 29.5, 27.6, 19.6, 19.4, 18.4, 17.97. ^31^P­{^1^H} NMR (162 MHz, CD_3_CN) δ_P_ (ppm): 24.30, −144.63 (sept). FTIR (solid, ATR) ν/cm^–1^: 1699, 1630, 1580, 1526, 1483, 1439, 1344, 1321, 1265, 1238, 1217, 1165, 1113, 1061, 1026, 995, 835, 760, 725, 690, 629, 557, 525, 505, 484. UV–vis (CH_3_CN): λ_max_/nm (ε/L mol^–1^cm^–1^): 215 (101890), 259 (68038), 262 (6948), 274 (60893), 289 (48318), 323 (24209), 382 (34150), 386 (32911), 460 (8940). HRMS (ES+) found *m*/*z* 630.7136 [M - 2PF_6_]^2+^, calculated *m*/*z* 630.7071 for [C_72_H_59_N_7_OPIr]^2+^.

##### Synthesis of [It­(tmq)_2_(L^7^)]­(PF_6_)_2_

Using [Ir­(tmq)_2_(NCMe)_2_]­PF_6_ (0.083 g, 0.09 mmol), **L** ^**7**^ (0.057 g, 0.09 mmol) in DCM (10 mL). An additional MeCN:Et_2_O reprecipitation was performed to yield the product as a red solid (0.094 g, 94%). ^1^H NMR (500 MHz, CD_3_CN, 298 K) δ_H_ (ppm): 9.15 (1H, s), 8.70 (1H, dd, *J* = 5.1, 1.3 Hz), 8.64 (1H, dd, *J* = 5.1, 1.4 Hz), 8.53–8.48 (2H, m), 8.43 (1H, dd, *J* = 8.4, 1.3 Hz), 8.23 (1H, dd, *J* = 8.6, 1.3 Hz), 7.95 (1H, s), 7.90–7.82 (5H, m), 7.79–7.72 (6H, m), 7.71–7.66 (6H, m), 7.48 (1H, d, *J* = 1.2 Hz), 7.45 (1H, d, *J* = 1.1 Hz), 7.28 (2H, app. dtd, *J* = 8.5, 7.4, 1.3 Hz), 7.04 (1H, s), 6.89–6.83 (3H, m), 6.78–6.73 (2H, m), 4.75 (2H, d, *J* = 14.1 Hz), 3.30 (6H, d, *J* = 15.6 Hz), 2.11 (6H, dd, *J* = 15.9, 1.0 Hz), 1.59 (6H, d, *J* = 31.9 Hz). ^13^C­{^1^H} NMR (126 MHz, CD_3_OD, 298 K) δ_C_ (ppm): 149.9, 149.3, 139.8, 136.2, 136.1, 134.9, 134.8, 131.7, 131.2, 131.1, 128.5, 128.4, 127.9, 127.4, 124.1, 123.8, 54.5, 30.4, 27.1, 27.0, 18.2. ^31^P­{^1^H} NMR (162 MHz, CD_3_CD) δ_P_ (ppm): 21.42, −144.56 (sept). FTIR (solid, ATR) ν/cm^–1^: 3622, 3374, 3057, 2922, 1697, 1630, 1578, 1524, 1483, 1439, 1342, 1319, 1267, 1215, 1162, 1134, 1111, 993, 831, 739, 729, 687, 627, 556, 507, 476, 732, 419, 411, 403. UV–vis (CH_3_CN): λ_max_/nm (ε/L mol^–1^cm^–1^): 222 (134632), 258 (91770), 268 (93098), 272 (85171), 281 (73414), 328 (32875), 279 (48700), 387 (44347), 456 (12925). HRMS (ES+) found *m*/*z* 592.6931 [M - 2PF_6_]^2+^, calculated *m*/*z* 592.6912 for [C_66_H_55_N_7_OPIr]^2+^.

##### Synthesis of [It­(tmq)_2_(L^8^)]­(PF_6_)_2_

Using [Ir­(tmq)_2_(NCMe)_2_]­PF_6_ (0.036 g, 0.04 mmol), **L** ^**8**^ (0.03 g, 0.4 mmol) and DCM (5 mL). An additional MeCN:Et_2_O reprecipitation was performed to yield the product as a red solid (0.020 g, 32%). ^1^H NMR (500 MHz, CD_3_CN) δ 8.70–8.65 (2H, m), 8.54–8.47 (3H, m), 8.33 (1H, dd, *J* = 8.3, 1.3 Hz), 7.93–7.89 (2H, m), 7.89–7.84 (3H, m), 7.80 (1H, dd, *J* = 8.3, 5.2 Hz), 7.71–7.66 (6H, m), 7.59 (6H, app. dddd, *J* = 12.7, 6.6, 2.0, 1.2 Hz), 7.48 (2H, dd, *J* = 5.6, 1.1 Hz), 7.36 (1H, s), 7.30–7.23 (4H, m), 7.08 (1H, s), 7.00 (2H, dd, *J* = 8.3, 2.6 H), 6.96 (1H, s), 6.88–6.83 (2H, m), 6.79–6.71 (2H, m), 4.67 (2H, d, *J* = 14.9 Hz), 3.51 (2H, d, *J* = 77.5 Hz), 3.30 (6H, d, *J* = 8.8 Hz), 3.13–2.86 (4H, m), 2.14 (6H, dd, *J* = 7.8, 0.9 Hz), 1.63 (6H, d, *J* = 15.8 Hz). ^13^C­{^1^H} NMR (126 MHz, CD_3_CN, 298 K) δ_C_ (ppm): δ 149.5, 147.6, 138.5, 136.4, 136.25, 136.22, 136.15, 136.0, 135.1, 135.0, 131.84, 131.80, 131.5, 131.1, 131.0, 130.82, 130.79, 128.67, 128.65, 128.52, 128.49, 127.6, 127.1, 124.2, 123.91, 123.85, 123.82, 114.9, 53.3 (*C*H_2_), 30.5 (*C*H_2_), 30.3 (*C*H_2_, d, *J =* 48.81 *Hz*), 27.7, 19.7, 19.4. ^31^P­{^1^H} NMR (162 MHz, CD_3_CN) δ_P_ (ppm): 22.66, −144.60 (sept). FTIR (solid, ATR) ν/cm^–1^: 3410, 3048, 3636, 2916, 2849, 1616, 1580, 1558, 1522, 1485, 1439, 4373, 1344, 1319, 1256, 1217, 1165, 1134, 1113, 1059, 1009, 995, 835, 689, 629, 556, 527, 505, 446, 415, 409. UV–vis (CH_3_CN): λ_max_/nm (ε/L mol^–1^cm^–1^): 214 (125126), 251 (808095), 258 (81380), 279 (56922), 282 (54207), 321 (23219), 349 (232219), 349 (27825), 378 (35788), 382 (34275), 461 (8396). HRMS (ES+) found *m*/*z* 665.2383 [M - 2PF_6_]^2+^, calculated *m*/*z* 665.2367 for [C_76_H_66_N_8_OPIr]^2+^.

##### Synthesis of [It­(tmq)_2_(L^9^)]­(PF_6_)_2_

Using [Ir­(tmq)_2_(NCMe)_2_]­PF_6_ (0.029 g, 0.032 mmol), **L** ^**9**^ (0.013 g, 0.032 mmol) and DCM (5 mL). An additional MeCN:Et_2_O reprecipitation was performed to yield the product as a red solid (0.030 g, 60%). ^1^H NMR (500 MHz, (CD_3_)_2_CO, 298 K) δ_H_ (ppm): 10.20 (1H, s), 9.04 (1H, d, *J* = 5.5 Hz), 9.01–8.90 (2H, m), 8.76 (1H, d, *J* = 8.1 Hz), 8.55 (2H, d, *J* = 8.3 Hz), 8.45 (1H, s), 8.22 (2H, ddd, *J* = 11.1, 8.4, 5.1 Hz), 7.56 (2H, d, *J* = 8.2 Hz), 7.30 (2H, t, *J* = 7.7 Hz), 7.20 (1H, s), 7.12 (1H, s), 6.85 (4H, dt, *J* = 25.4, 7.2 Hz), 4.62 (2H, s), 3.83 (6H, q, *J* = 7.3 Hz), 3.37 (6H, s), 2.18 (6H, d, *J* = 13.5 Hz), 1.71 (6H, d, *J* = 13.5 Hz)­1.48 (9H, t, *J* = 7.2 Hz). ^13^C­{^1^H} NMR (126 MHz, (CD_3_)_2_CO, 298 K) δ_C_ (ppm): 164.3, 164.1, 164.0, 152.94, 152.88, 150.3, 149.6, 146.1, 142.0, 141.63, 141.56, 140.0, 139.9, 139.8, 136.4, 136.3, 131.61, 131.57, 131.13, 131.10, 129.08, 129.03, 128.3, 127.7, 124.1, 122.6, 55.8, 19.80, 19.78, 19.5, 8.1. FTIR (solid, ATR) ν/cm^–1^: 407, 422, 473, 532, 556, 627, 700, 729, 762, 837, 881, 993, 1117, 1163, 1213, 1240, 1258, 1319, 1346, 1395, 1427, 1481, 1501, 1558, 1578, 1605, 1717, 1773, 2913, 2978, 3048, 3647. UV–vis (CH_3_CN): λ_max_/nm (ε/L mol^–1^cm^–1^): 216 (79999), 165 (67735), 322 (23407), 374 (32874). HRMS (ES+) found *m*/*z* 512.2075 [M - 2PF_6_]^2+^, calculated *m*/*z* 512.2059 for [C_54_H_55_N_8_OIr]^2+^.

## Supplementary Material



## Data Availability

Information on the data underpinning this publication, including access details, can be found in the Cardiff University Research Data Repository at 10.17035/cardiff.30998695.

## Notes

NMR and HRMS spectra for all compounds; cyclic voltammetry data for complexes; data collection parameters for the X-ray crystal structures; additional TD-DFT tabulated data (PDF)
