# Supplementary material for: Synthesis, Photophysical and TD-DFT Evaluation of Triphenylphosphonium-Labeled Ru(II) and Ir(III) Luminophores
Source: Inorg Chem. 2026 Jan 8;65(2):1336–54. doi: 10.1021/acs.inorgchem.5c04913 (PMC12820962; doi:10.1021/acs.inorgchem.5c04913)
Supplement: Supplementary file 1 [file ic5c04913_si_001.pdf]

Supporting Information

Synthesis, photophysical and TD-DFT evaluation of triphenylphosphonium-labelled Ru(II) and Ir(III) luminophores

Alexandra R. Ibbott,[a] Steffan Walker-Griffiths,[b] Peter N. Horton,[c] Joseph M. Beames,[b] Catherine L. Andrews,[a] Simon J. Coles,[b] and Simon J. A. Pope[a]*

[a]School of Chemistry, Main Building, Cardiff University, Cardiff CF10 3AT, Cymru/Wales; [b] School of Chemistry, The University of Birmingham, Edgbaston, Birmingham, B15 2TT, UK; [c] UK National Crystallographic Service, Chemistry, Faculty of Natural and Environmental Sciences, University of Southampton, Highfield, Southampton, SO17 1BJ, England, UK

Email: popesj@cardiff.ac.uk

		Page
Figure S1	^{1}H NMR spectrum of $\mathbf{L^1}$	S5
Figure S2	$^{13}C\{^1H\}$ NMR spectrum of $\mathbf{L^1}$	S5
Figure S3	^{1}H NMR spectrum of $\mathbf{L^2}$	S6
Figure S4	$^{13}C\{^1H\}$ NMR spectrum of $\mathbf{L^2}$	S6
Figure S5	^{1}H NMR spectrum of $\mathbf{L^3}$	S7
Figure S6	$^{13}C\{^1H\}$ NMR spectrum of $\mathbf{L^3}$	S7
Figure S7	^{1}H NMR spectrum of $\mathbf{L^4}$	S8
Figure S8	$^{13}C\{^1H\}$ NMR spectrum of $\mathbf{L^4}$	S8
Figure S9	^{1}H NMR spectrum of $\mathbf{L^5}$	S9
Figure S10	$^{13}C\{^1H\}$ NMR spectrum of $\mathbf{L^5}$	S9
Figure S11	$^{31}P\{^1H\}$ NMR spectrum of $\mathbf{L^5}$	S10
Figure S12	^{1}H NMR spectrum of $\mathbf{L^6}$	S10
Figure S13	$^{13}C\{^1H\}$ NMR spectrum of $\mathbf{L^6}$	S11
Figure S14	$^{31}P\{^1H\}$ NMR spectrum of $\mathbf{L^6}$	S11
Figure S15	^{1}H NMR spectrum of $\mathbf{L^7}$	S12
Figure S16	$^{13}C\{^1H\}$ NMR spectrum of $\mathbf{L^7}$	S12
Figure S17	$^{31}P\{^1H\}$ NMR spectrum of $\mathbf{L^7}$	S13
Figure S18	^{1}H NMR spectrum of $\mathbf{L^8}$	S13
Figure S19	$^{13}C\{^1H\}$ NMR spectrum of $\mathbf{L^8}$	S14
Figure S20	$^{31}P\{^1H\}$ NMR spectrum of $\mathbf{L^8}$	S14
Figure S21	^{1}H NMR spectrum of $[Ru(bipy)_2(\mathbf{L^1})](PF_6)_2$	S15
Figure S22	$^{13}C\{^1H\}$ NMR spectrum of $[Ru(bipy)_2(\mathbf{L^1})](PF_6)_2$	S15
Figure S23	^{1}H NMR spectrum of $[Ru(bipy)_2(\mathbf{L^2})](PF_6)_2$	S16
Figure S24	$^{13}C\{^1H\}$ NMR spectrum of $[Ru(bipy)_2(\mathbf{L^2})](PF_6)_2$	S16
Figure S25	^{1}H NMR spectrum of $[Ru(bipy)_2(\mathbf{L^3})](PF_6)_2$	S17
Figure S26	$^{13}C\{^1H\}$ NMR spectrum of $[Ru(bipy)_2(\mathbf{L^3})](PF_6)_2$	S17
Figure S27	^{1}H NMR spectrum of $[Ru(bipy)_2(\mathbf{L^4})](PF_6)_2$	S18
Figure S28	$^{13}C\{^1H\}$ NMR spectrum of $[Ru(bipy)_2(\mathbf{L^4})](PF_6)_2$	S18
Figure S29	^{1}H NMR spectrum of $[Ru(bipy)_2(\mathbf{L^5})](PF_6)_3$	S19
Figure S30	$^{13}C\{^1H\}$ NMR spectrum of $[Ru(bipy)_2(\mathbf{L^5})](PF_6)_3$	S19
Figure S31	$^{31}P\{^1H\}$ NMR spectrum of $[Ru(bipy)_2(\mathbf{L^5})](PF_6)_3$	S20
Figure S32	^{1}H NMR spectrum of $[Ru(bipy)_2(\mathbf{L^6})](PF_6)_3$	S20
Figure S33	$^{13}C\{^1H\}$ NMR spectrum of $[Ru(bipy)_2(\mathbf{L^6})](PF_6)_3$	S21
Figure S34	$^{31}P\{^1H\}$ NMR spectrum of $[Ru(bipy)_2(\mathbf{L^6})](PF_6)_3$	S21
Figure S35	^{1}H NMR spectrum of $[Ru(bipy)_2(\mathbf{L^7})](PF_6)_3$	S22

Figure S36	$^{13}C\{^1H\}$ NMR spectrum of [Ru(bipy)$_2$(**L**^{7})](PF$_6$)$_3$	S22
Figure S37	$^{31}P\{^1H\}$ NMR spectrum of [Ru(bipy)$_2$(**L**^{7})](PF$_6$)$_3$	S23
Figure S38	^1H NMR spectrum of [Ru(bipy)$_2$(**L**^{8})](PF$_6$)$_3$	S23
Figure S39	$^{13}C\{^1H\}$ NMR spectrum of [Ru(bipy)$_2$(**L**^{8})](PF$_6$)$_3$	S24
Figure S40	$^{31}P\{^1H\}$ NMR spectrum of [Ru(bipy)$_2$(**L**^{8})](PF$_6$)$_3$	S24
Figure S41	^1H NMR spectrum of [Ir(tmq)$_2$(**L**^{1})](PF$_6$)	S25
Figure S42	$^{13}C\{^1H\}$ NMR spectrum of [Ir(tmq)$_2$(**L**^{1})](PF$_6$)	S25
Figure S43	^1H NMR spectrum of [Ir(tmq)$_2$(**L**^{2})](PF$_6$)	S26
Figure S44	$^{13}C\{^1H\}$ NMR spectrum of [Ir(tmq)$_2$(**L**^{2})](PF$_6$)	S26
Figure S45	^1H NMR spectrum of [Ir(tmq)$_2$(**L**^{3})](PF$_6$)	S27
Figure S46	$^{13}C\{^1H\}$ NMR spectrum of [Ir(tmq)$_2$(**L**^{3})](PF$_6$)	S27
Figure S47	^1H NMR spectrum of [Ir(tmq)$_2$(**L**^{4})](PF$_6$)	S28
Figure S48	$^{13}C\{^1H\}$ NMR spectrum of [Ir(tmq)$_2$(**L**^{4})](PF$_6$)	S28
Figure S49	^1H NMR spectrum of [Ir(tmq)$_2$(**L**^{5})](PF$_6$)$_2$	S29
Figure S50	$^{13}C\{^1H\}$ NMR spectrum of [Ir(tmq)$_2$(**L**^{5})](PF$_6$)$_2$	S29
Figure S51	$^{31}P\{^1H\}$ NMR spectrum of [Ir(tmq)$_2$(**L**^{5})](PF$_6$)$_2$	S30
Figure S52	^1H NMR spectrum of [Ir(tmq)$_2$(**L**^{6})](PF$_6$)$_2$	S30
Figure S53	$^{13}C\{^1H\}$ NMR spectrum of [Ir(tmq)$_2$(**L**^{6})](PF$_6$)$_2$	S31
Figure S54	$^{31}P\{^1H\}$ NMR spectrum of [Ir(tmq)$_2$(**L**^{6})](PF$_6$)$_2$	S31
Figure S55	^1H NMR spectrum of [Ir(tmq)$_2$(**L**^{7})](PF$_6$)$_2$	S32
Figure S56	$^{13}C\{^1H\}$ NMR spectrum of [Ir(tmq)$_2$(**L**^{7})](PF$_6$)$_2$	S32
Figure S57	$^{31}P\{^1H\}$ NMR spectrum of [Ir(tmq)$_2$(**L**^{7})](PF$_6$)$_2$	S33
Figure S58	^1H NMR spectrum of [Ir(tmq)$_2$(**L**^{8})](PF$_6$)$_2$	S33
Figure S59	$^{13}C\{^1H\}$ NMR spectrum of [Ir(tmq)$_2$(**L**^{8})](PF$_6$)$_2$	S34
Figure S60	$^{31}P\{^1H\}$ NMR spectrum of [Ir(tmq)$_2$(**L**^{8})](PF$_6$)$_2$	S34
Figure S61	^1H NMR spectrum of [Ru(bipy)$_2$(**L**^{9})](PF$_6$)$_3$	S35
Figure S62	$^{13}C\{^1H\}$ NMR spectrum of [Ru(bipy)$_2$(**L**^{9})](PF$_6$)$_3$	S35
Figure S63	^1H NMR spectrum of [Ir(tmq)$_2$(**L**^{9})](PF$_6$)$_2$	S36
Figure S64	$^{13}C\{^1H\}$ NMR spectrum of [Ir(tmq)$_2$(**L**^{9})](PF$_6$)$_2$	S36
Figure S65	HRMS for [Ru(bipy)$_2$(**L**$^{1-4}$)](PF$_6$)$_2$ complexes	S37
Figure S66	HRMS for [Ru(bipy)$_2$(**L**$^{5-8}$)](PF$_6$)$_3$ complexes	S37
Figure S67	HRMS for [Ir(tmq)$_2$(**L**$^{1-4}$)](PF$_6$) complexes	S38
Figure S68	HRMS for [Ir(tmq)$_2$(**L**$^{5-8}$)](PF$_6$)$_2$ complexes	S38
Figure S69	CVs of [Ru(bipy)$_2$(**L**)](PF$_6$)$_n$ and [Ir(tmq)$_2$(**L**)](PF$_6$)$_n$	S39
Figure S70	Calculated Kohn-Sham molecular orbitals for [Ru(bipy)$_2$(**L**^{6})]$^{2+}$	S40
Figure S71	Calculated Kohn-Sham molecular orbitals for [Ru(bipy)$_2$(**L**^{7})]$^{2+}$	S40

Figure S72	Calculated Kohn-Sham molecular orbitals for $[Ru(bipy)_2(\mathbf{L^8})]^{2+}$	S41
Figure S73	Calculated Kohn-Sham molecular orbitals for $[Ir(tmq)_2(\mathbf{L^6})]^{2+}$	S41
Figure S74	Calculated Kohn-Sham molecular orbitals for $[Ir(tmq)_2(\mathbf{L^7})]^{2+}$	S42
Figure S75	Calculated Kohn-Sham molecular orbitals for $[Ir(tmq)_2(\mathbf{L^8})]^{2+}$	S42

Table S1	Data collection parameters for the X-ray crystal structures	S43
Table S2	Selected redox data of the complexes	S44
Table S3	Calculated MO contributions, excited states descriptions and their associated transitions for $[Ru(bipy)_2(\mathbf{L^6})]^{3+}$	S45
Table S4	Calculated MO contributions, excited states descriptions and their associated transitions for $[Ru(bipy)_2(\mathbf{L^7})]^{3+}$	S46
Table S5	Calculated MO contributions, excited states descriptions and their associated transitions for $[Ru(bipy)_2(\mathbf{L^8})]^{3+}$	S47
Table S6	Calculated MO contributions, excited states descriptions and their associated transitions for $[Ir(tmq)_2(\mathbf{L^6})]^{2+}$	S48
Table S7	Calculated MO contributions, excited states descriptions and their associated transitions for $[Ir(tmq)_2(\mathbf{L^7})]^{2+}$	S48
Table S8	Calculated MO contributions, excited states descriptions and their associated transitions for $[Ir(tmq)_2(\mathbf{L^8})]^{2+}$	S49
Table S9	Superimposed DFT optimised S_0 (blue) and TD-DFT optimised S_1 (beige) geometries for all TPP^+ complexes, along with the root mean square deviation (RMSD) between the two structures.	S50

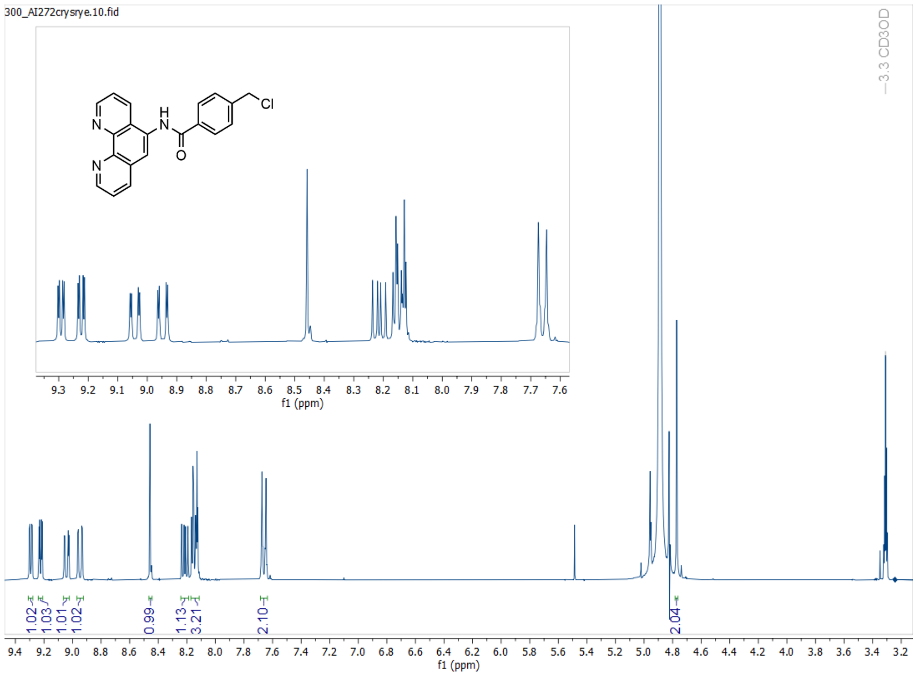

Figure S1. ^{1}H NMR spectrum of $\mathbf{L^1}$ in methanol-d4.

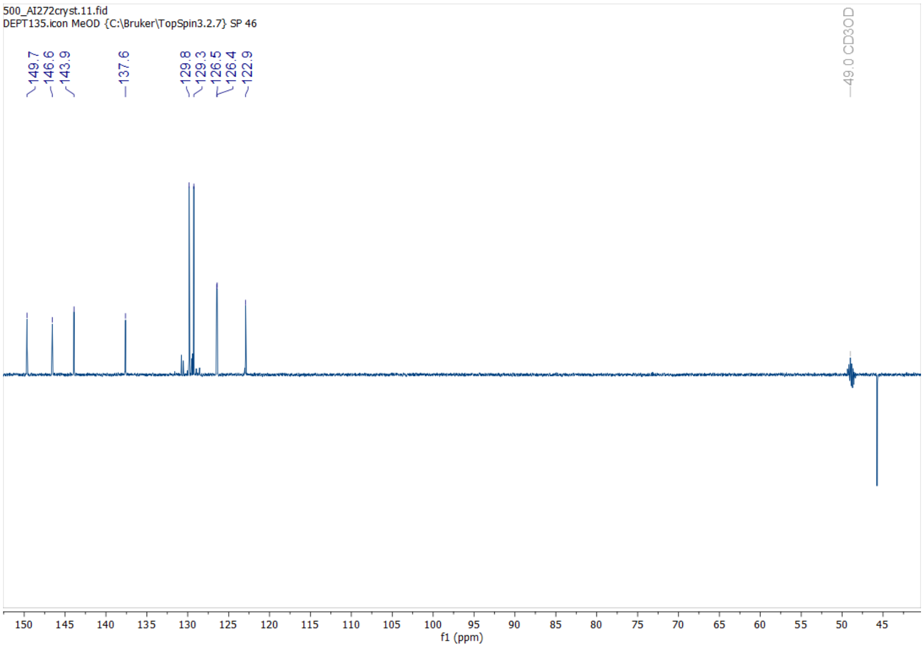

Figure S2. ^{13}C{^{1}H} NMR spectrum of $\mathbf{L^1}$ in methanol-d4.

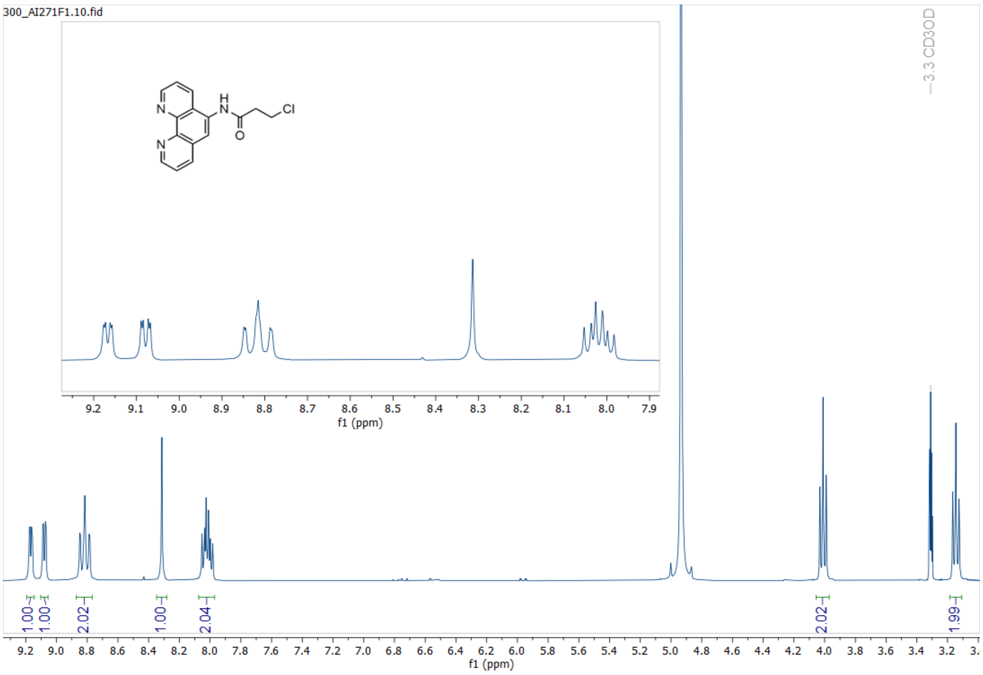

Figure S3. ^{1}H NMR spectrum of $\mathbf{L^2}$ in methanol-d4.

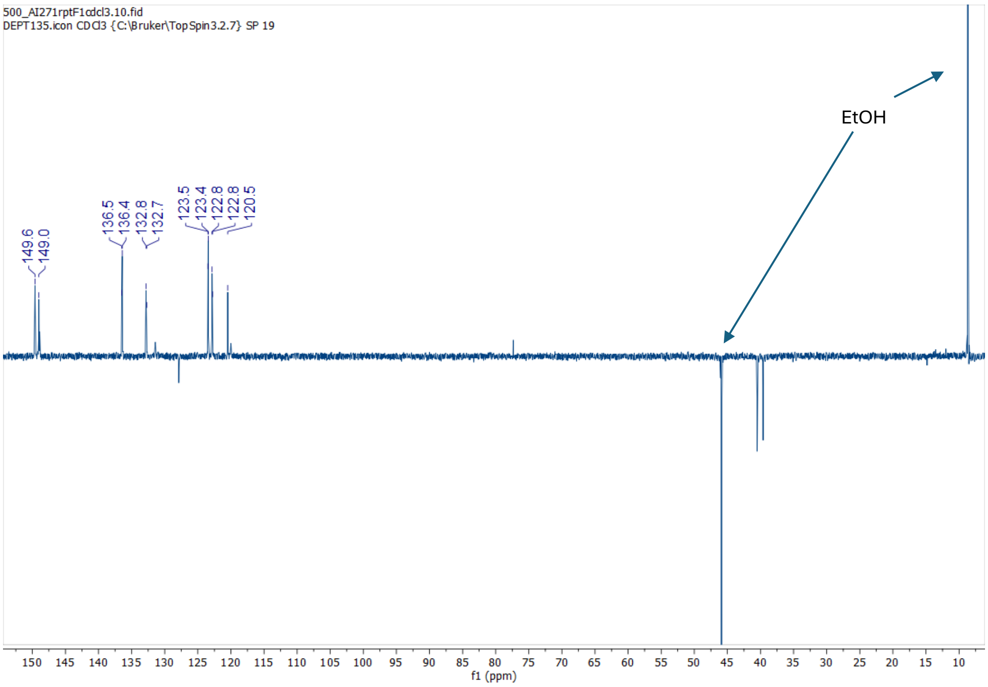

Figure S4. $^{13}C\{^{1}H\}$ NMR spectrum of $\mathbf{L^2}$ in $CDCl_3$.

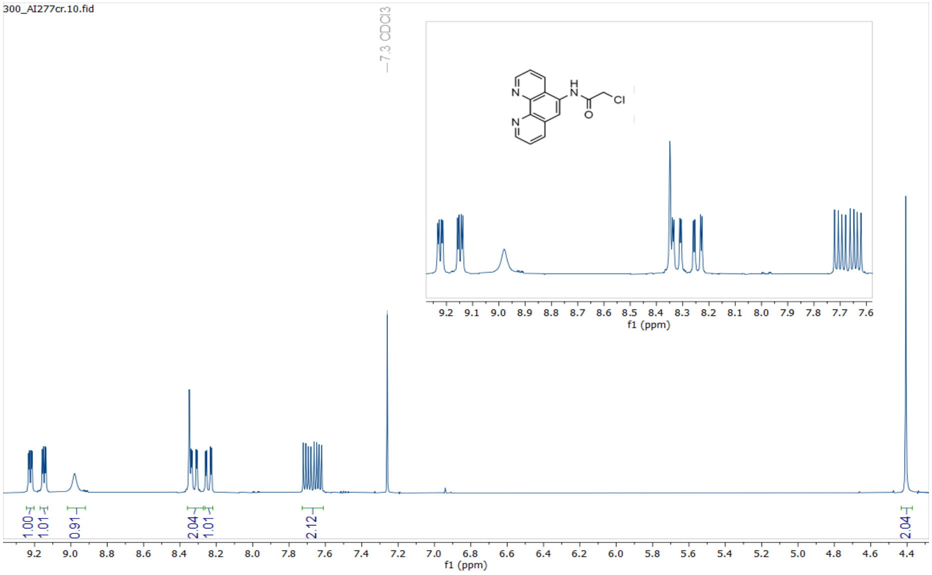

Figure S5. ^{1}H NMR spectrum of **L³** in $CDCl_3$.

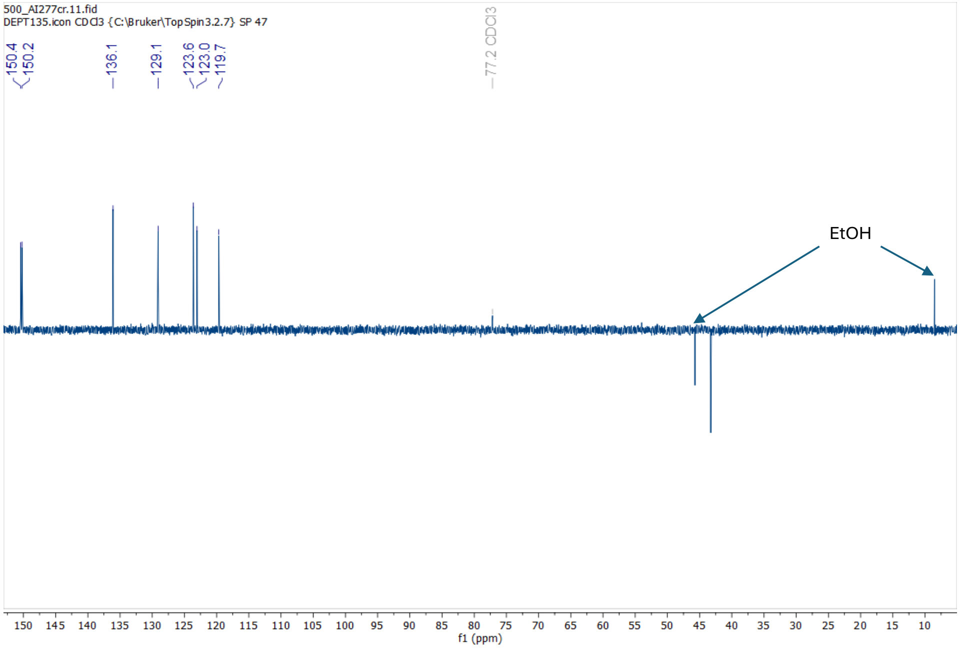

Figure S6. $^{13}C\{^{1}H\}$ NMR spectrum of **L³** in $CDCl_3$.

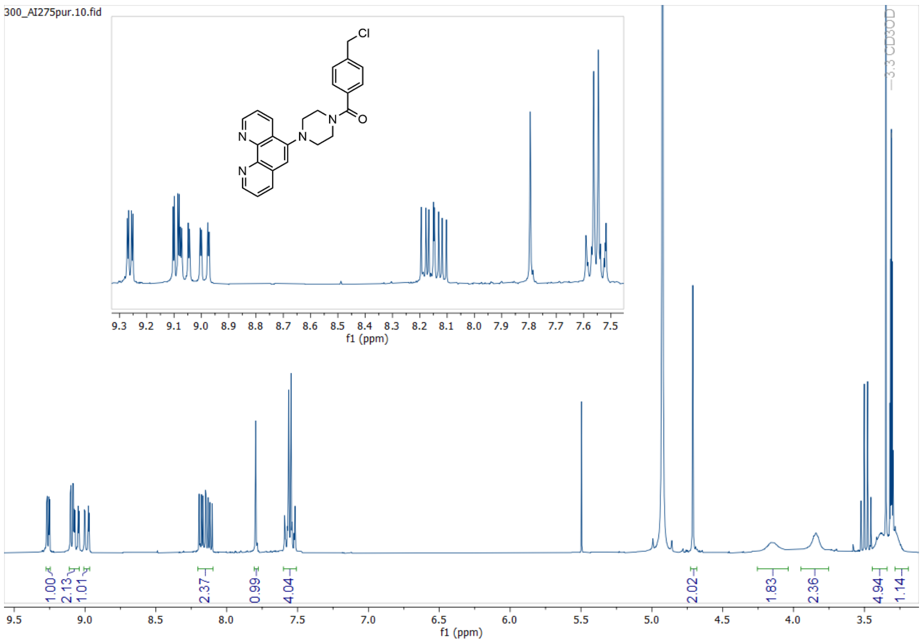

Figure S7. ^{1}H NMR spectrum of **L[4]** in methanol-d4.

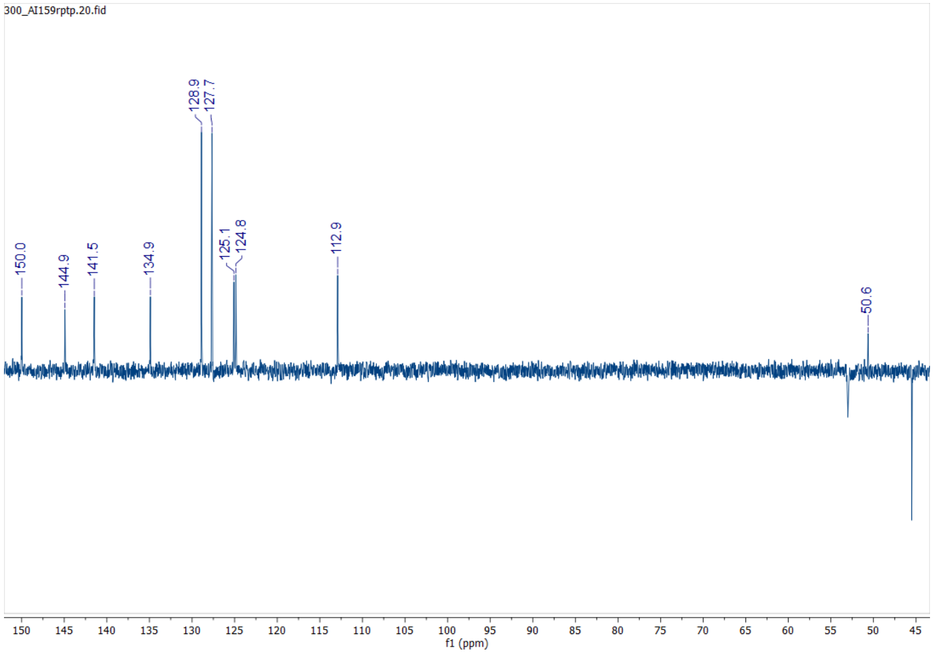

Figure S8. $^{13}C\{^{1}H\}$ NMR spectrum of **L[4]** in methanol-d4.

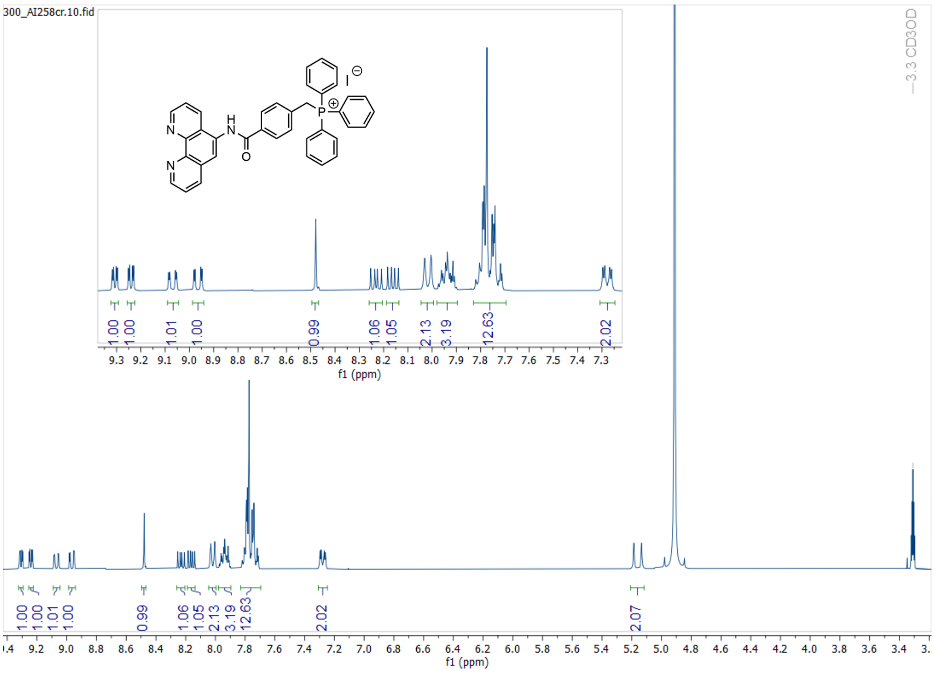

Figure S9. ^{1}H NMR spectrum of $\mathbf{L^5}$ in methanol-d4.

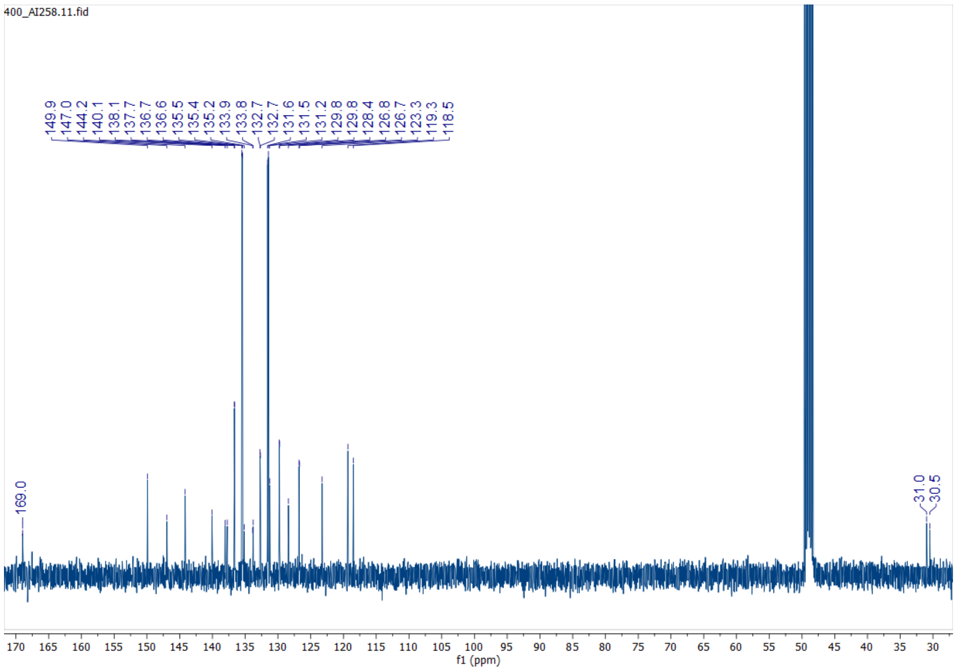

Figure S10. ^{13}C{^{1}H} NMR spectrum of $\mathbf{L^5}$ in methanol-d4.

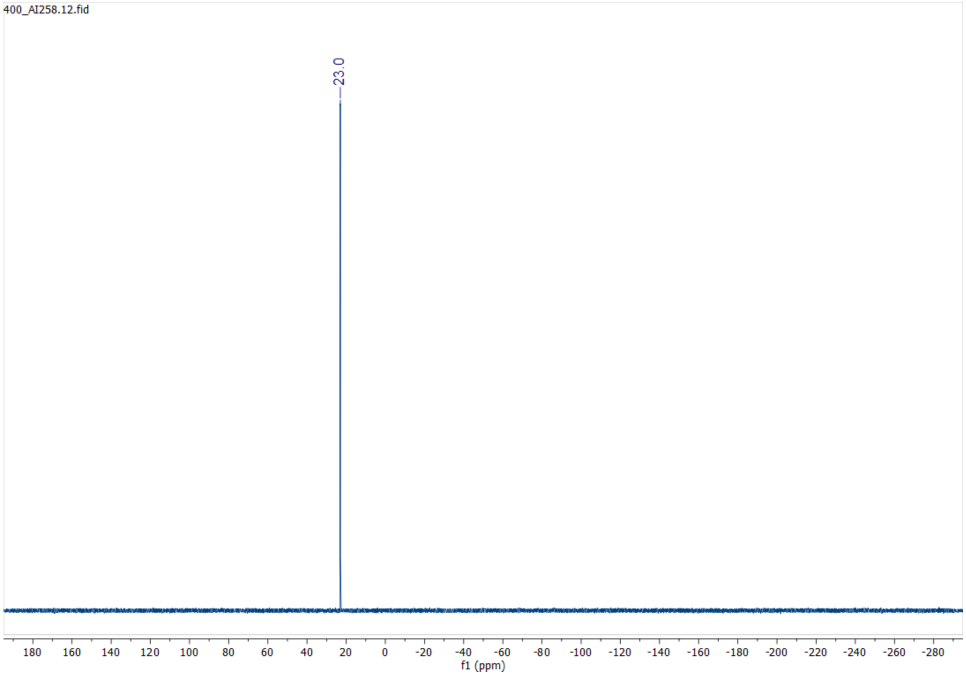

Figure S11. $^{31}P\{^{1}H\}$ NMR spectrum of **L[5]** methanol-d4.

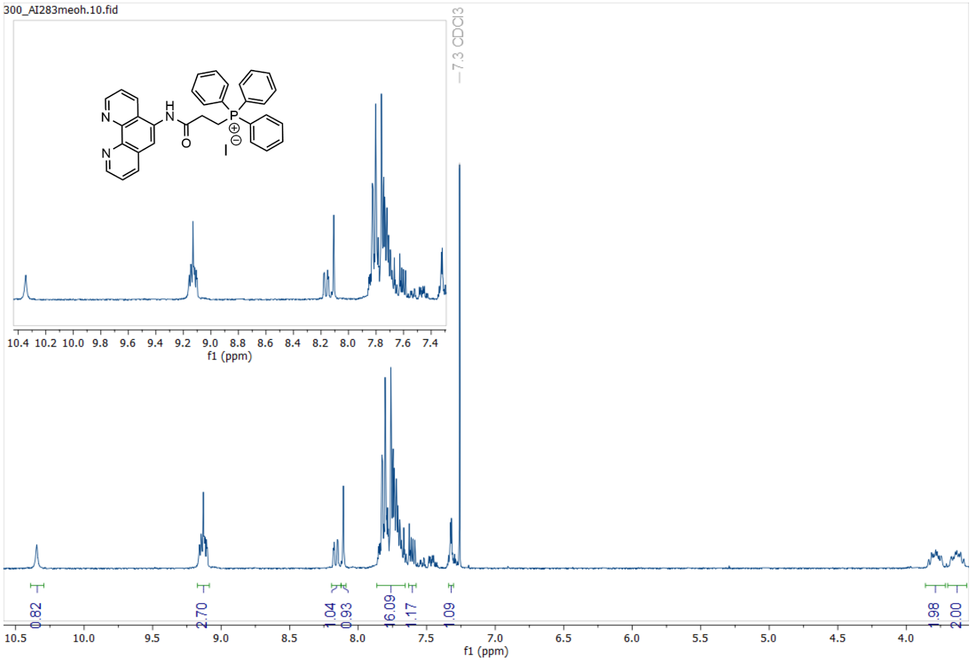

Figure S12. ^{1}H NMR spectrum of **L[6]** in methanol-d4.

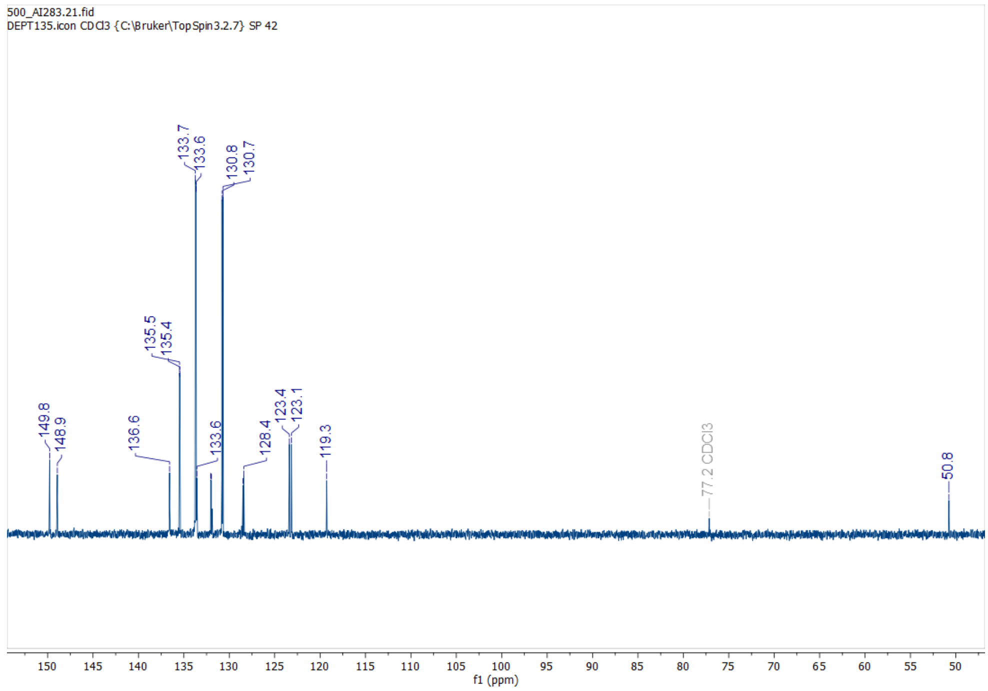

Figure S13. $^{13}C\{^{1}H\}$ NMR spectrum of $\mathbf{L^6}$ in chloroform

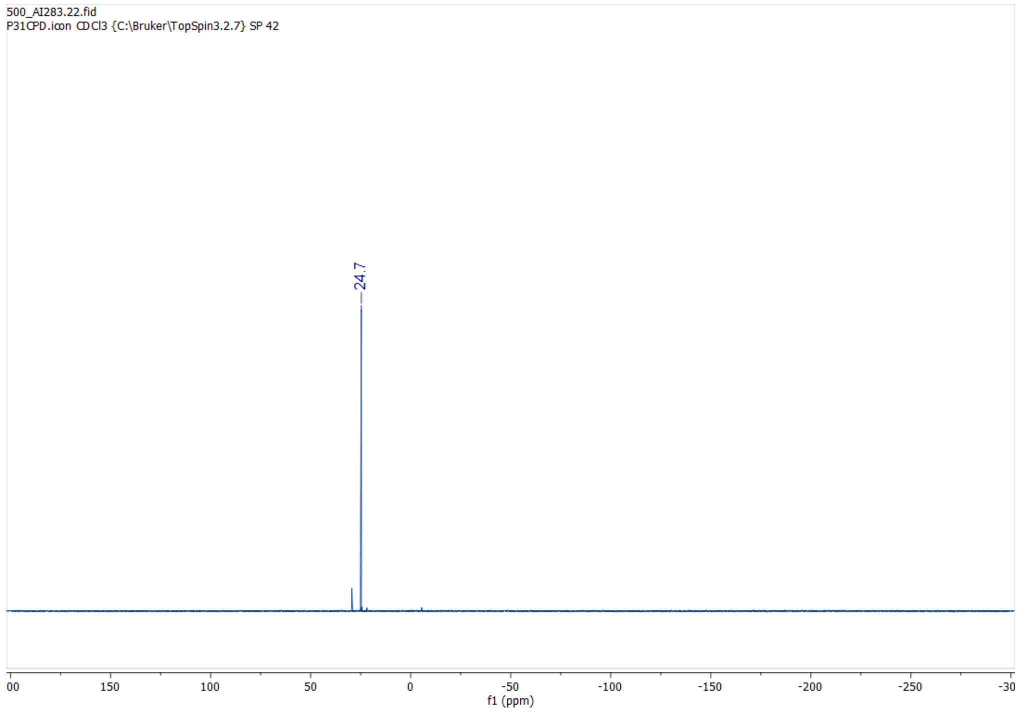

Figure S14. $^{31}P\{^{1}H\}$ NMR spectrum of $\mathbf{L^6}$ methanol-d4.

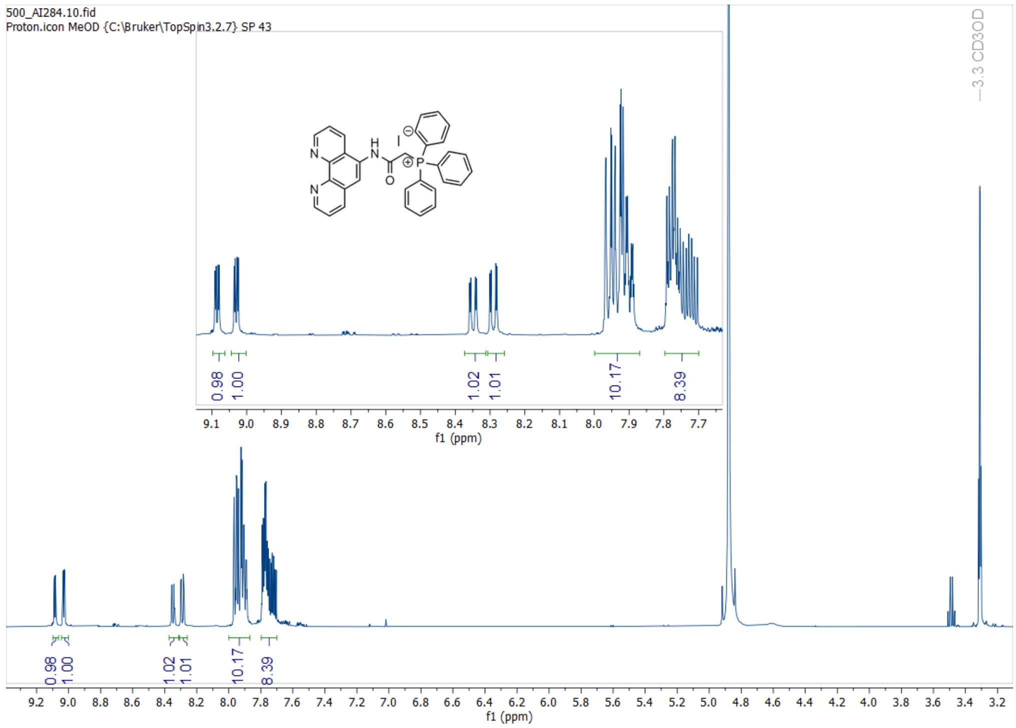

Figure S15. ^{1}H NMR spectrum of **L^{7}** in methanol-d4.

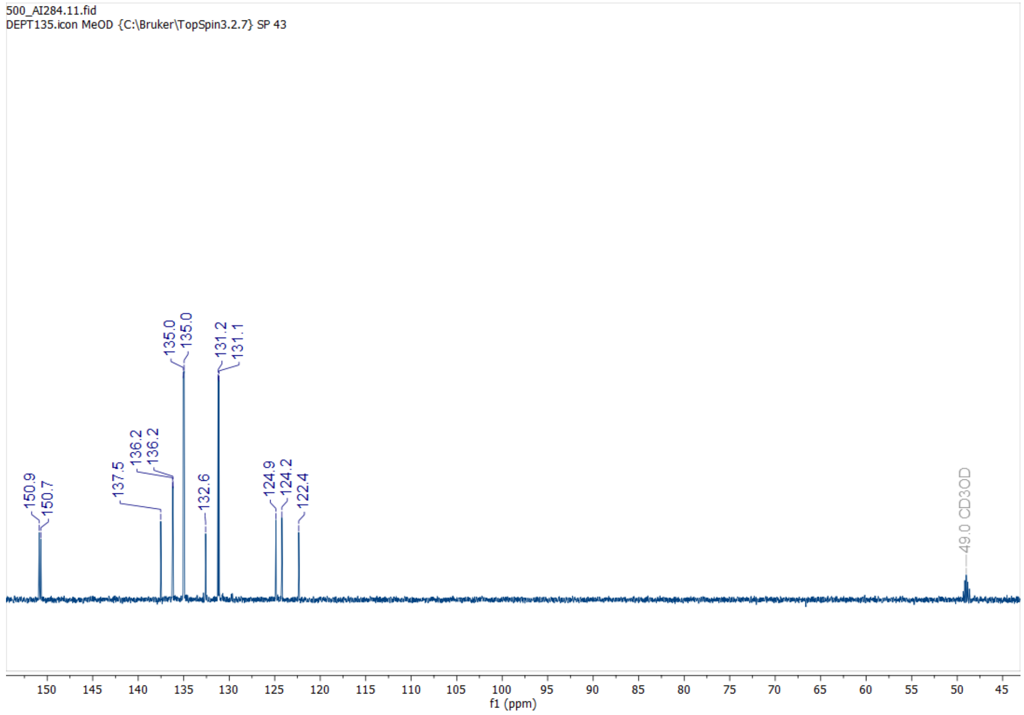

Figure S16. ^{13}C{^{1}H} NMR spectrum of **L^{7}** in methanol-d4.

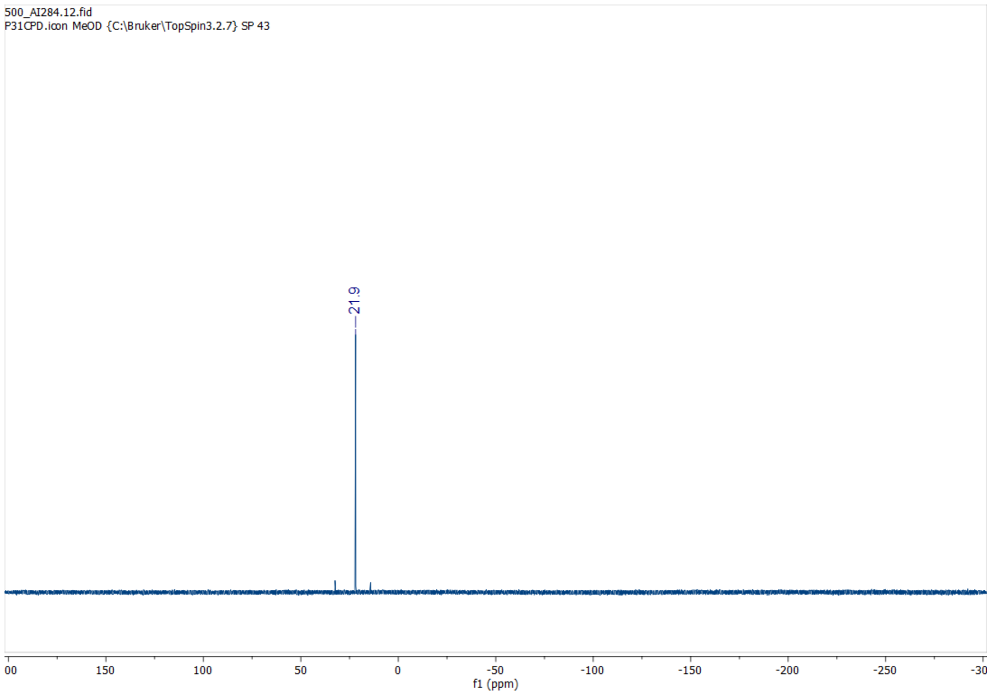

Figure S17. $^{31}P\{^{1}H\}$ NMR spectrum of **L^{7}** methanol-d4.

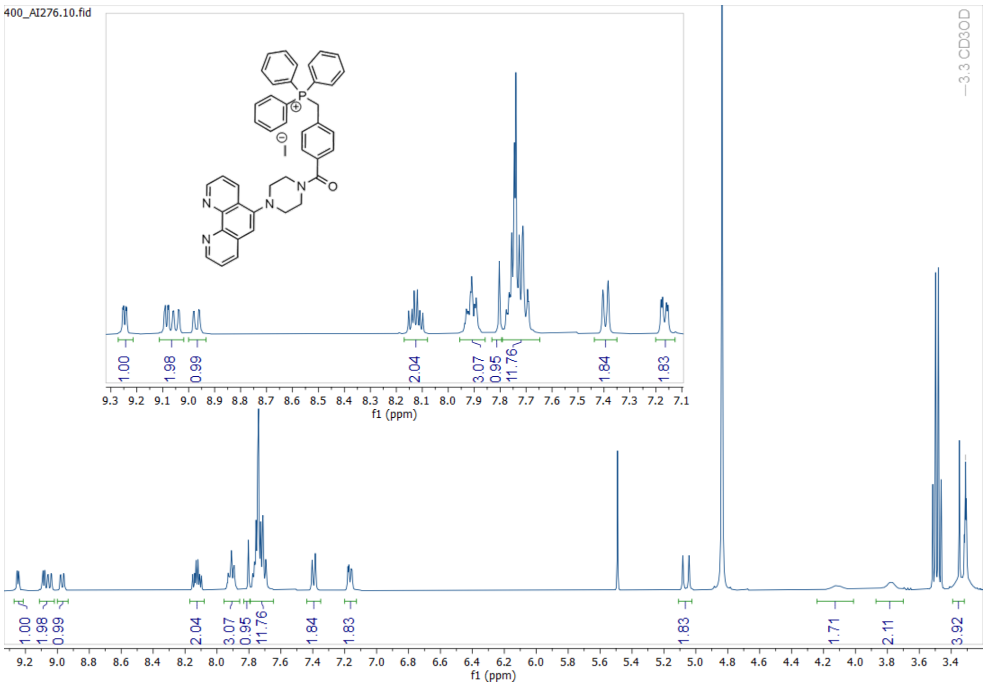

Figure S18. ^{1}H NMR spectrum of **L^{8}** in methanol-d4.

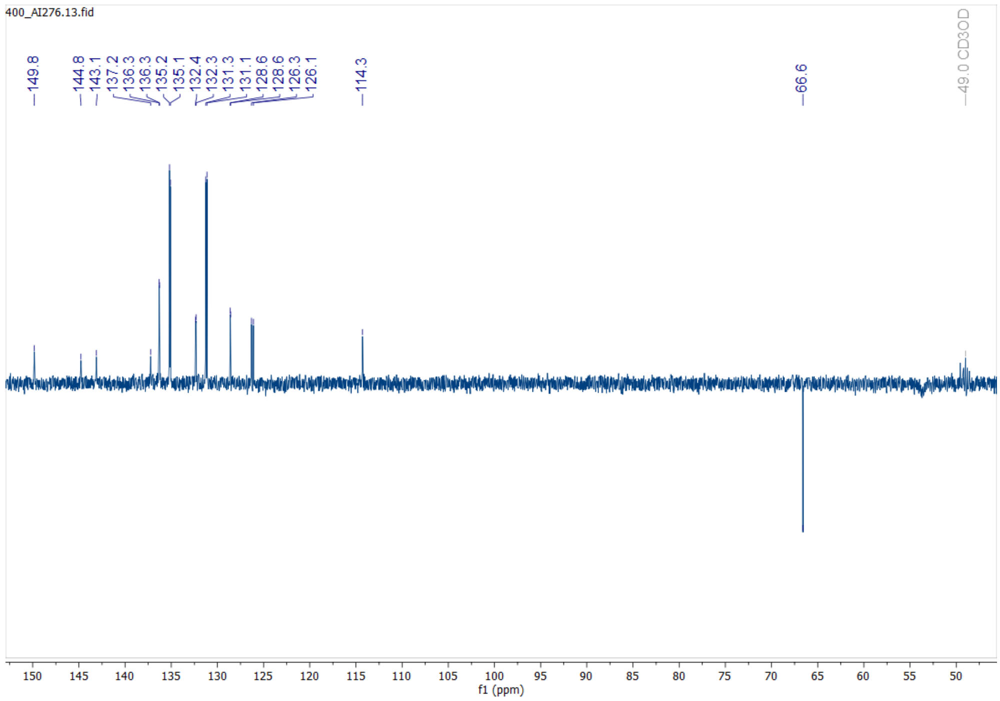

Figure S19. $^{13}C\{^1H\}$ NMR spectrum of $\mathbf{L^8}$ in methanol-d4.

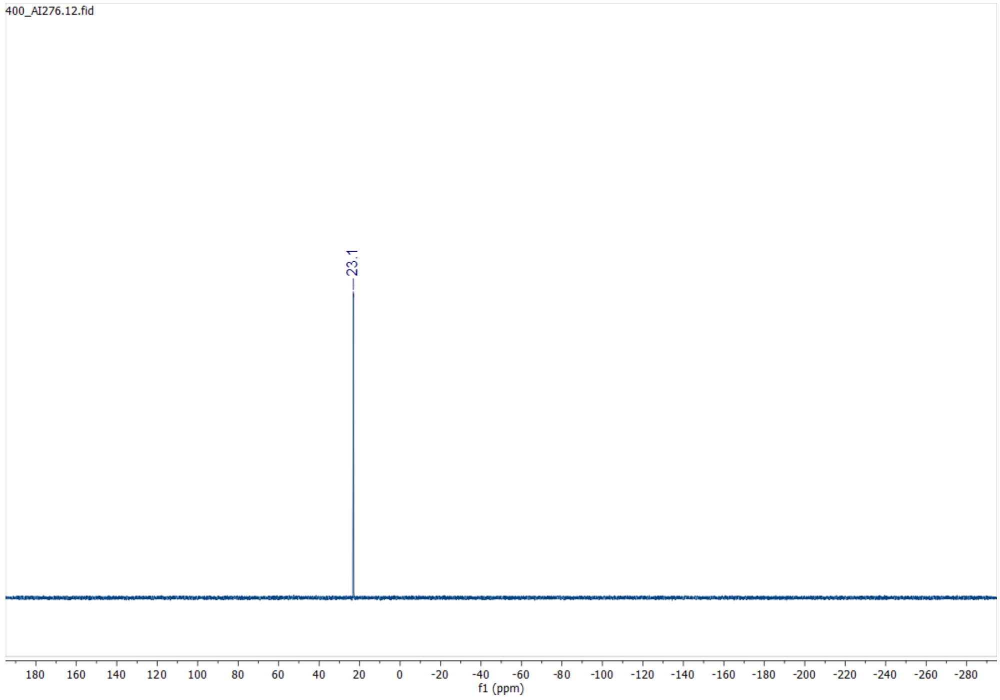

Figure S20. $^{31}P\{^1H\}$ NMR spectrum of $\mathbf{L^8}$ methanol-d4.

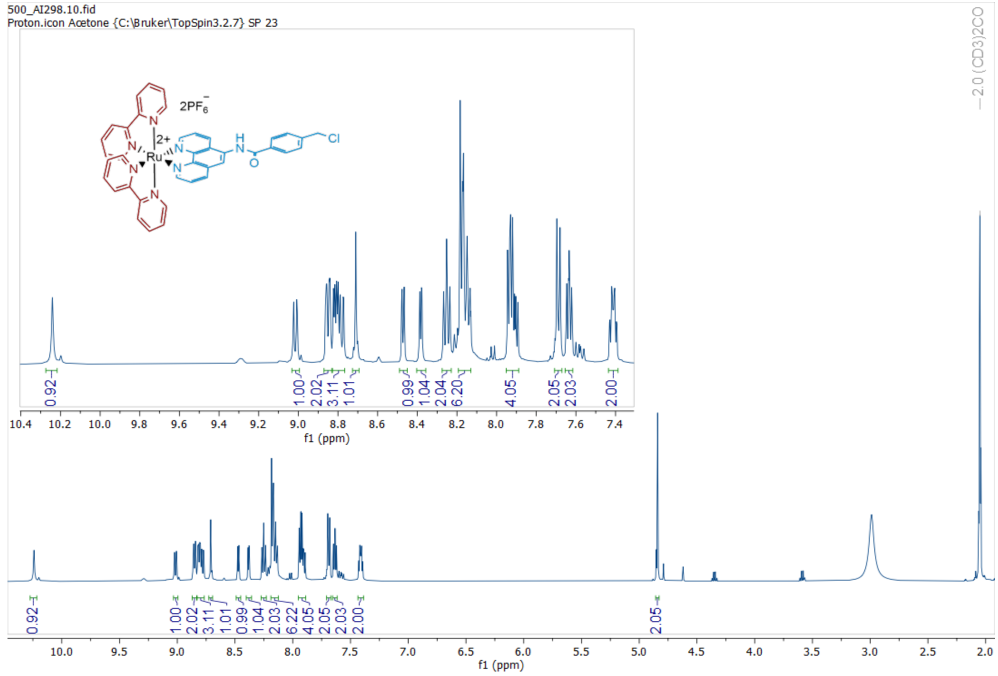

Figure S21. ^{1}H NMR spectrum of [Ru(bipy)$_2$(**L^1**)](PF$_6$)$_3$ in acetone-d6.

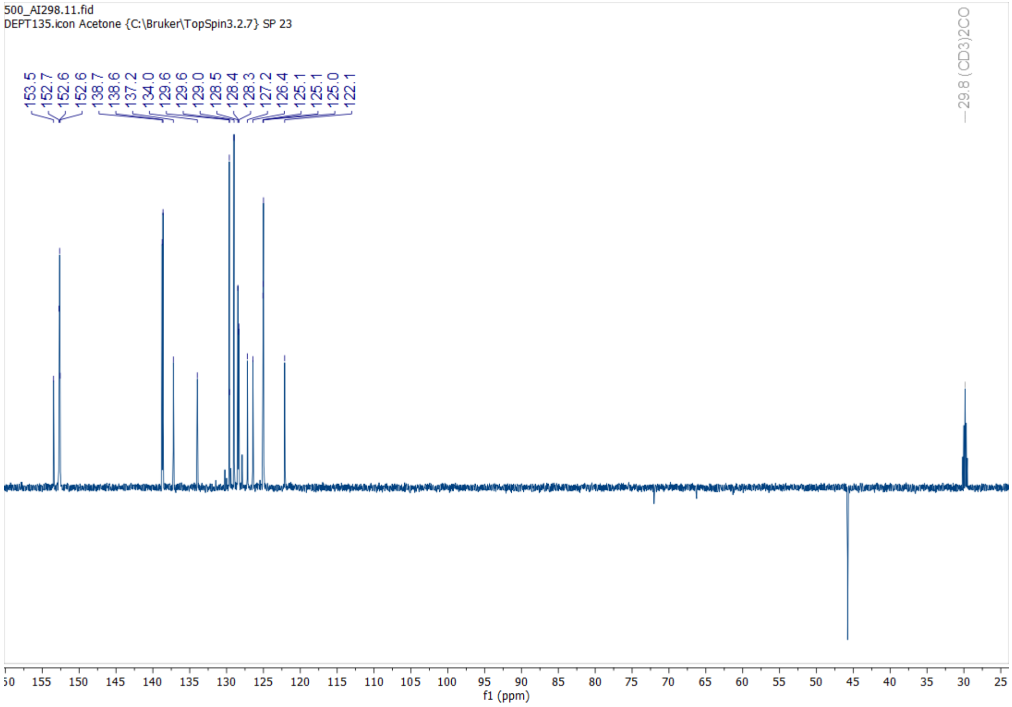

Figure S22. ^{13}C{^{1}H} NMR spectrum of [Ru(bipy)$_2$(**L^1**)](PF$_6$)$_2$ in acetone-d6.

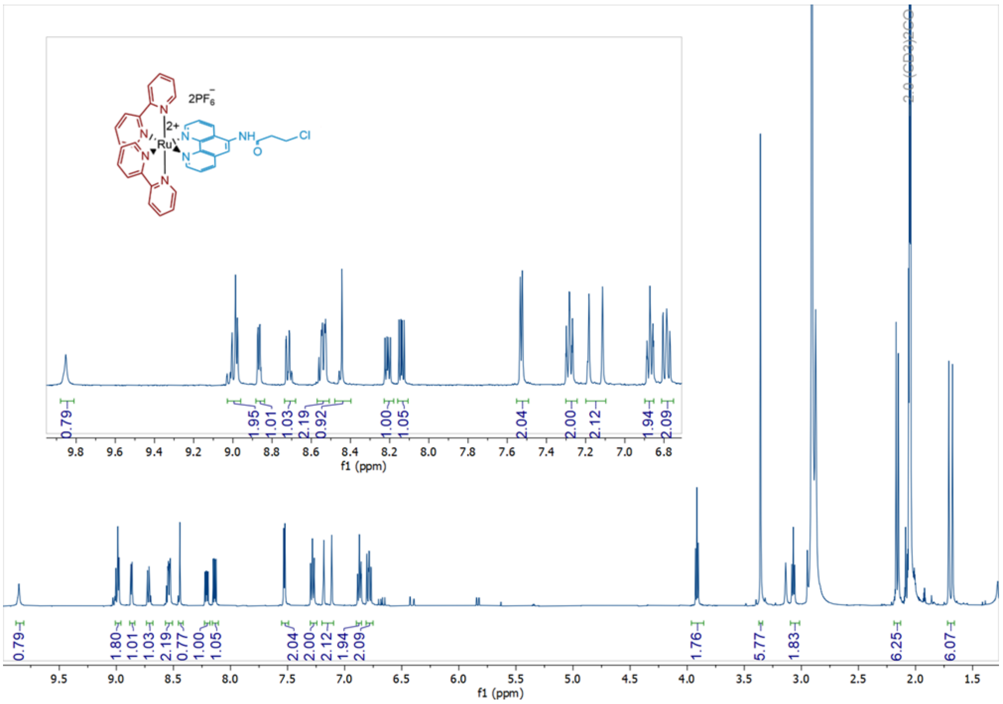

Figure S23. ^{1}H NMR spectrum of [Ru(bipy)$_2$(**L^2**)](PF$_6$)$_3$ in acetonitrile-d3.

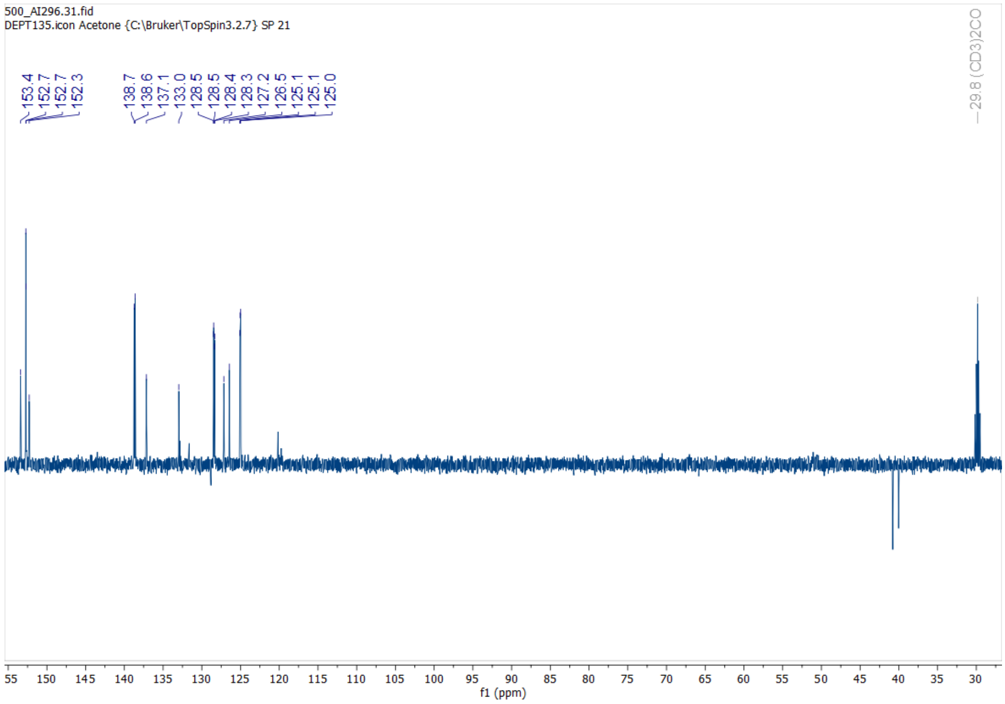

Figure S24. ^{13}C{^{1}H} NMR spectrum of [Ru(bipy)$_2$(**L^2**)](PF$_6$)$_2$ in acetonitrile-d3.

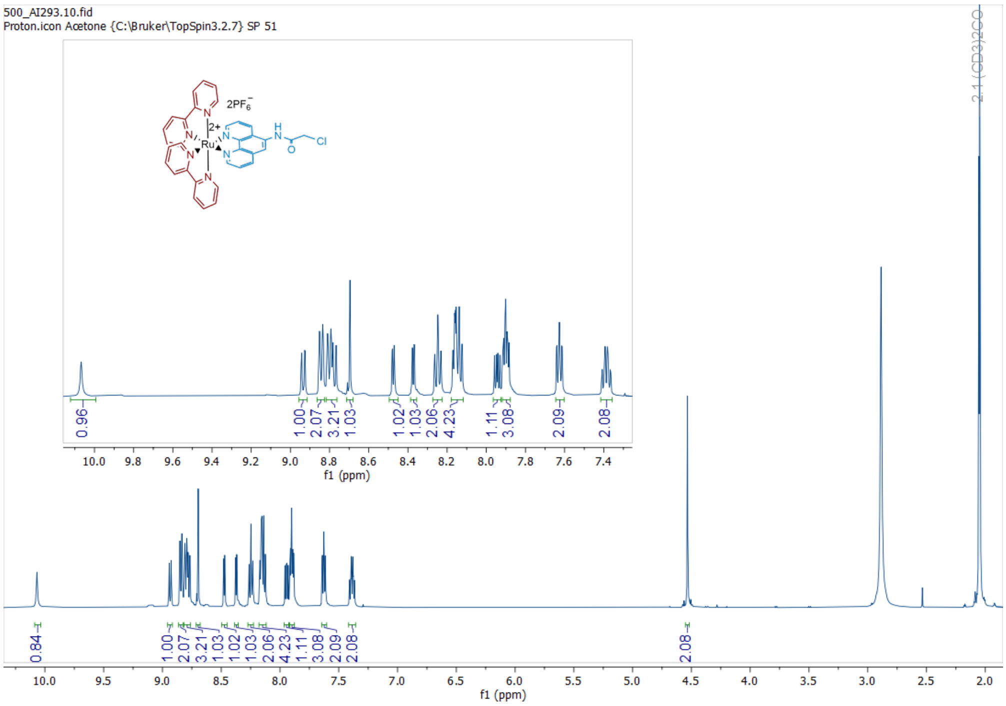

Figure S25. ^{1}H NMR spectrum of $[Ru(bipy)_2(\mathbf{L^3})](PF_6)_2$ in acetone-d6.

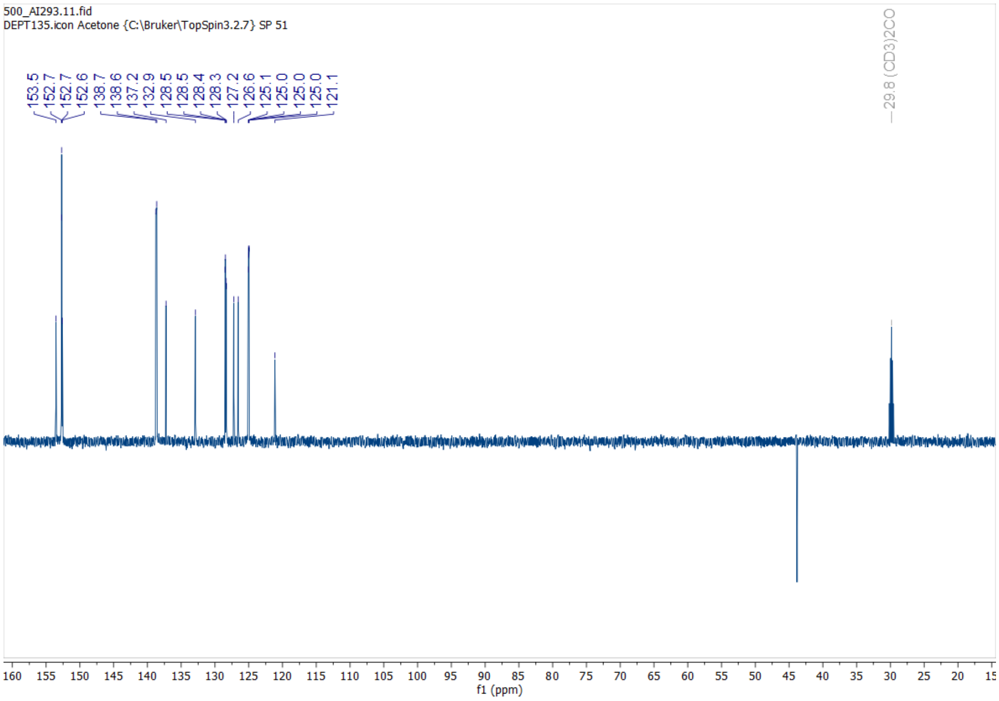

Figure S26. ^{13}C{^{1}H} NMR spectrum of $[Ru(bipy)_2(\mathbf{L^3})](PF_6)_2$ in acetonitrile-d3.

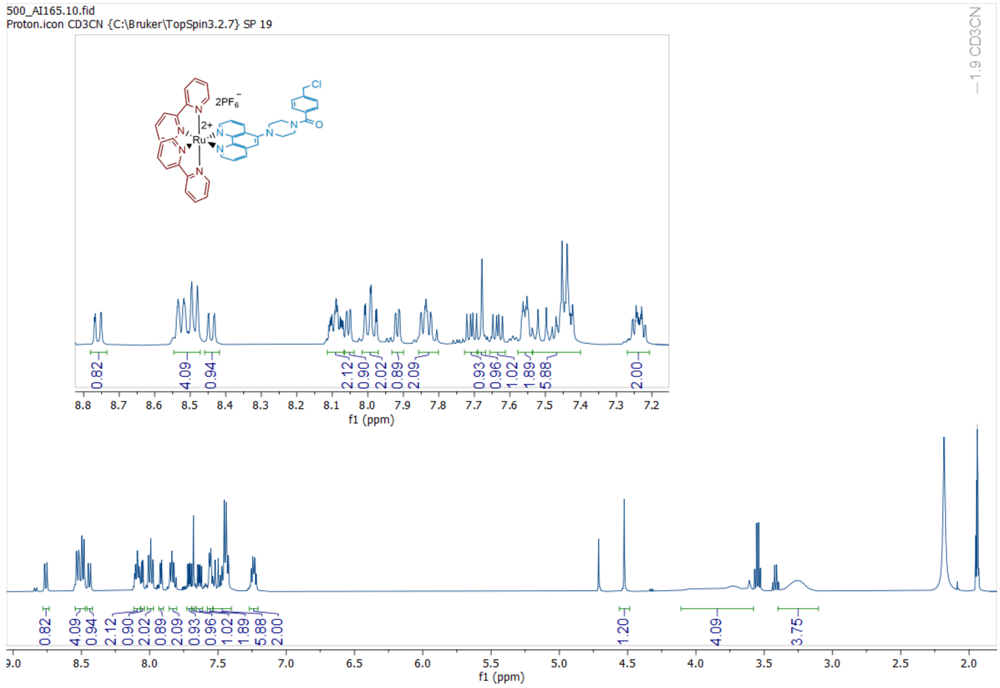

Figure S27. ^{1}H NMR spectrum of [Ru(bipy)$_2$(**L**^{4})](PF$_6$)$_2$ in acetonitrile-d3.

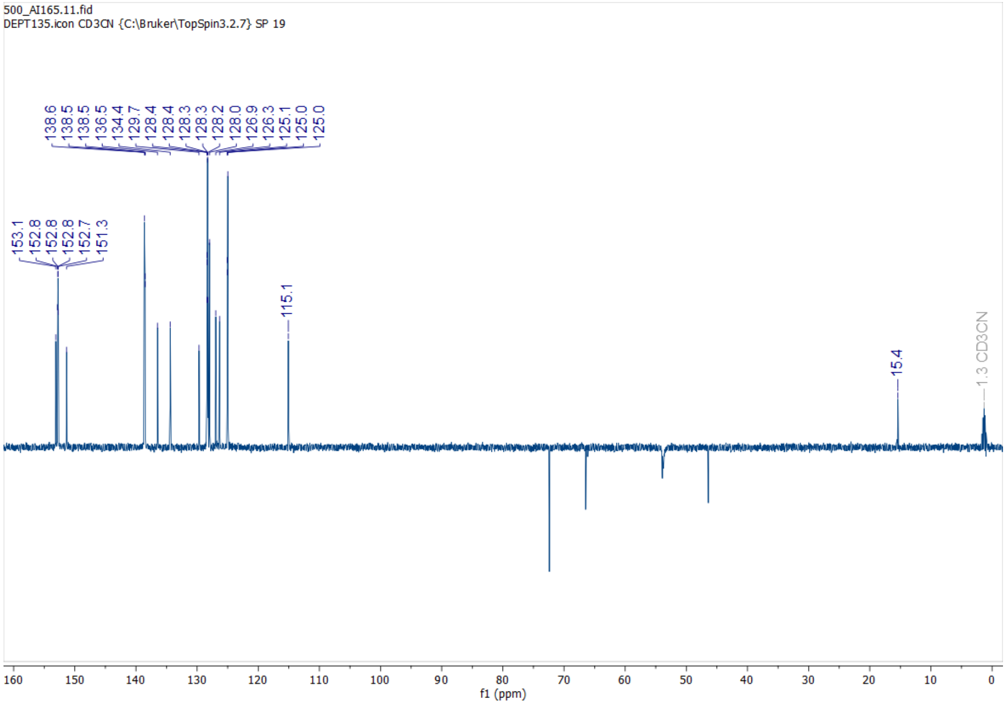

Figure S28. ^{13}C{^{1}H} NMR spectrum of [Ru(bipy)$_2$(**L**^{4})](PF$_6$)$_2$ in acetonitrile-d3.

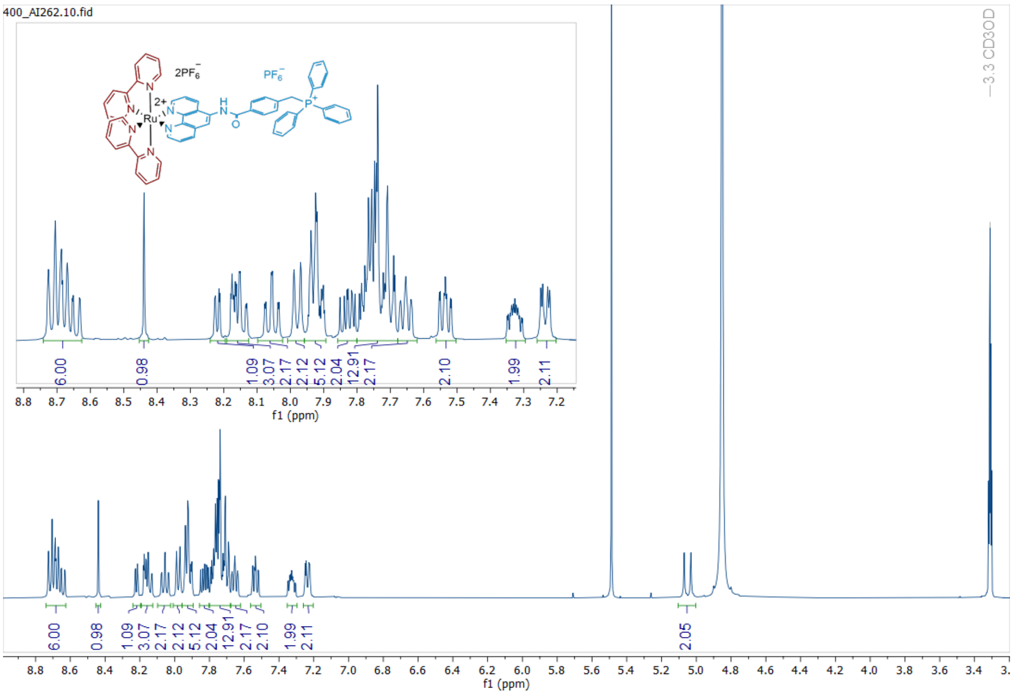

Figure S29. ^{1}H NMR spectrum of $[Ru(bipy)_2(\mathbf{L^5})](PF_6)_3$ in methanol-d4.

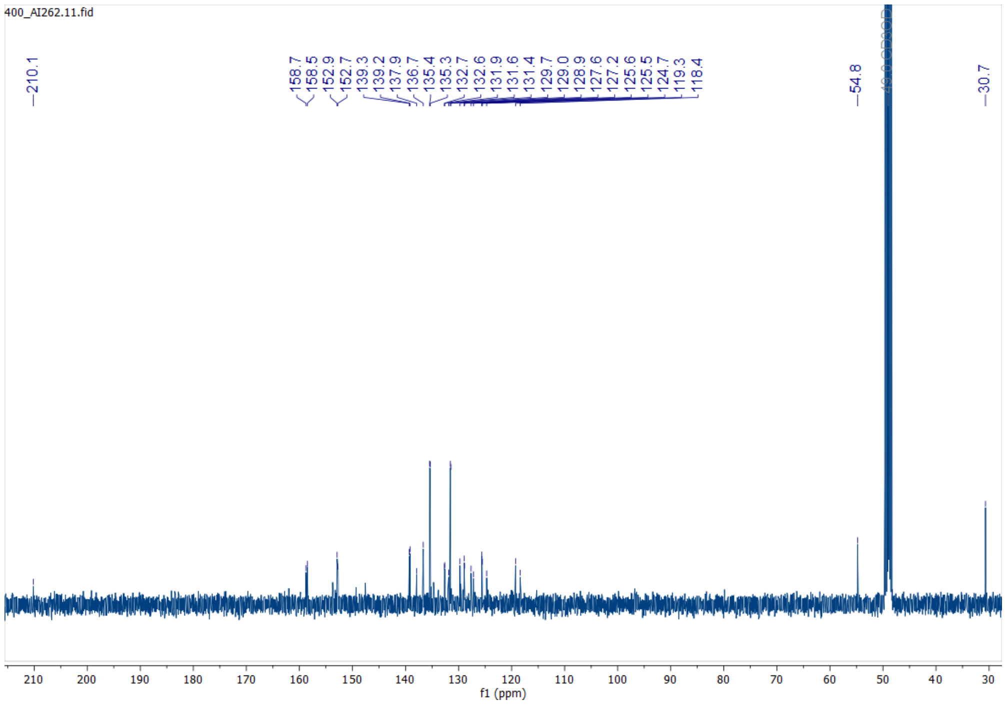

Figure S30. $^{13}C\{^{1}H\}$ NMR spectrum of $[Ru(bipy)_2(\mathbf{L^5})](PF_6)_3$ in acetonitrile-d3.

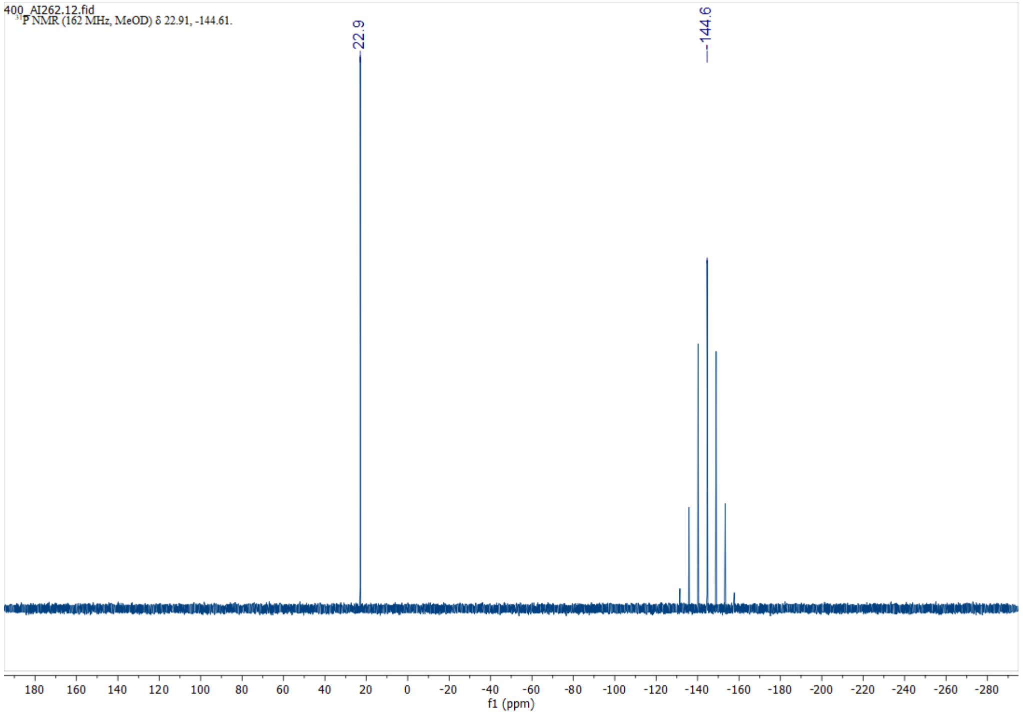

Figure S31. $^{31}P\{^{1}H\}$ NMR spectrum of [Ru(bipy)$_2$(**L^5**)](PF$_6$)$_3$ acetonitrile-d6.

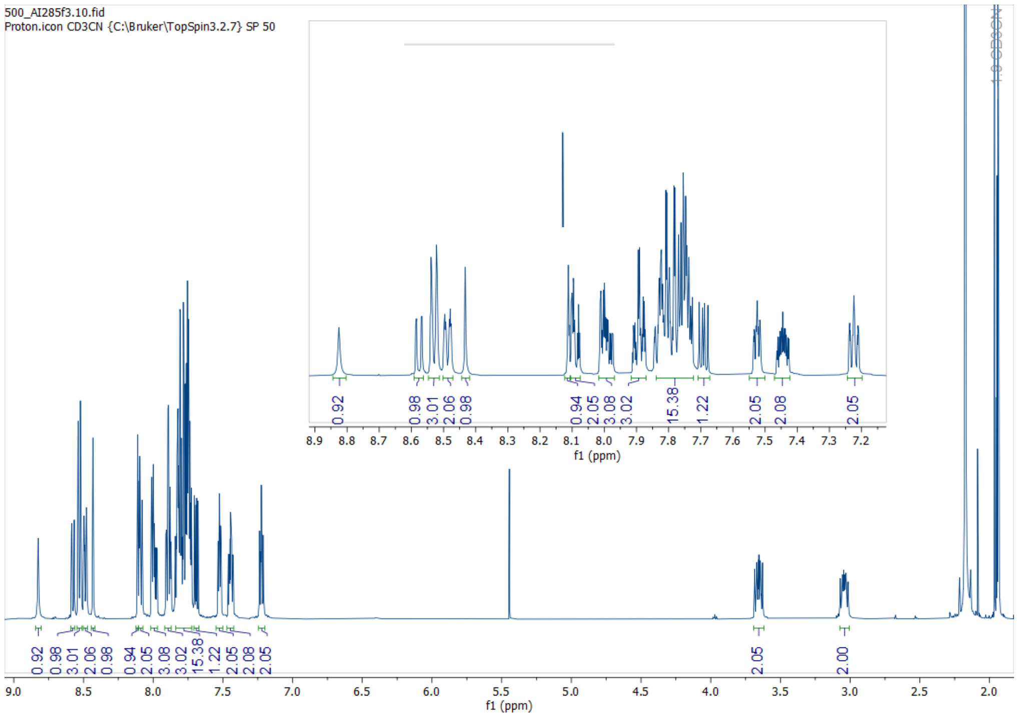

Figure S32. ^{1}H NMR spectrum of [Ru(bipy)$_2$(**L^6**)](PF$_6$)$_3$ in acetonitrile-d3.

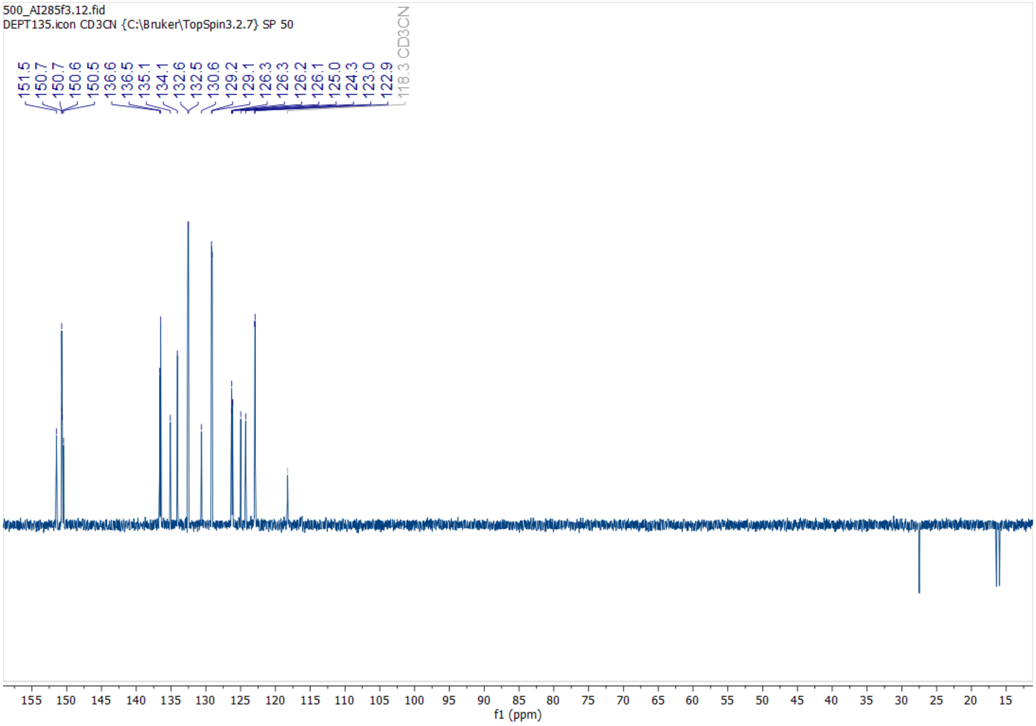

Figure S33. $^{13}C\{^{1}H\}$ NMR spectrum of $[Ru(bipy)_2(\mathbf{L^6})](PF_6)_3$ in acetone-d6.

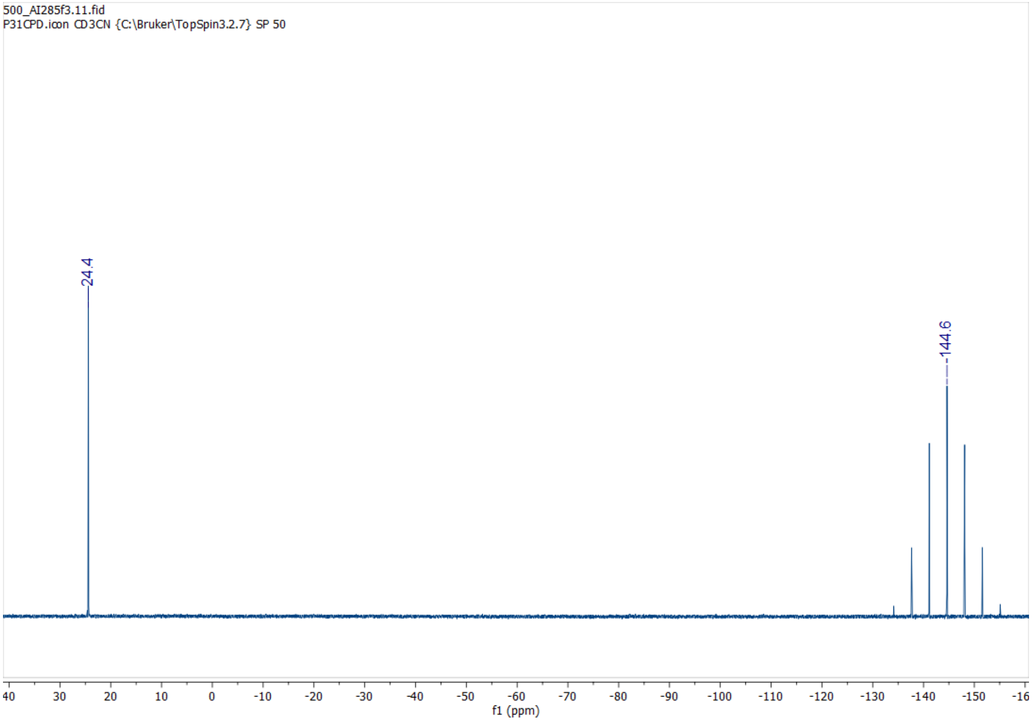

Figure S34. $^{31}P\{^{1}H\}$ NMR spectrum of $[Ru(bipy)_2(\mathbf{L^6})](PF_6)_3$ acetonitrile-d6.

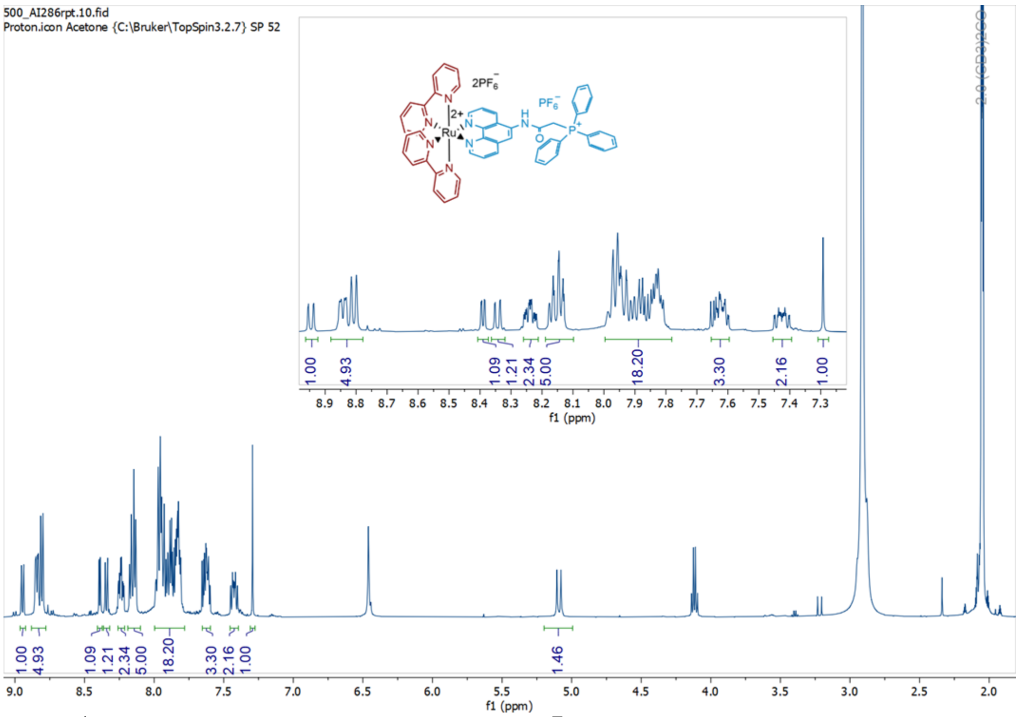

Figure S35. ^{1}H NMR spectrum of $[Ru(bipy)_2(\mathbf{L^7})](PF_6)_3$ in methanol-d6.

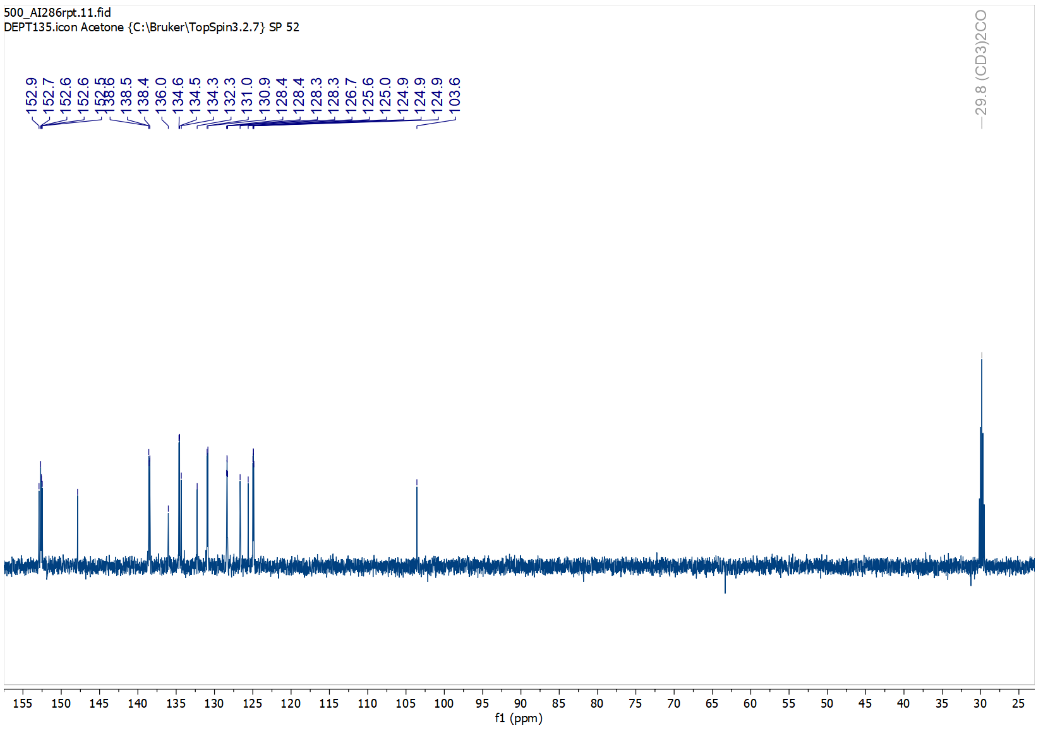

Figure S36. $^{13}C\{^{1}H\}$ NMR spectrum of $[Ru(bipy)_2(\mathbf{L^7})](PF_6)_3$ in acetone-d6.

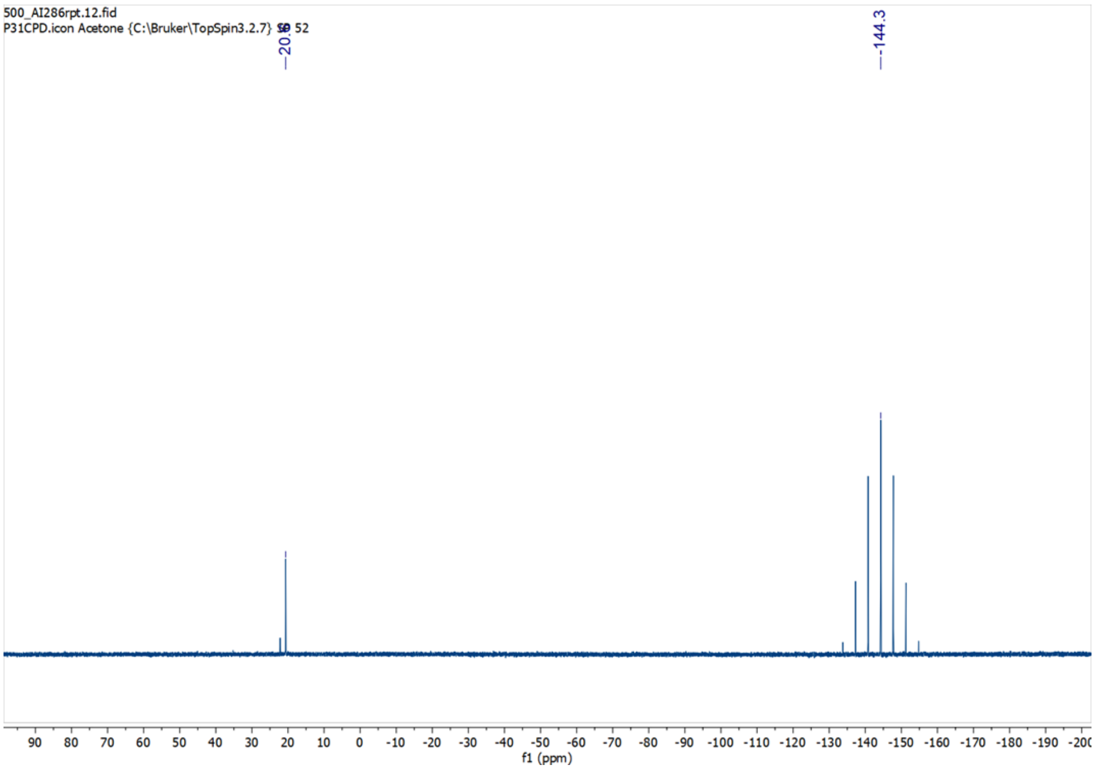

Figure S37. $^{31}P\{^{1}H\}$ NMR spectrum of $[Ru(bipy)_2(\mathbf{L^7})](PF_6)_3$ acetone-d6.

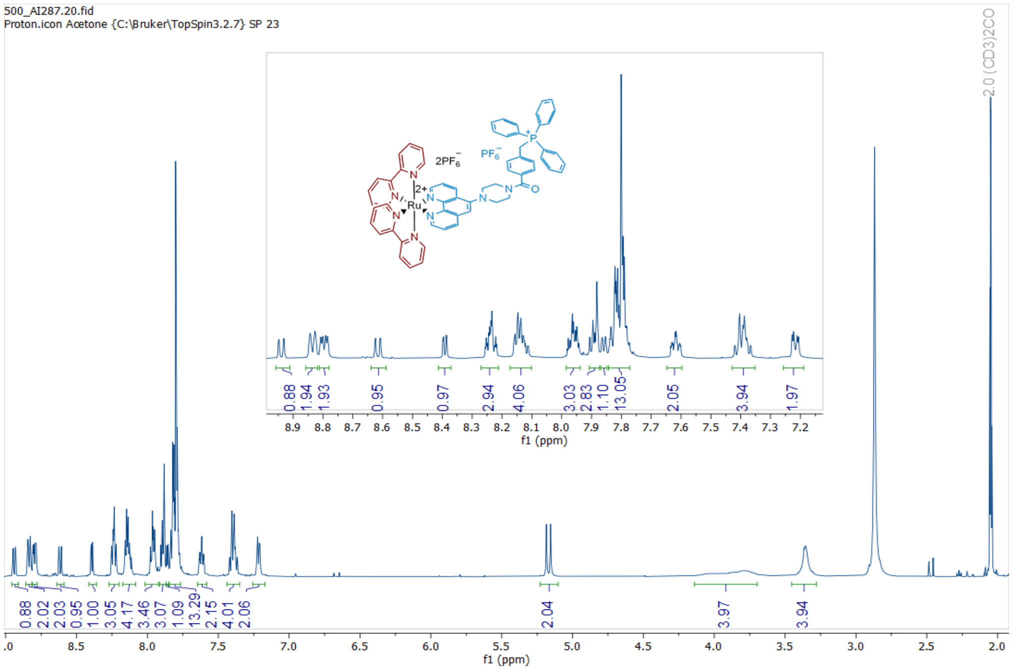

Figure S38. ^{1}H NMR spectrum of $[Ru(bipy)_2(\mathbf{L^8})](PF_6)_3$ acetone-d6.

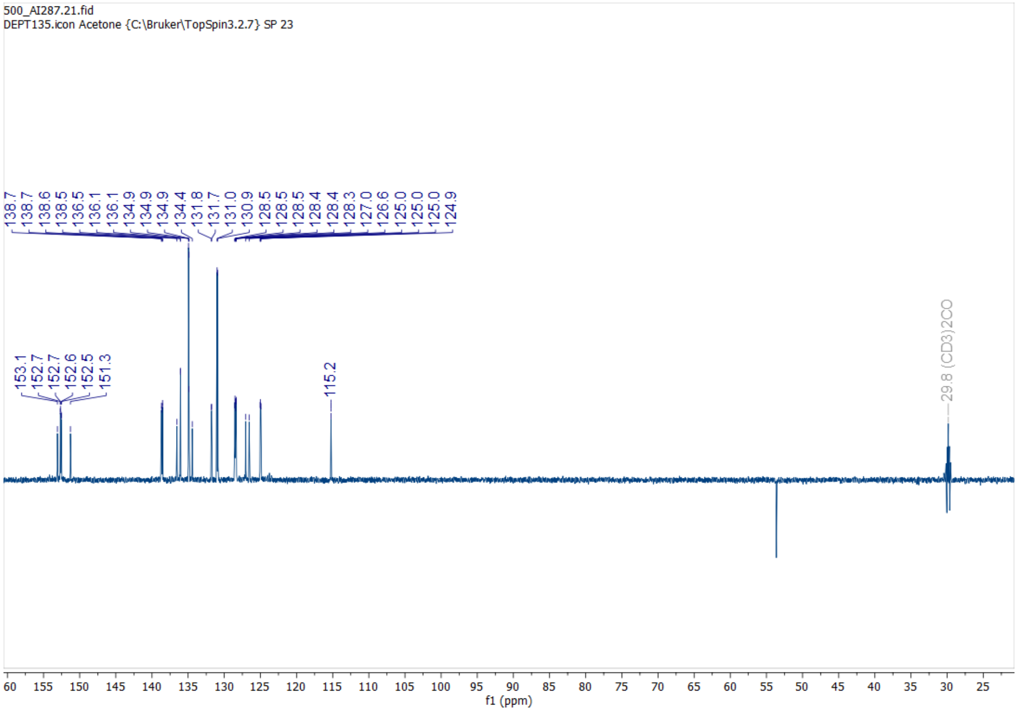

Figure S39. $^{13}C\{^1H\}$ NMR spectrum of $[Ru(bipy)_2($**L8**$)](PF_6)_3$ acetone-d6.

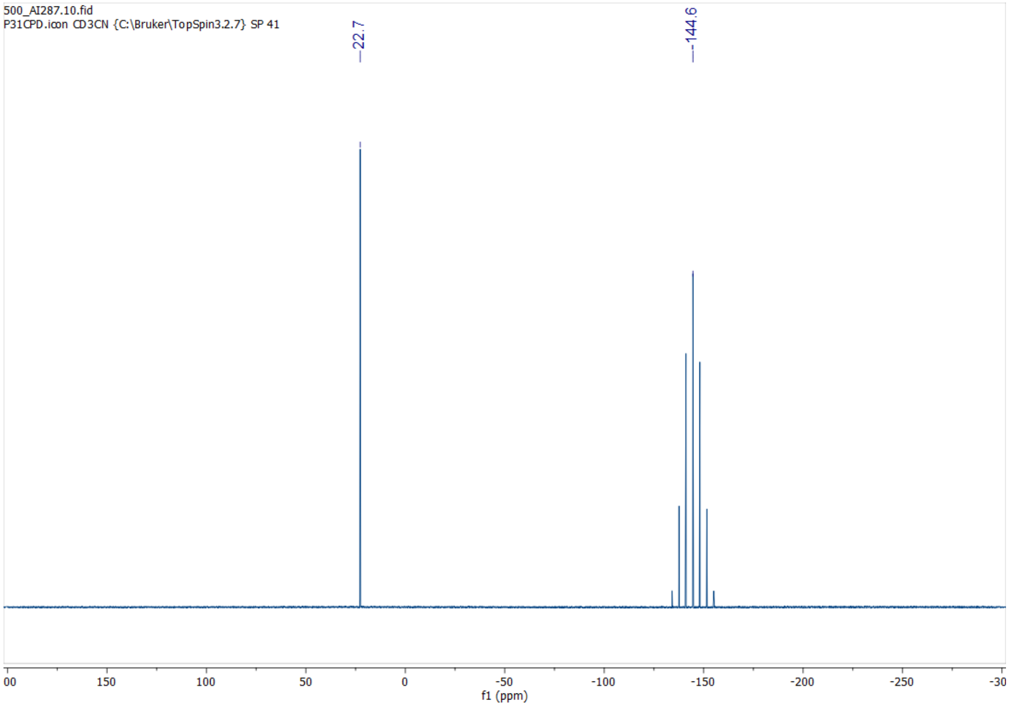

Figure S40. $^{31}P\{^1H\}$ NMR spectrum of $[Ru(bipy)_2($**L^8**$)](PF_6)_3$ acetone-d6.

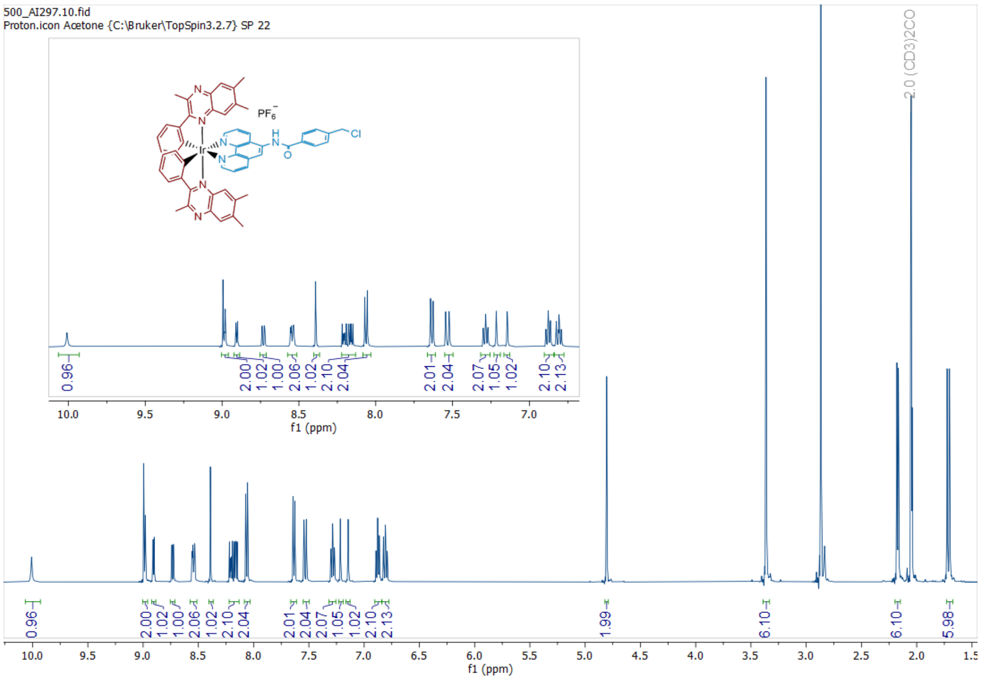

Figure S41. ^{1}H NMR spectrum of $[Ir(tmq)_2(\mathbf{L^1})](PF_6)$ acetone-d6.

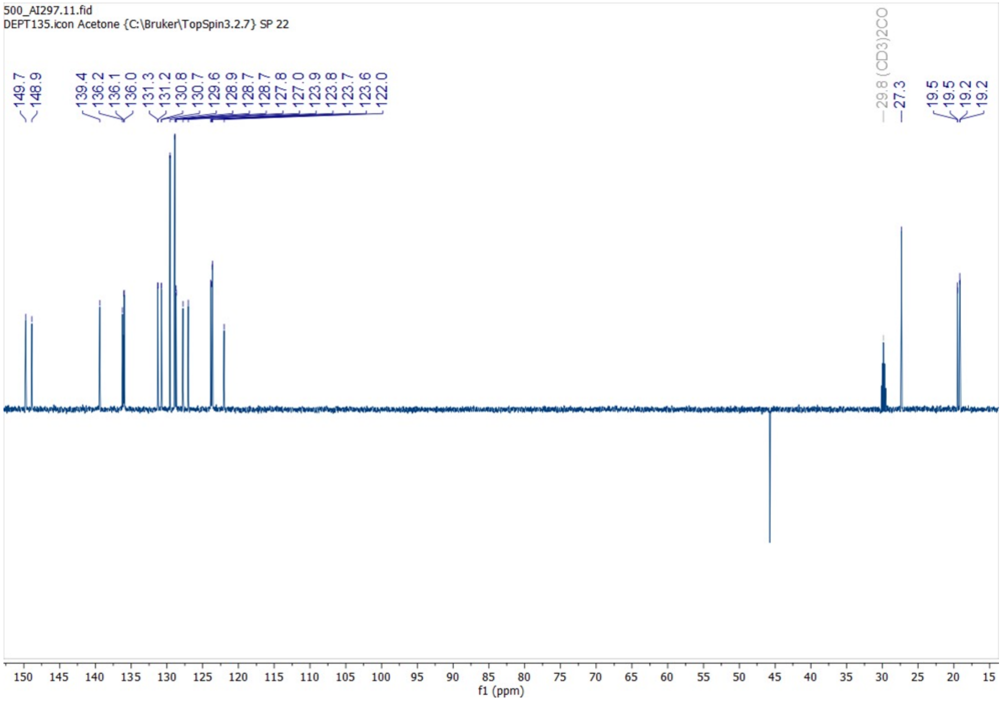

Figure S42. $^{13}C\{^{1}H\}$ NMR spectrum of $[Ir(tmq)_2(\mathbf{L^1})](PF_6)$ acetone-d6.

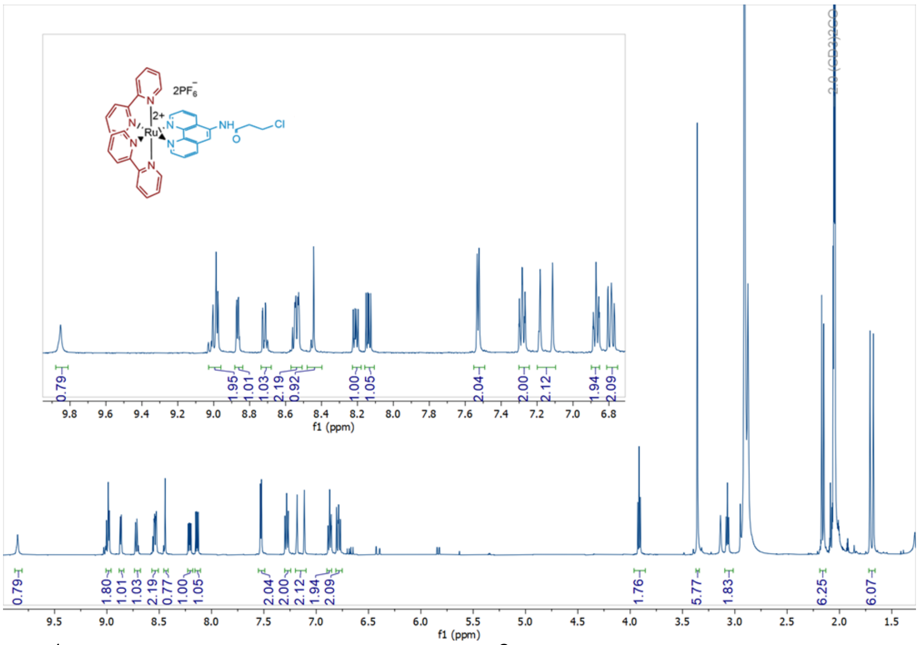

Figure S43. ^{1}H NMR spectrum of $[Ir(tmq)_2(\mathbf{L^2})](PF_6)$ acetone-d6.

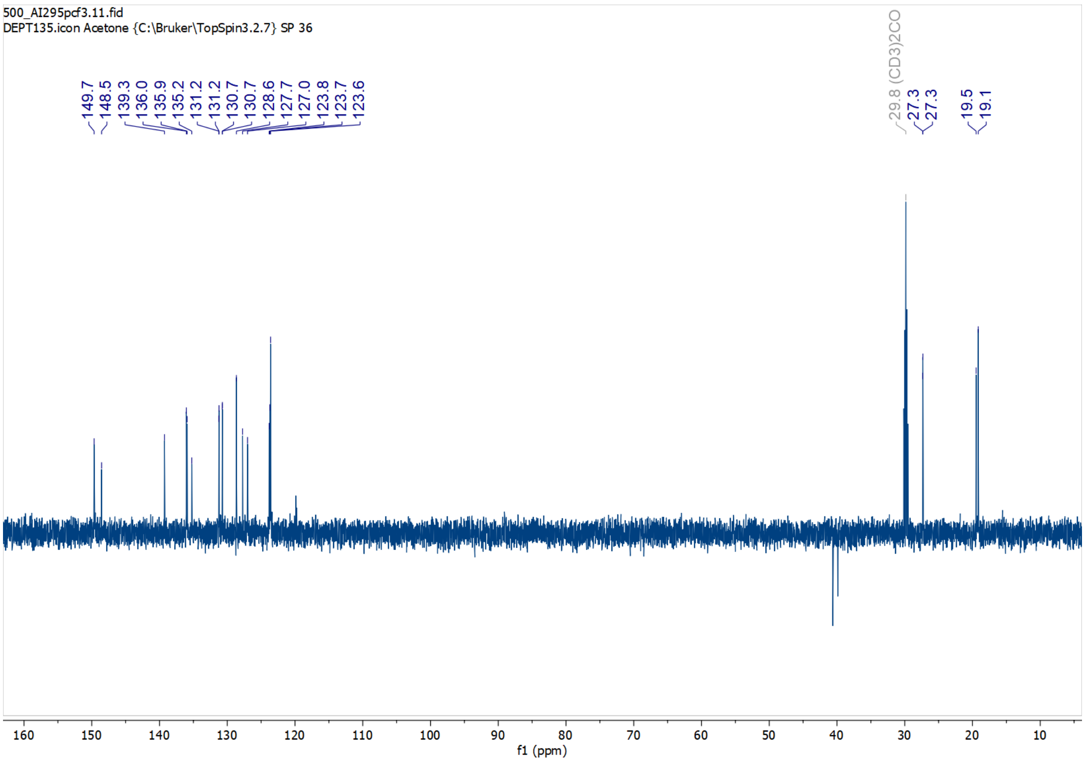

Figure S44. $^{13}C\{^{1}H\}$ NMR spectrum of $[Ir(tmq)_2(\mathbf{L^2})](PF_6)$ acetone-d6.

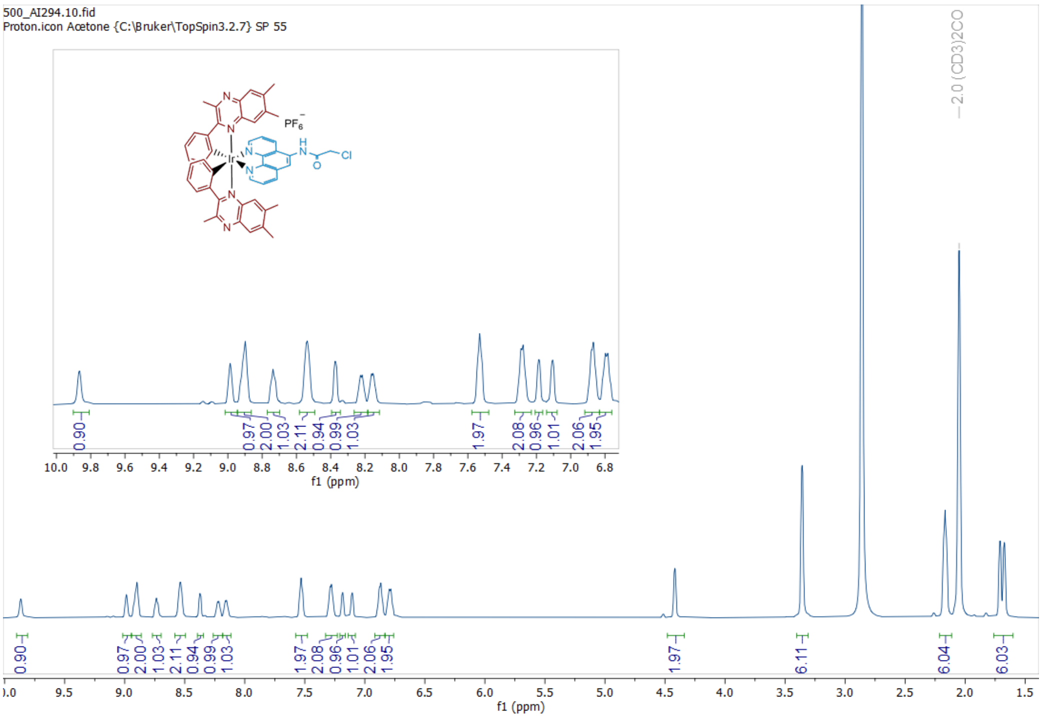

Figure S45. ^{1}H NMR spectrum of $[Ir(tmq)_2($**L**$^3)](PF_6)$ acetone-d6.

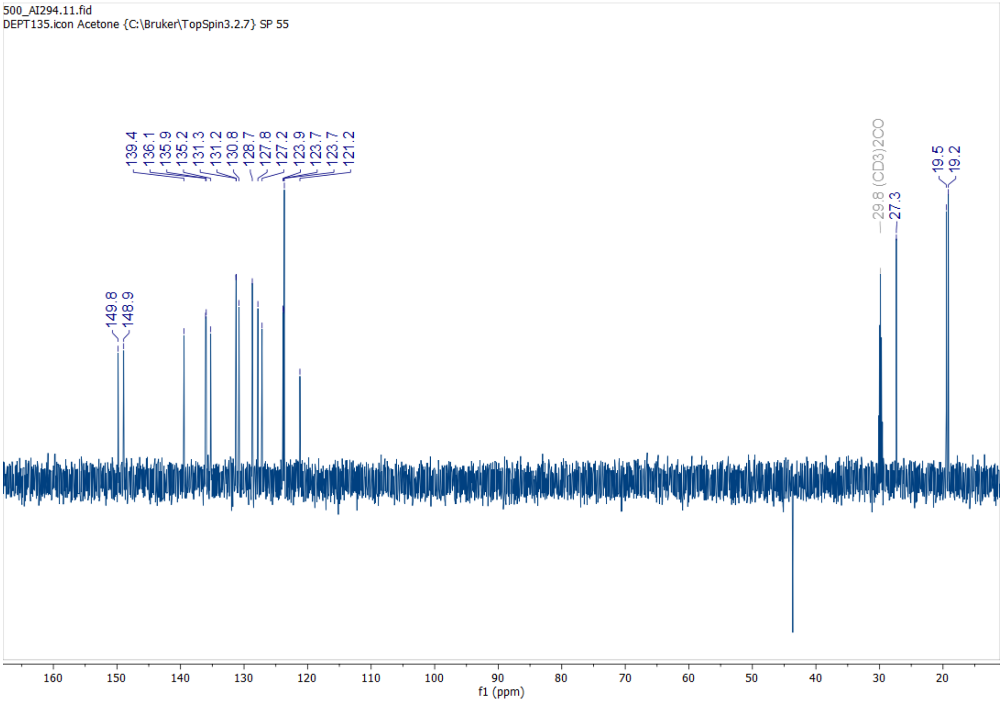

Figure S46. $^{13}C\{^{1}H\}$ NMR spectrum of $[Ir(tmq)_2($**L**$^3)](PF_6)$ acetone-d6.

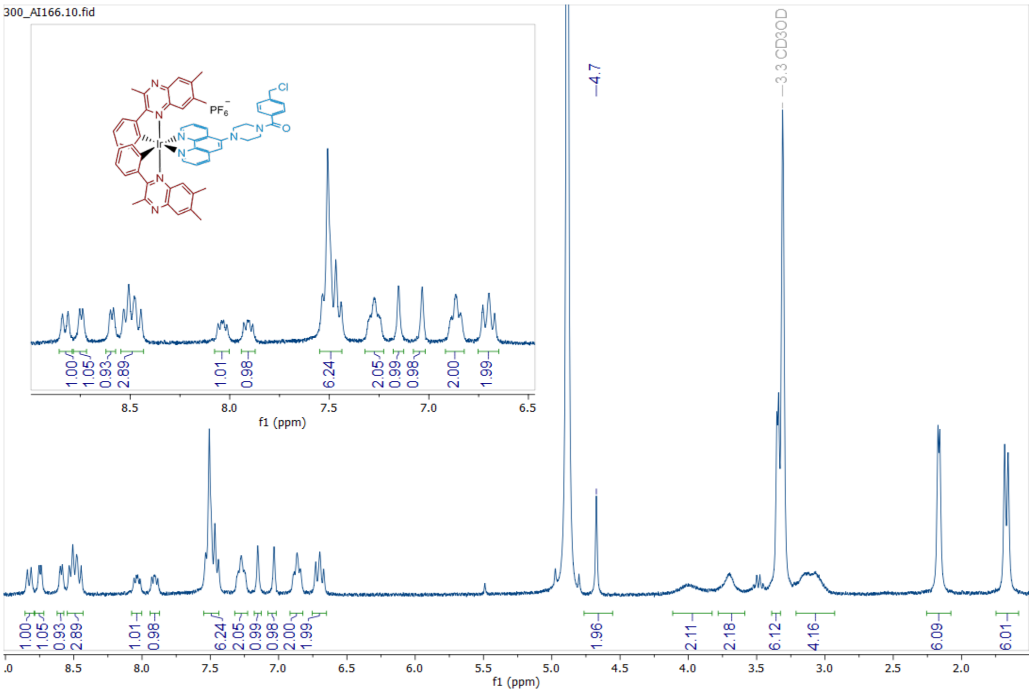

Figure S47. ^{1}H NMR spectrum of [Ir(tmq)$_2$(**L^4**)](PF$_6$) acetone-d6.

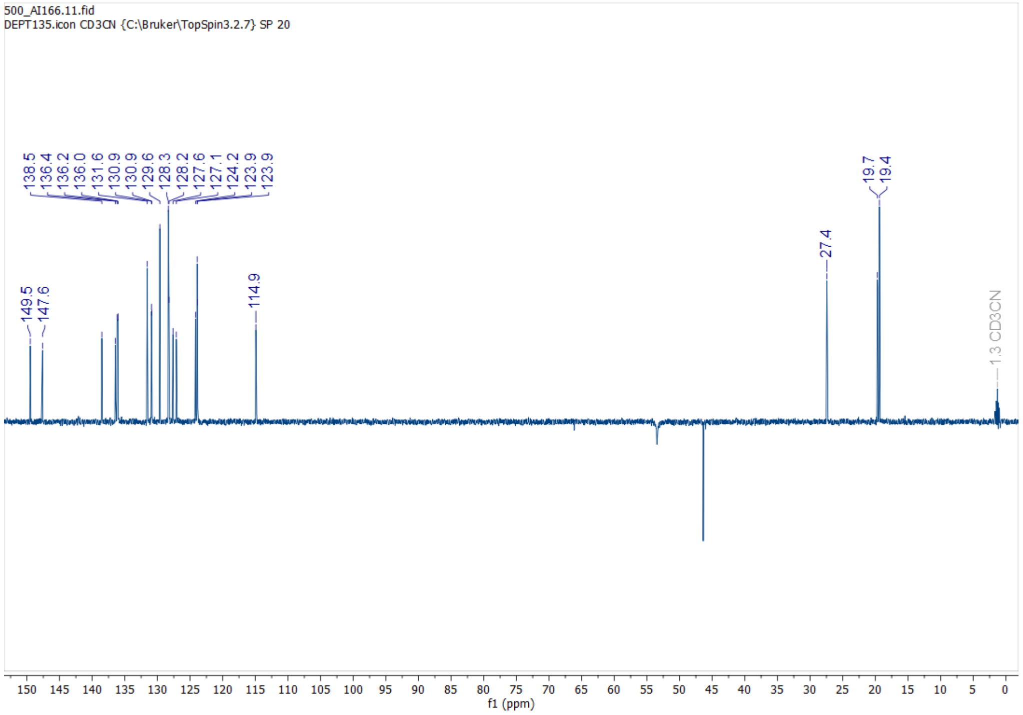

Figure S48. ^{13}C{^{1}H} NMR spectrum of [Ir(tmq)$_2$(**L^4**)](PF$_6$) methanol-d4.

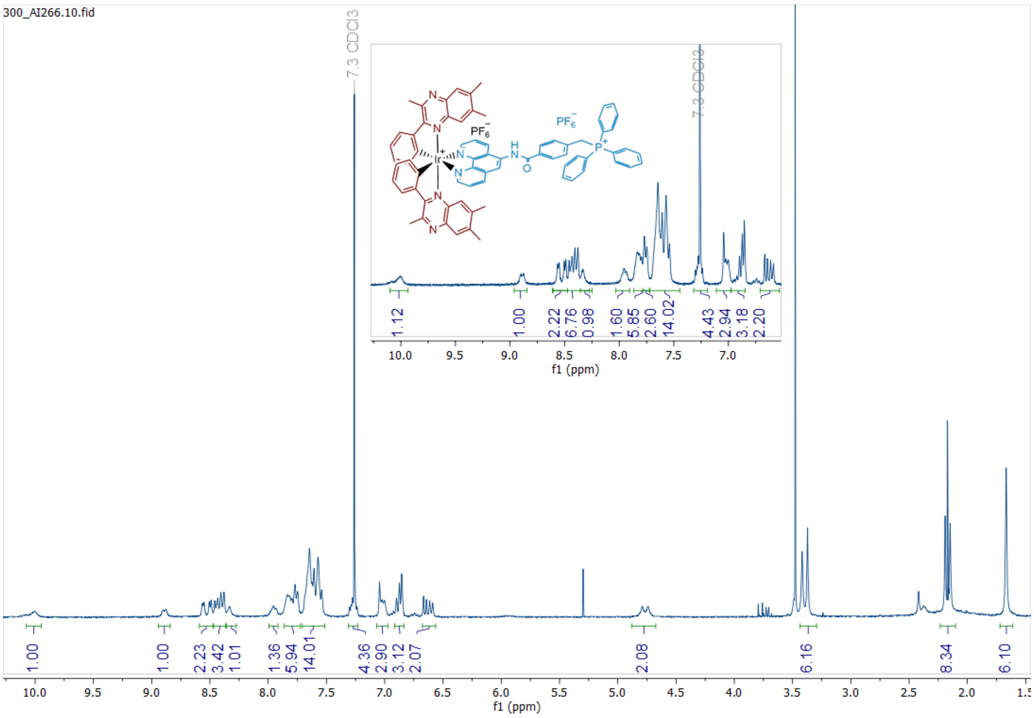

Figure S49. ^{1}H NMR spectrum of $[Ir(tmq)_2(\mathbf{L^5})](PF_6)_2$ acetone-d6.

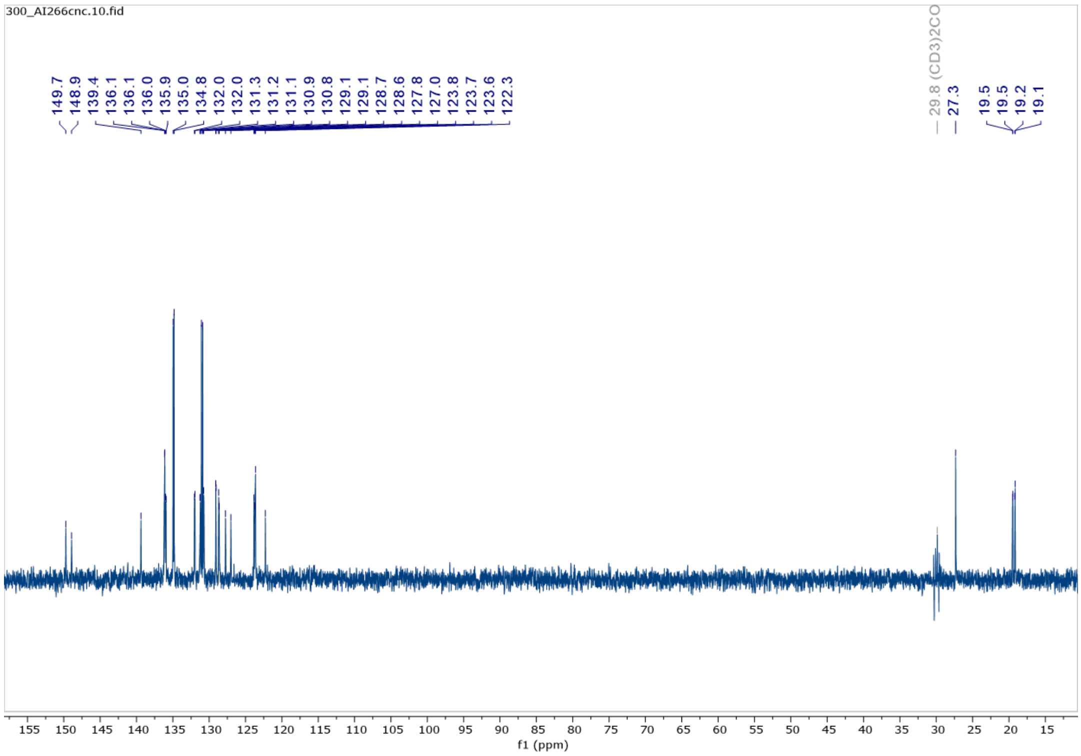

Figure S50. $^{13}C\{^{1}H\}$ NMR spectrum of $[Ir(tmq)_2(\mathbf{L^5})](PF_6)_2$ acetone-d6.

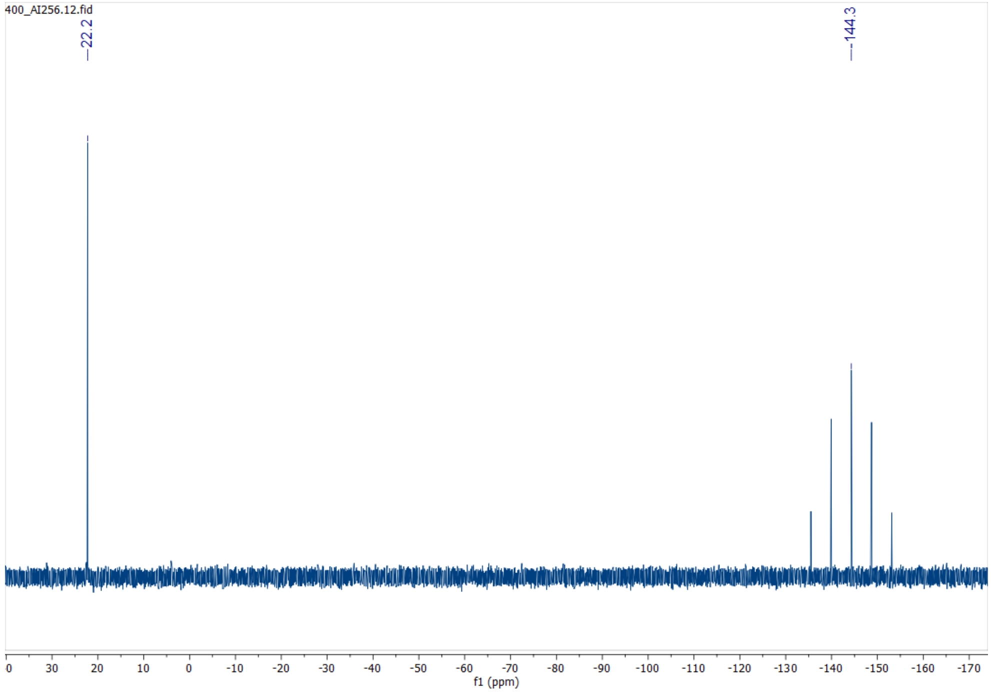

Figure S51. $^{31}P\{^{1}H\}$ NMR spectrum of $[Ir(tmq)_2(\mathbf{L^5})](PF_6)_2$ acetonitrile-d6.

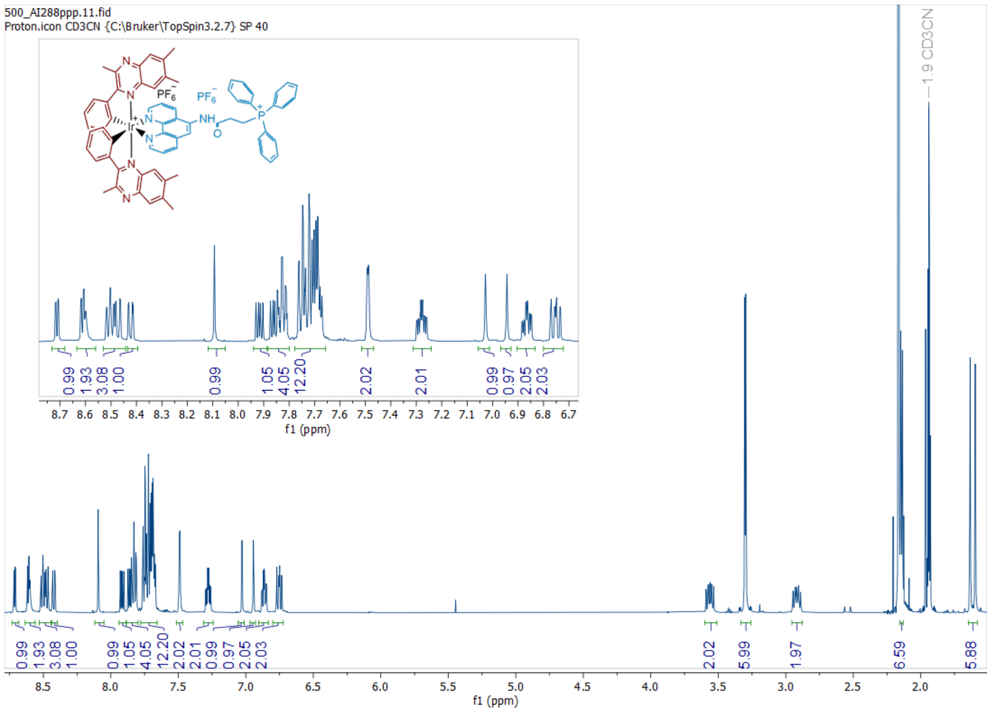

Figure S52. ^{1}H NMR spectrum of $[Ir(tmq)_2(\mathbf{L^6})](PF_6)_2$ acetone-d6.

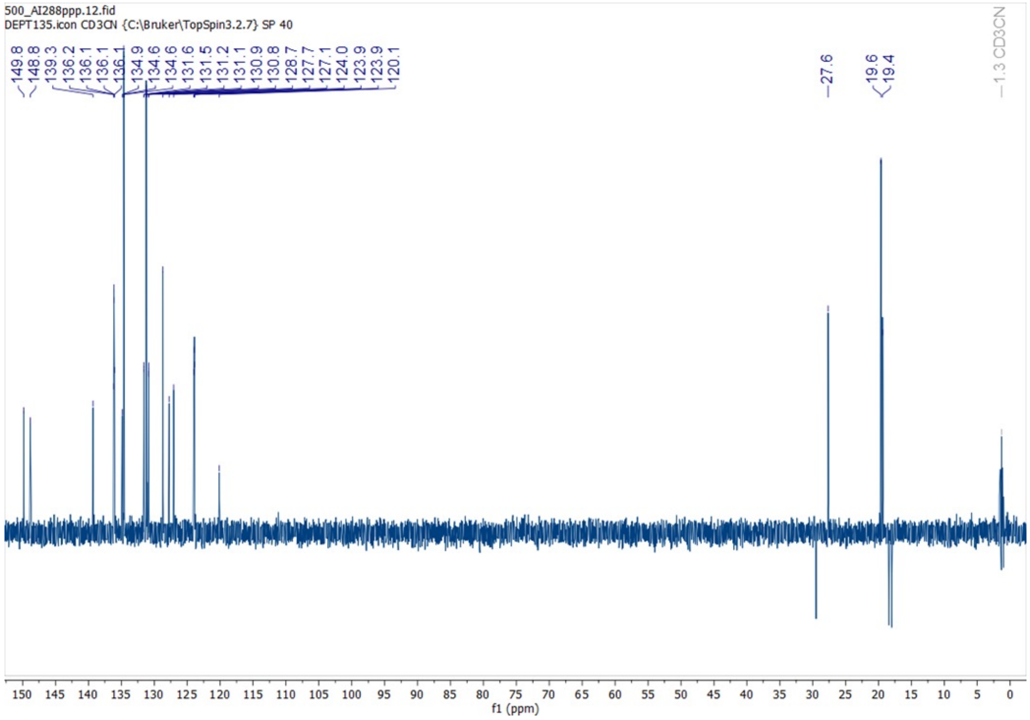

Figure S53. $^{13}C\{^1H\}$ NMR spectrum of $[Ir(tmq)_2(\mathbf{L^6})](PF_6)_2$ acetone-d6.

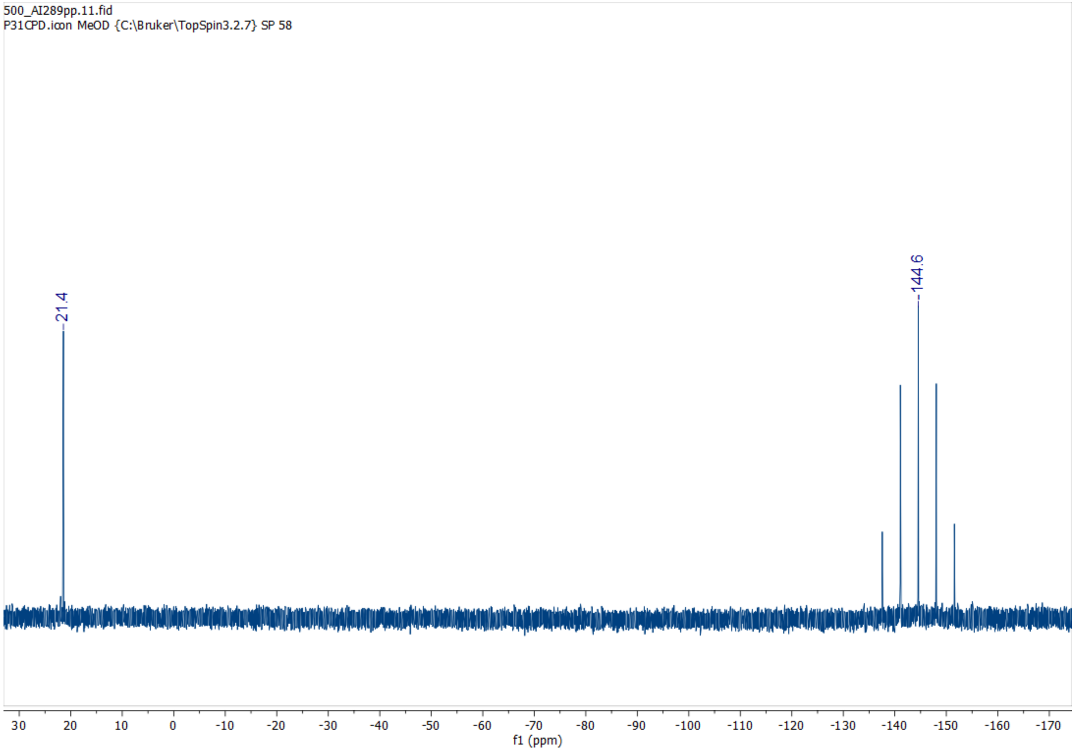

Figure S54. $^{31}P\{^1H\}$ NMR spectrum of $[Ir(tmq)_2(\mathbf{L^6})](PF_6)_2$ acetonitrile-d6.

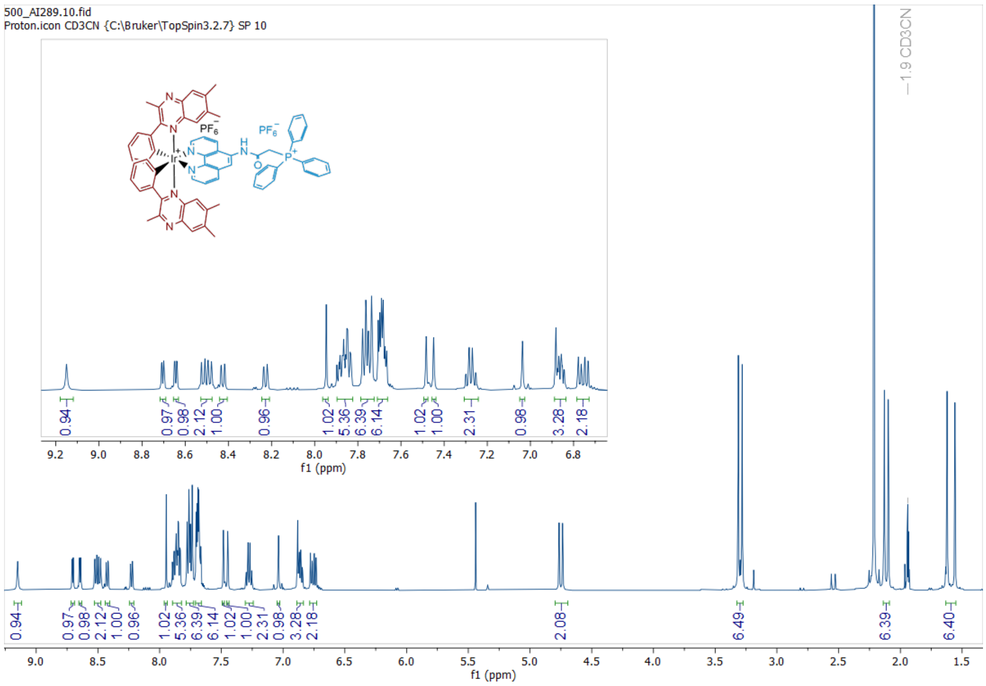

Figure S55. ^{1}H NMR spectrum of $[Ir(tmq)_2(\mathbf{L^7})](PF_6)_2$ acetone-d6.

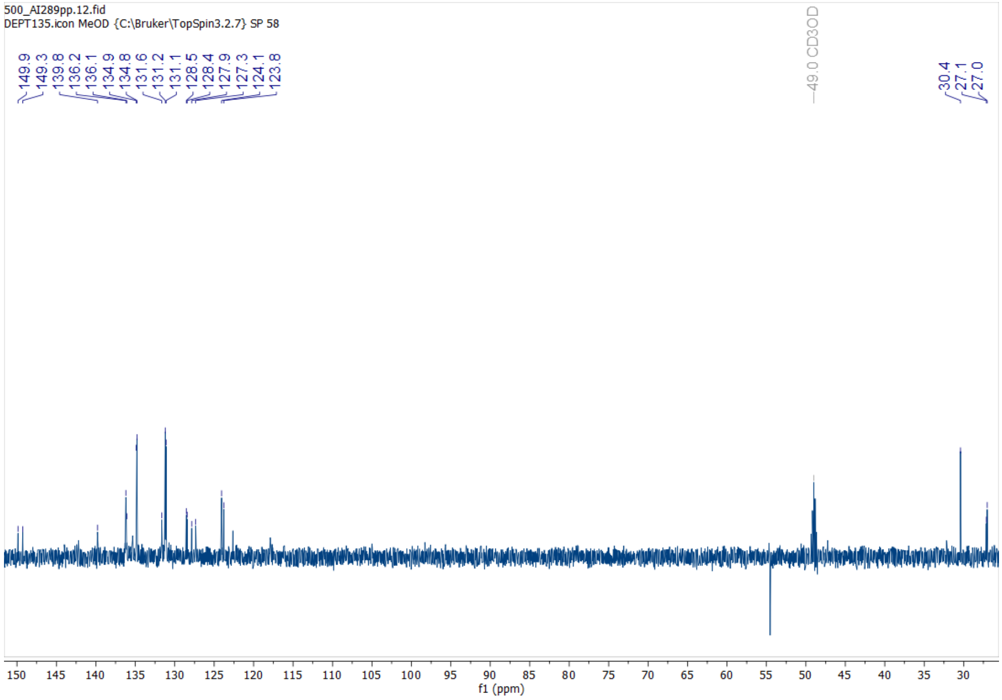

Figure S56. $^{13}C\{^{1}H\}$ NMR spectrum of $[Ir(tmq)_2(\mathbf{L^7})](PF_6)_2$ acetone-d6.

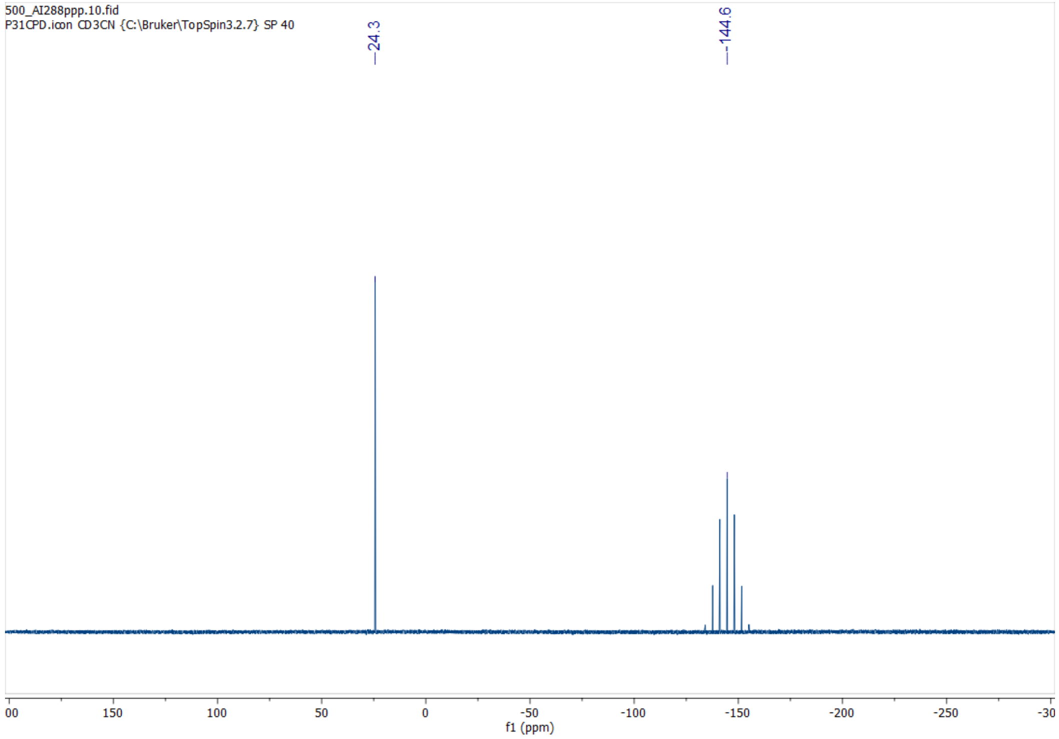

Figure S57. $^{31}P\{^{1}H\}$ NMR spectrum of $[Ir(tmq)_2($**L^7**$)](PF_6)_2$ acetonitrile-d6.

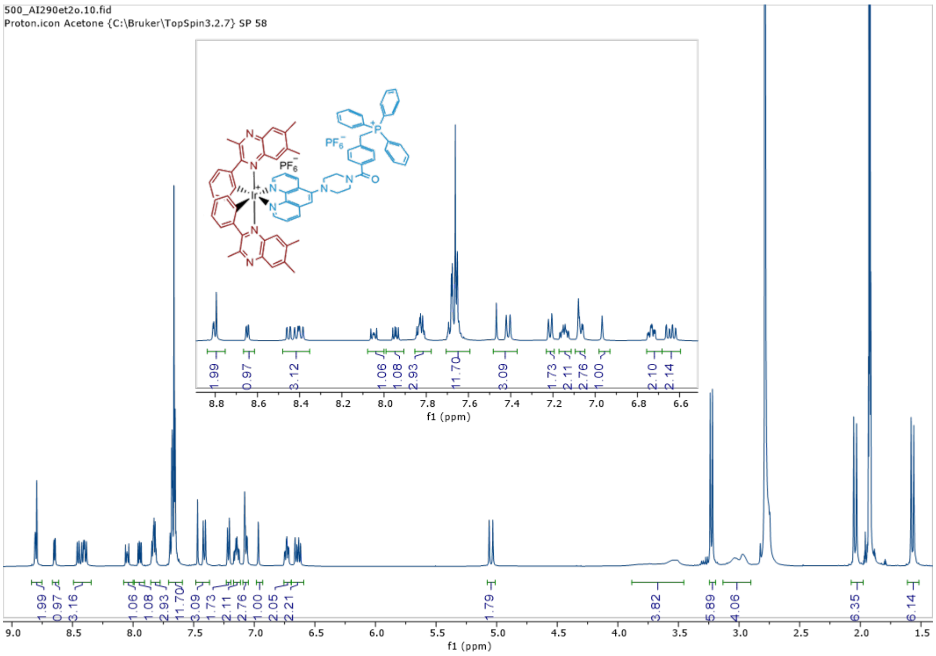

Figure S58. ^{1}H NMR spectrum of $[Ir(tmq)_2($**L^8**$)](PF_6)_2$ acetone-d6.

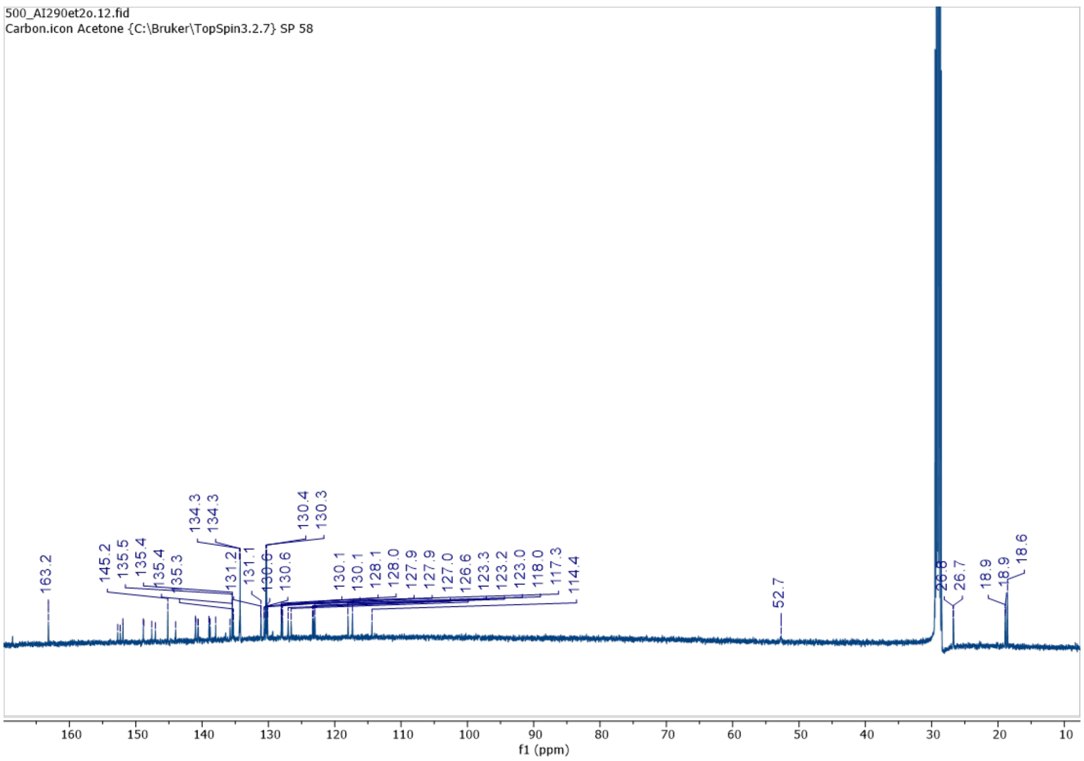

Figure S59. $^{13}C\{^{1}H\}$ NMR spectrum of $[Ir(tmq)_2(\mathbf{L^8})](PF_6)_2$ acetone-d6.

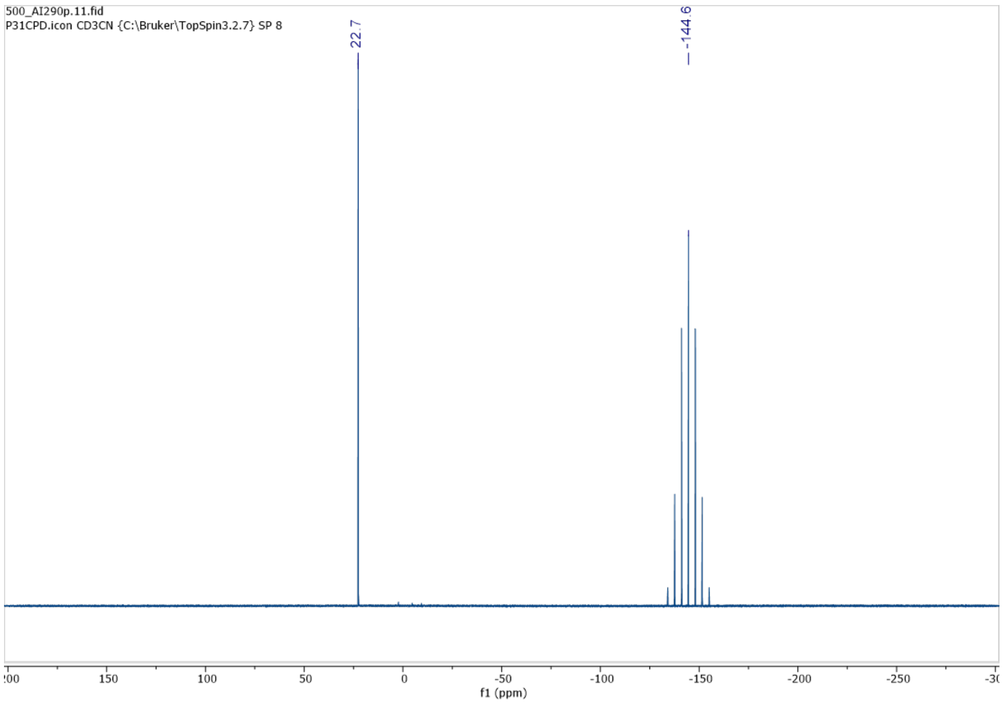

Figure S60. $^{31}P\{^{1}H\}$ NMR spectrum of $[Ir(tmq)_2(\mathbf{L^8})](PF_6)_2$ acetone-d6.

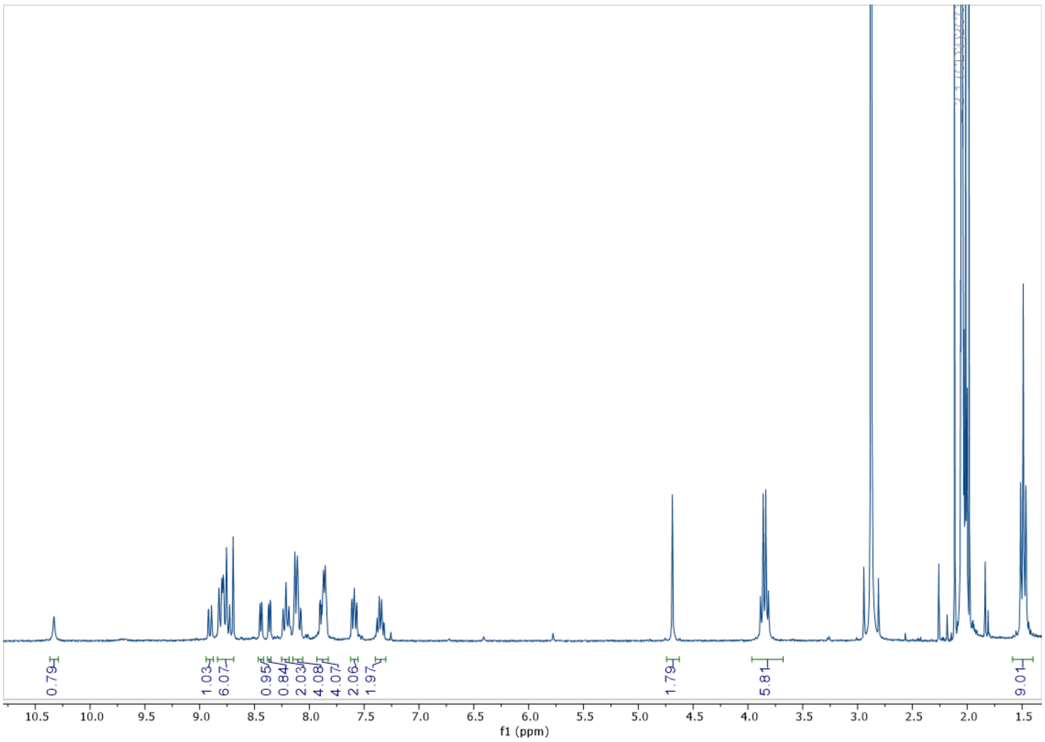

Figure S61. ^{1}H NMR spectrum of [Ru(bipy)$_2$(**L^9**)](PF$_6$)$_3$ acetone-d6.

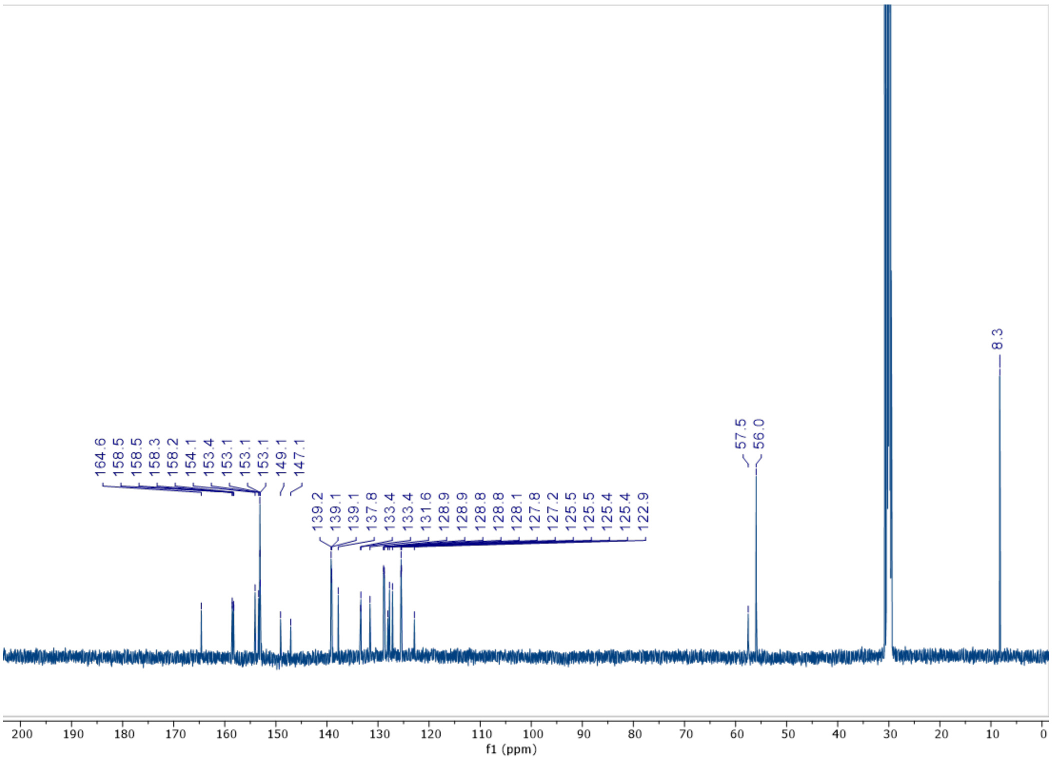

Figure S62. ^{13}C{^{1}H} NMR spectrum of [Ru(bipy)$_2$(**L^9**)](PF$_6$)$_3$ acetone-d6.

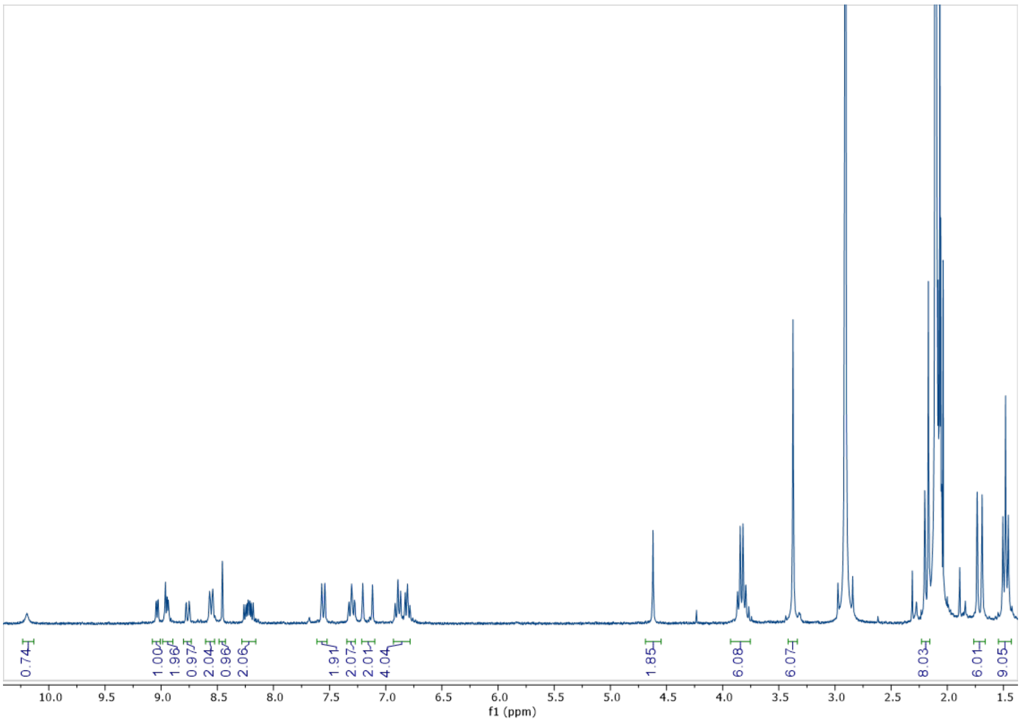

Figure S63. ^{1}H NMR spectrum of $[Ir(tmq)_2(\mathbf{L^9})](PF_6)_2$ acetone-d6.

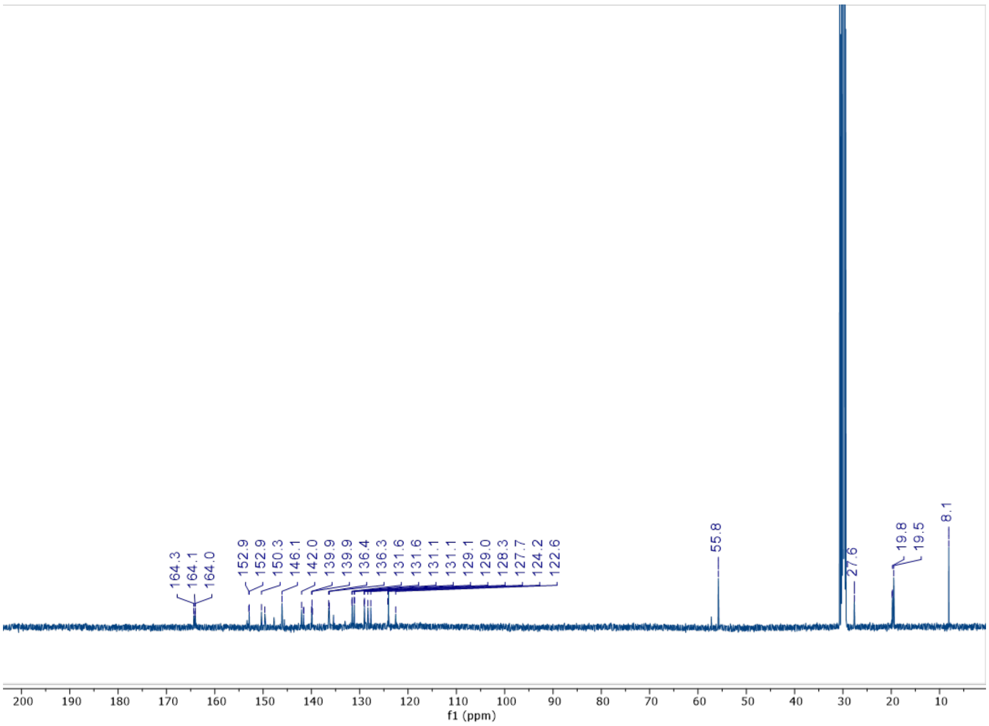

Figure S64. $^{13}C\{^{1}H\}$ NMR spectrum of $[Ir(tmq)_2(\mathbf{L^9})](PF_6)_2$ acetone-d6.

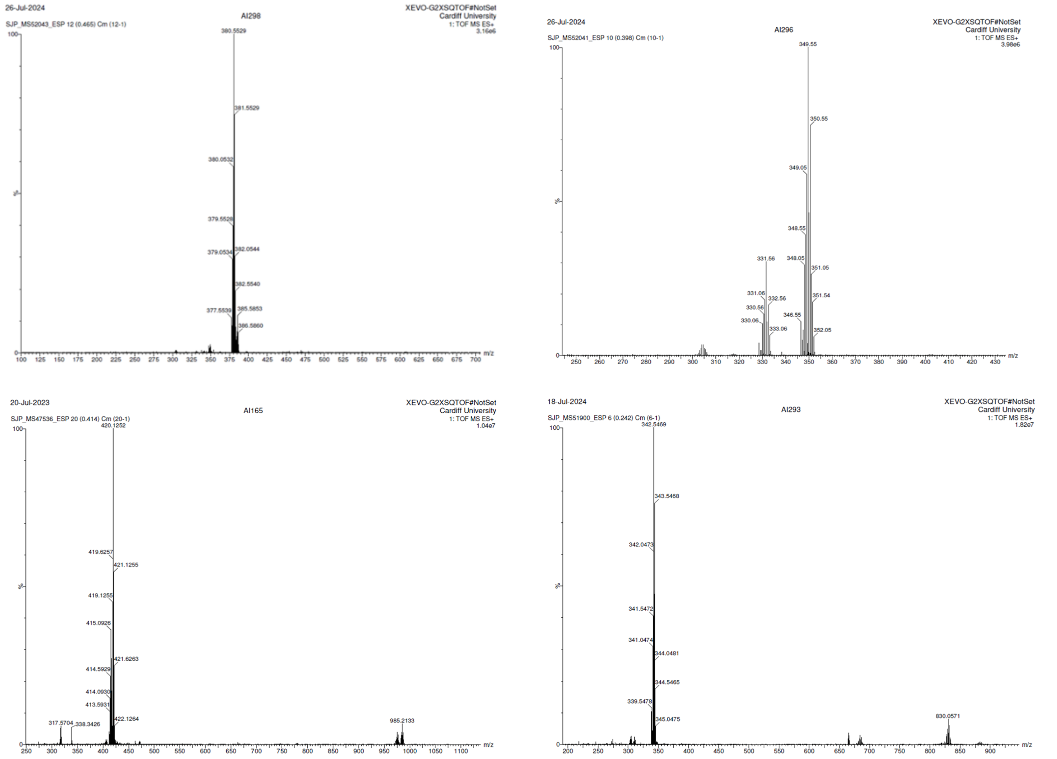

Figure S65. HR MS data for the for (clockwise from top left) [Ru(bipy)$_2$(**L$^{1-4}$**)](PF$_6$)$_2$.

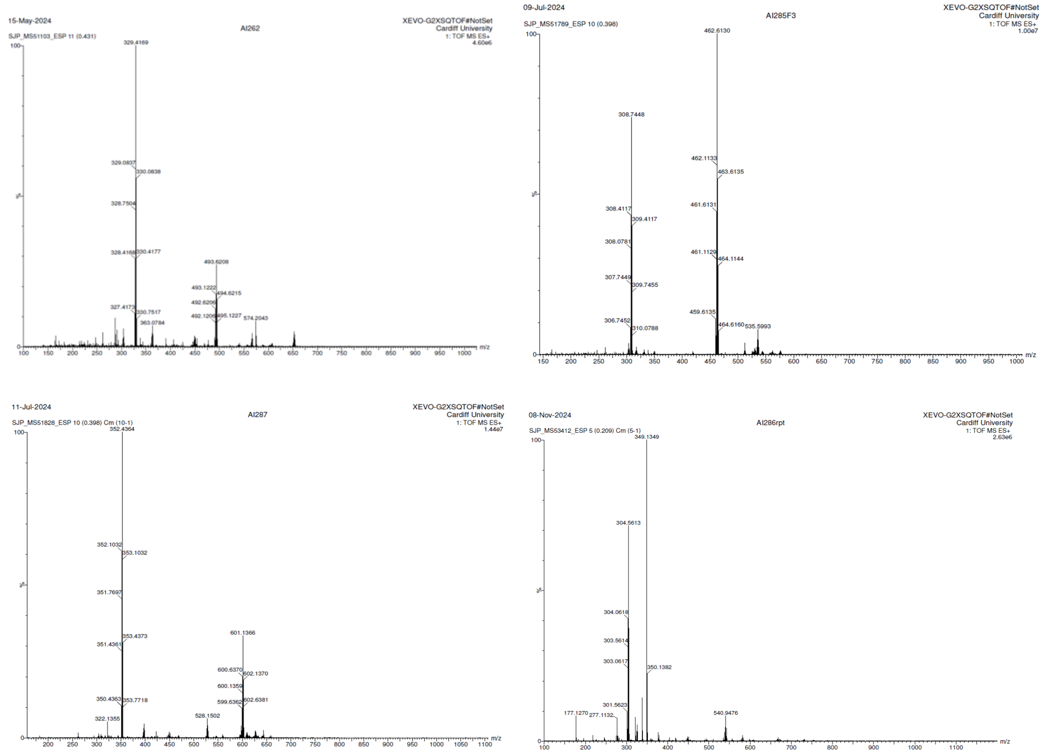

Figure S66. HR MS data for the for (clockwise from top left) [Ru(bipy)$_2$(**L$^{5-8}$**)](PF$_6$)$_3$.

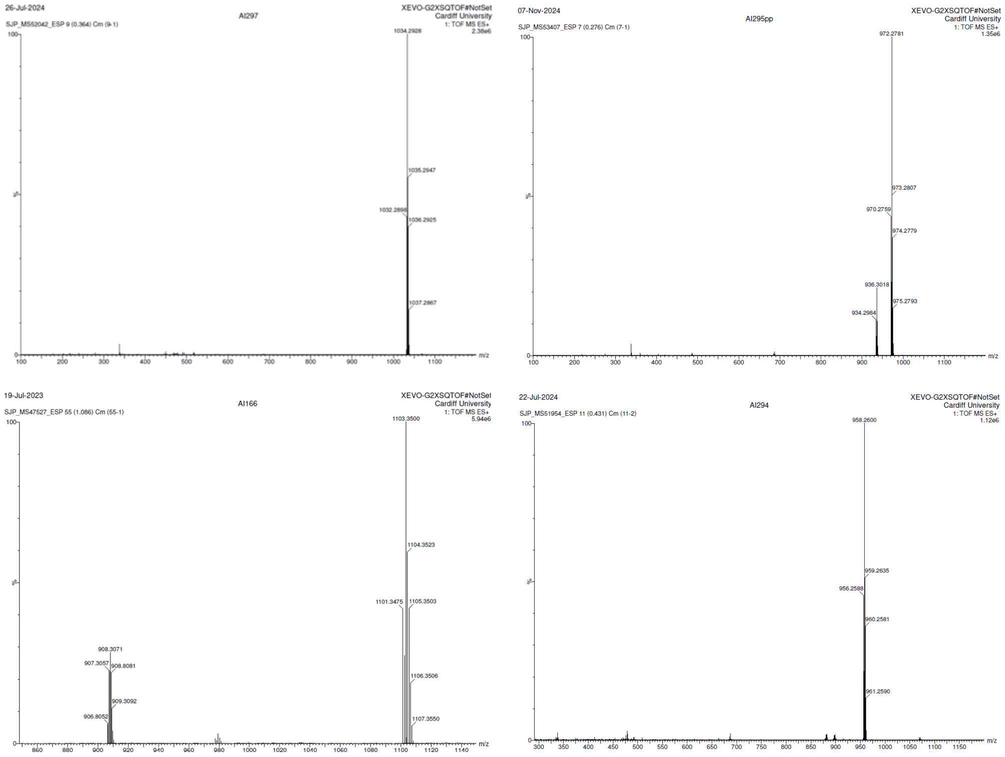

Figure S67. HR MS data for the for (clockwise from top left) [Ir(tmq)$_2$(**L$^{1-4}$**)](PF$_6$)

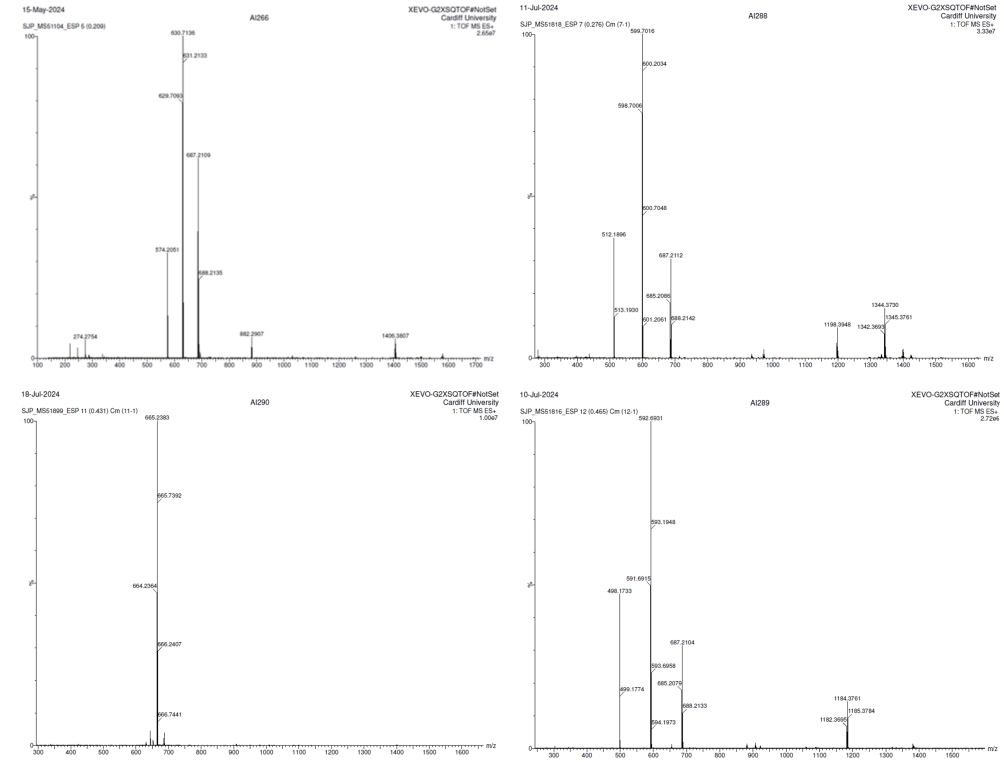

Figure S68. HR MS data for the for (clockwise from top left) [Ir(tmq)$_2$(**L$^{5-8}$**)](PF$_6$)$_2$

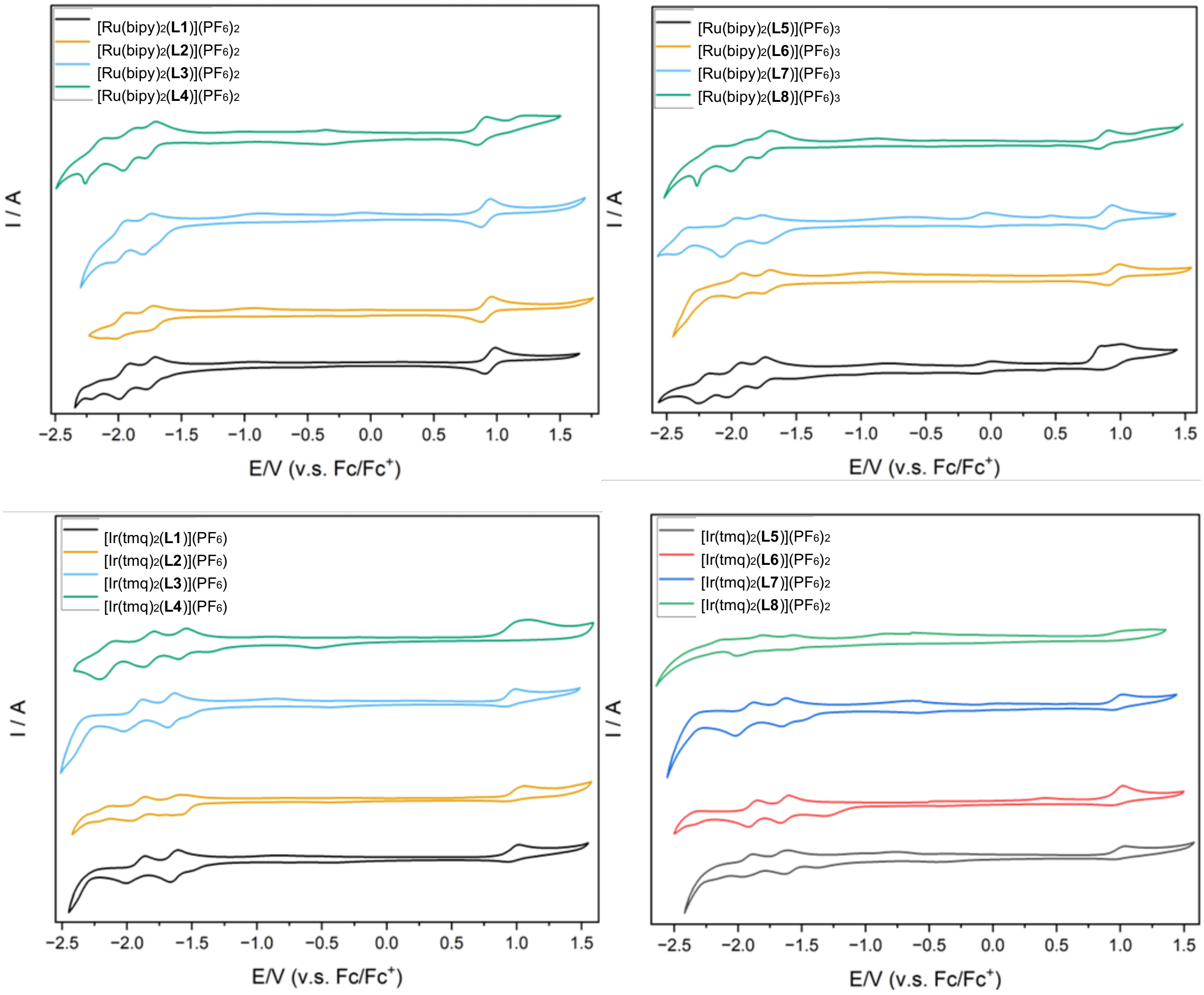

Figure S69. The cyclic voltammograms of $[Ru(bipy)_2(\mathbf{L})](PF_6)_n$ (top) and $[Ir(tmq)_2(\mathbf{L})](PF_6)_n$ (bottom) referenced against Fc/Fc^+ couple at scan rate 250 mV/s (1 mM complex in N_2 purged MeCN with 0.1 M $[^nBu_4N][PF_6]$).

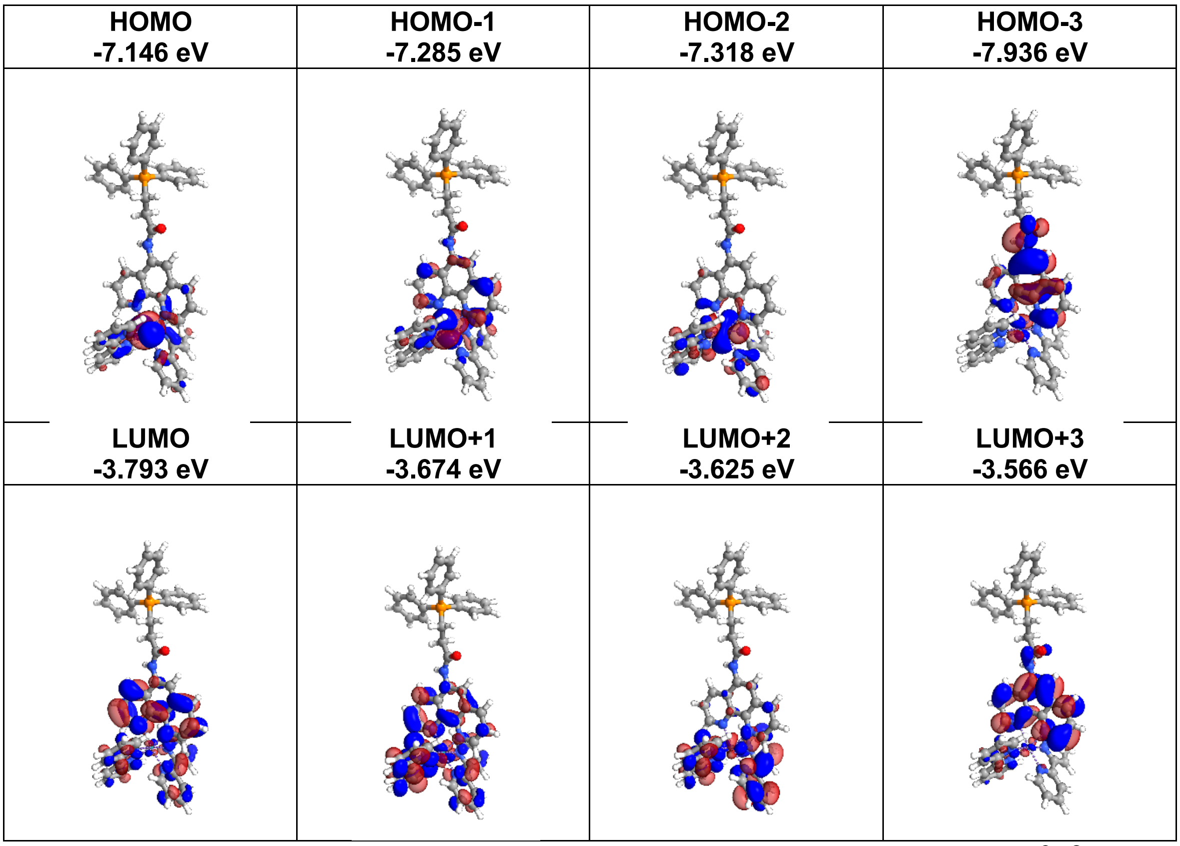

Figure S70. Calculated Kohn-Sham molecular orbitals for [Ru(bipy)$_2$(**L^6**)]$^{2+}$.

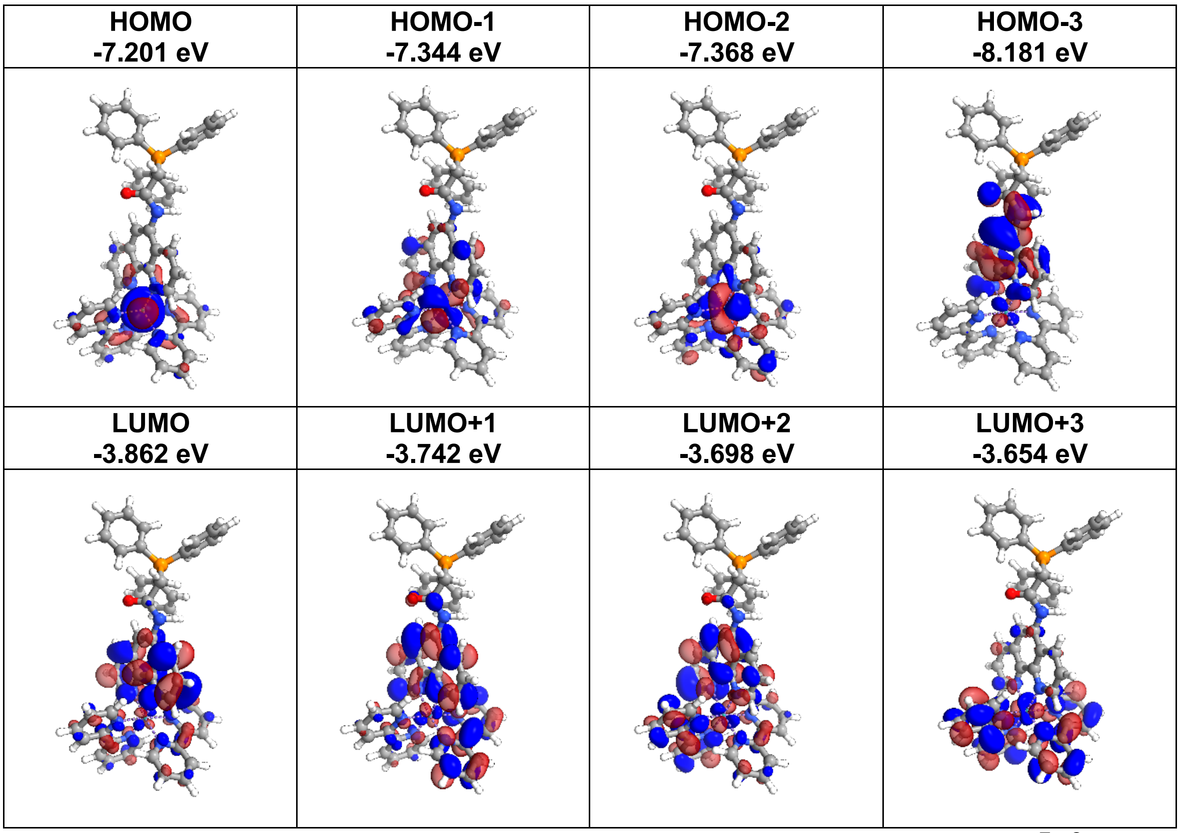

Figure S71. Calculated Kohn-Sham molecular orbitals for [Ru(bipy)$_2$(**L^7**)]$^{2+}$.

HOMO<br>-6.963 eV	HOMO-1<br>-7.039 eV	HOMO-2<br>-7.218 eV	HOMO-3<br>-7.228 eV
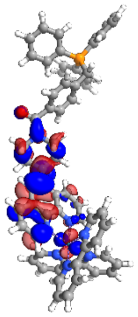	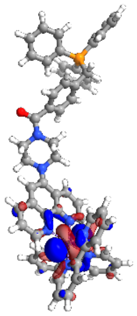	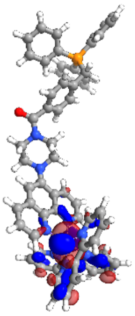	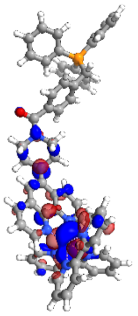
**LUMO<br>-3.674 eV**	**LUMO+1<br>-3.563 eV**	**LUMO+2<br>-3.522 eV**	**LUMO+3<br>-3.310 eV**
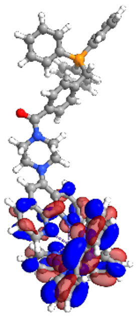	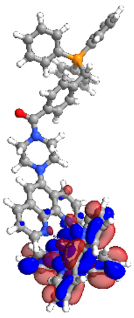	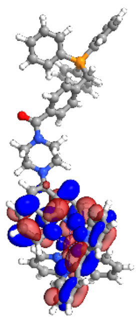	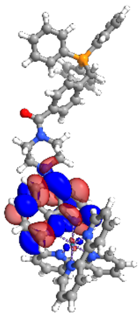

Figure S72. Calculated Kohn-Sham molecular orbitals for $[Ru(bipy)_2(\mathbf{L^8})]^{2+}$.

HOMO<br>-6.515 eV	HOMO-1<br>-6.830 eV	HOMO-2<br>-7.096 eV	HOMO-3<br>-7.188 eV
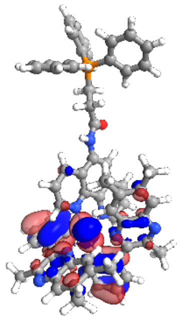	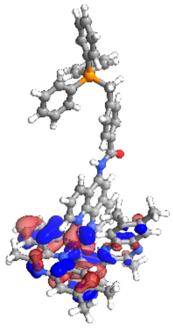	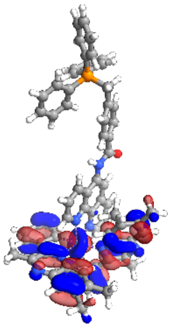	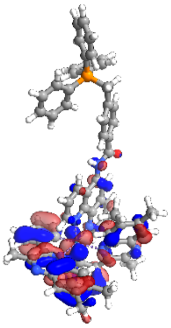
**LUMO<br>-3.386 eV**	**LUMO+1<br>-3.298 eV**	**LUMO+2<br>-3.138 eV**	**LUMO+3<br>-3.013 eV**
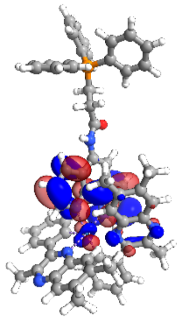	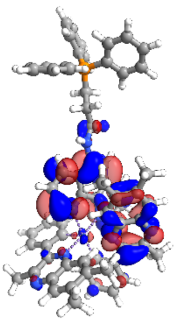	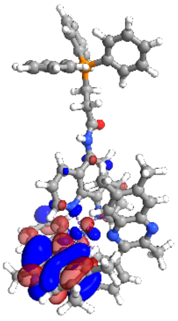	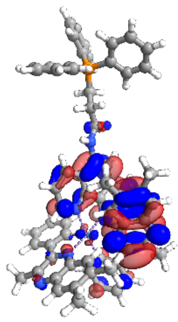

Figure S73. Calculated Kohn-Sham molecular orbitals for $[Ir(tmq)_2(\mathbf{L^6})]^{2+}$.

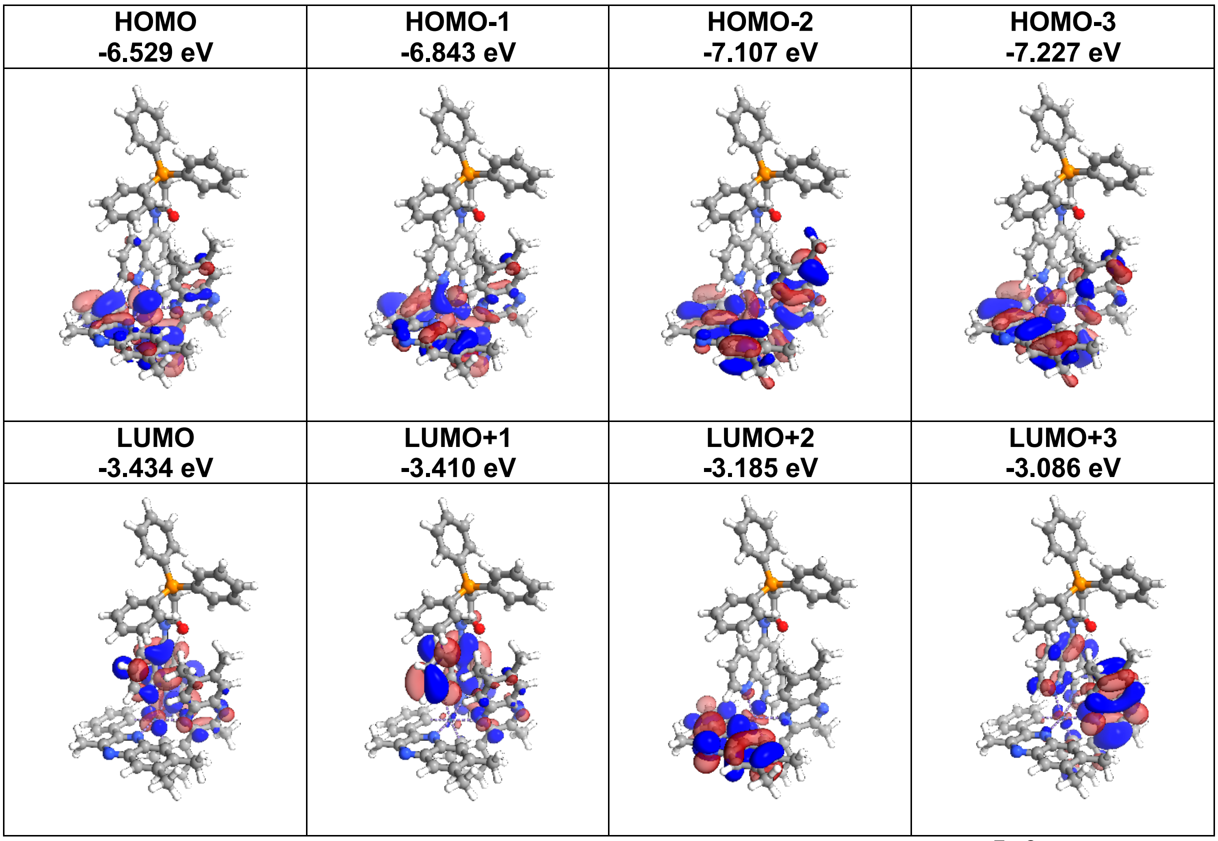

Figure S74. Calculated Kohn-Sham molecular orbitals for $[Ir(tmq)_2(\mathbf{L^7})]^{2+}$.

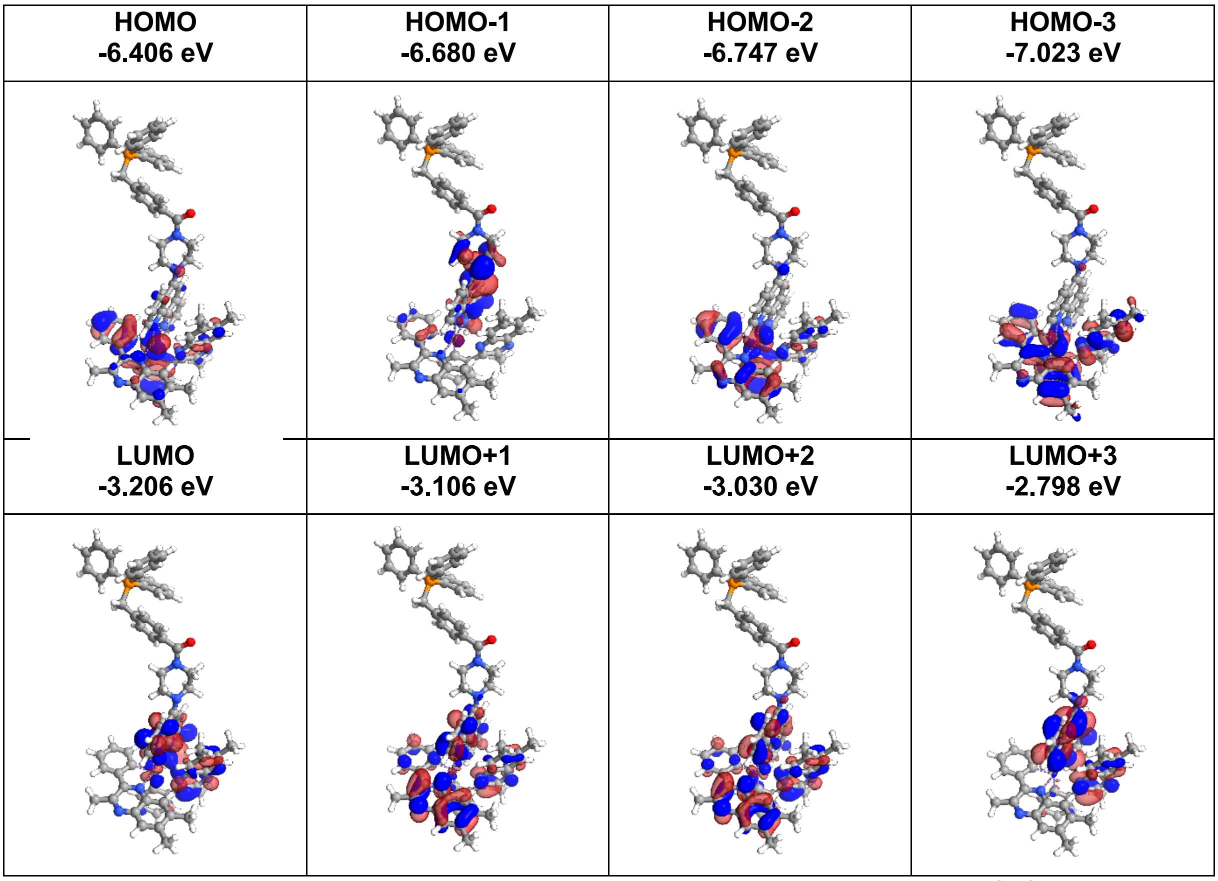

Figure S75. Calculated Kohn-Sham molecular orbitals for $[Ir(tmq)_2(\mathbf{L^8})]^{2+}$.

Table S1. Data collection parameters for the X-ray crystal structures.

Sample	L^5 *(ICl)*	L^5 *(I_3)*	$[Ru(bipy)_2(L^5)](PF_6)_3$	$[Ir(tmq)_2(L^6)](PF_6)_2$
Formula	$C_{38}H_{32}Cl_{0.83}I_{1.17}N_3O_2P$	$C_{39}H_{29}D_4I_3N_3O_2P$	$C_{59.5}H_{51}F_{18}N_7O_{2.5}P_4Ru$	$C_{67}H_{57}F_{12}IrN_7OP_3$
$D_{calc.}$/ g cm^{-3}	1.554	1.800	1.649	1.599
μ/mm^{-1}	1.284	2.645	0.482	2.323
Formula Weight	771.30	991.38	1471.02	1489.30
Colour	yellow	yellow	orange	red
Shape	rod-shaped	blade-shaped	blade-shaped	plate-shaped
Size/mm^3	0.060×0.020×0.015	0.190×0.160×0.040	0.130×0.030×0.015	0.140×0.110×0.005
T/K	100(2)	100(2)	100(2)	100(2)
Crystal System	monoclinic	monoclinic	triclinic	triclinic
Space Group	$P2_1/n$	$P2_1/c$	P-1	P-1
a/Å	16.1564(4)	13.63040(10)	9.2069(2)	14.3429(4)
b/Å	12.5841(3)	13.57240(10)	17.8371(5)	20.1024(7)
c/Å	16.7797(3)	19.7764(2)	18.3094(5)	21.9157(5)
α/°	90	90	86.410(2)	98.801(2)
β/°	104.946(2)	90.7880(10)	86.353(2)	96.938(2)
γ/°	90	90	81.412(2)	92.522(2)
V/Å^3	3296.13(13)	3658.23(5)	2962.81(13)	6186.2(3)
Z	4	4	2	4
Z'	1	1	1	2
Wavelength/Å	0.71075	0.71075	0.71075	0.71075
Radiation type	Mo Kα	Mo Kα	Mo Kα	Mo Kα
Θ_{min}/°	2.248	1.820	2.232	1.880
Θ_{max}/°	34.339	34.338	28.700	25.681
Measured Refl's.	107216	156239	76349	129886
Indep't Refl's	13784	15331	15293	23495
Refl's I≥2 σ(I)	10681	14107	10443	12624
R_{int}	0.0545	0.0258	0.0667	0.0949
Parameters	449	440	869	2545
Restraints	368	1	345	12170
Largest Peak	2.051	2.851	1.076	3.476
Deepest Hole	-0.948	-1.028	-0.498	-1.231
GooF	1.055	1.065	1.023	1.519
wR_2 (all data)	0.0987	0.0730	0.1374	0.4400
wR_2	0.0921	0.0715	0.1228	0.4043
R_1 (all data)	0.0627	0.0296	0.0987	0.2097
R_1	0.0412	0.0264	0.0578	0.1406

Comment: For $[Ir(tmq)_2(\mathbf{L^6})]^{2+}$ cations disordered, one over the majority of the cation, the second just from the amide side-arm of the **L⁶** ligand, which is used for the example measurements that follow. This results in some B-Alerts in CheckCIF. Most relate to averaging when applying restraints to the disorder modelling of PF_6 anions, with the rest involving residual densities near the Ir(III) ions. This is expected as there is further minor disorder which cannot be suitably modelled without ruining the given model because there is insufficient information in the diffraction.

For L5(I_3), (this was mentioned within the CIF) it was observed that the crystals were not single and appeared to grow as two different component systems, but it was not possible to suitably integrate and/or deconvolute the minor component. This means that measured intensities can sometimes differ significantly more than would be expected from calculated intensities. It is assumed that a consequence of the slightly poorer fit to the model are larger residual densities which appear as B-alerts in the checkCIF report.

Table S2. Selected redox data of the complexes (vs. Fc/Fc$^+$ couple at scan rate 250 mV/s. Measured in N_2 purged MeCN) identifying the metal-centered oxidation process.[a]

**Complex**	**Ru$^{II/III}$**	
	***E*$_{1/2}$**	**ΔE**
[Ru(bipy)$_2$(**L^1**)][PF$_6$]$_2$	0.909	71
[Ru(bipy)$_2$(**L^2**)][PF$_6$]$_2$	0.894	80
[Ru(bipy)$_2$(**L^3**)][PF$_6$]$_2$	0.900	60
[Ru(bipy)$_2$(**L^4**)][PF$_6$]$_2$	0.889	75
[Ru(bipy)$_2$(**L^5**)][PF$_6$]$_3$	0.932	76
[Ru(bipy)$_2$(**L^6**)][PF$_6$]$_3$	0.904	81
[Ru(bipy)$_2$(**L^7**)][PF$_6$]$_3$	0.901	137
[Ru(bipy)$_2$(**L^8**)][PF$_6$]$_3$	0.894	66
	**Ir$^{III/IV}$**	
[Ir(tmq)$_2$(**L^1**)]PF$_6$	0.987	34
[Ir(tmq)$_2$(**L^2**)]PF$_6$	1.003	154
[Ir(tmq)$_2$(**L^3**)]PF$_6$	0.993	70
[Ir(tmq)$_2$(**L^4**)]PF$_6$	1.010	N/A
[Ir(tmq)$_2$(**L^5**)][PF$_6$]$_2$	1.071	50
[Ir(tmq)$_2$(**L^6**)][PF$_6$]$_2$	0.997	76
[Ir(tmq)$_2$(**L^7**)][PF$_6$]$_2$	1.014	N/A
[Ir(tmq)$_2$(**L^8**)][PF$_6$]$_2$	1.010	N/A

[a] $E_{1/2}$ is the half-wave potential (V); ΔE is the peak separation (mV)

Table S3. A description of the calculated MO contributions, excited states descriptions and their associated transitions for $[Ru(bipy)_2(\mathbf{L^6})]^{3+}$, where X corresponds to the combined bipy and phenanthroline ligands, and Y from branching nitrogen on the phenanthroline onwards.

	Moiety contribution to Orbital (%)			Orbital contribution to excited state	
**Orbital**	**Ru(4d)**	**X**	**Y**	**Excited state**	**Contributing Transitions (> 10%)**
**LUMO +4**	2%	98%	0%	1 (496 nm f = 0.0010)	HOMO → LUMO (76.6%)
					HOMO → LUMO +1 (19.1%)
**LUMO +3**	2%	93%	5%	2 (483 nm f = 0.0014)	HOMO → LUMO +1 (74.3%)
**LUMO +2**	7%	93%	0%		HOMO → LUMO (21.0%)
**LUMO +1**	6%	93%	1%		
**LUMO**	2%	97%	1%		
**HOMO**	73%	27%	0%	3 (479 nm f = 0.0002)	HOMO → LUMO +2 (94.6%)
**HOMO -1**	61%	38%	1%	4 (459 nm f = 0.0074)	HOMO -2 → LUMO (58.9%)
					HOMO -2 → LUMO +1 (21.0%)
**HOMO -2**	66%	34%	0%	5 (451 nm f = 0.0587)	HOMO -1 → LUMO (68.4%)
					HOMO -1 → LUMO +1 (19.2%)
**HOMO -3**	6%	69%	25%	6 (449 nm f = 0.0097)	HOMO -1 → LUMO +2 (41.9%)
					HOMO -2 → LUMO (29.1%)
**HOMO -4**	1%	99%	0%		HOMO -2 → LUMO +1 (14.9%)

Table S4. A description of the calculated MO contributions, excited states descriptions and their associated transitions for $[Ru(bipy)_2(\mathbf{L^7})]^{3+}$, where X corresponds to the combined bipy and phenanthroline ligands, and Y from branching nitrogen on the phenanthroline onwards.

	Moiety contribution to Orbital (%)			Orbital contribution to excited state	
**Orbital**	**Ru(4d)**	**L1**	**L2**	**Excited state**	**Contributing Transitions (> 10%)**
**LUMO +4**	1%	97%	1%	1 (500 nm f = 0.0009)	HOMO $\rightarrow$ LUMO (82.5%)
					HOMO $\rightarrow$ LUMO +2 (13.9%)
**LUMO +3**	6%	94%	0%	2 (481 nm f = 0.0013)	HOMO $\rightarrow$ LUMO +1 (52.8%)
					HOMO $\rightarrow$ LUMO +2 (22.5%)
**LUMO +2**	5%	93%	2%		HOMO $\rightarrow$ LUMO +3 (12.2%)
					HOMO $\rightarrow$ LUMO (11.0%)
**LUMO +1**	3%	93%	4%		
**LUMO**	2%	96%	2%		
**HOMO**	73%	27%	0%	3 (476 nm f = 0.0003)	HOMO $\rightarrow$ LUMO +3 (64.6%)
					HOMO $\rightarrow$ LUMO +2 (29.7%)
**HOMO -1**	62%	38%	0%	4 (463 nm f = 0.0051)	HOMO -2 $\rightarrow$ LUMO (74.5%)
					HOMO -2 $\rightarrow$ LUMO +2 (13.9%)
**HOMO -2**	66%	34%	0%	5 (453 nm f = 0.0469)	HOMO -1 $\rightarrow$ LUMO (52.1%)
					HOMO $\rightarrow$ LUMO +1 (14.8%)
					HOMO $\rightarrow$ LUMO +2 (11.0%)
**HOMO -3**	4%	71%	25%	6 (447 nm f = 0.0042)	HOMO -1 $\rightarrow$ LUMO +3 (36.6%)
					HOMO -1 $\rightarrow$ LUMO +1 (19.4%)
**HOMO -4**	1%	98%	1%		HOMO -2 $\rightarrow$ LUMO (12.9%)
					HOMO -2 $\rightarrow$ LUMO +1 (11.5%)

Table S5. A description of the calculated MO contributions, excited states descriptions and their associated transitions for $[Ru(bipy)_2(\mathbf{L^8})]^{3+}$, where X corresponds to the combined bipy and phenanthroline ligands, and Y from branching nitrogen on the phenanthroline onwards.

	Moiety contribution to Orbital (%)			Orbital contribution to excited state	
**Orbital**	**Ru(4d)**	**L1**	**L2**	**Excited state**	**Contributing Transitions (> 10%)**
**LUMO +4**	2%	98%	0%	1 (491 nm f = 0.0018)	HOMO -1 → LUMO (88.7%)
**LUMO +3**	1%	95%	4%	2 (488 nm f = 0.0005)	HOMO -1 → LUMO +1 (95.5%)
**LUMO +2**	7%	93%	0%		
**LUMO +1**	7%	93%	0%		
**LUMO**	2%	98%	0%		
**HOMO**	6%	52%	42%	3 (478 nm f = 0.0003)	HOMO -1 → LUMO +2 (86.8%)
**HOMO -1**	73%	27%	0%	4 (461 nm f = 0.0674)	HOMO → LUMO (51.9%)
					HOMO -3 → LUMO (34.8%)
**HOMO -2**	66%	34%	0%	5 (453 nm f = 0.0469)	HOMO -3 → LUMO +1 (46.9%)
					HOMO → LUMO +1 (21.6%)
					HOMO -2 → LUMO (14.3%)
					HOMO -2 → LUMO +2 (11.5%)
**HOMO -3**	58%	33%	9%	6 (448 nm f = 0.0436)	HOMO → LUMO +2 (35.7%)
					HOMO -2 → LUMO (25.1%)
**HOMO -4**	0%	1%	99%		HOMO -2 → LUMO +1 (14.2%)

Table S6. A description of the calculated MO contributions, excited states descriptions and their associated transitions for $[Ir(tmq)_2(\mathbf{L^6})]^{2+}$, where X corresponds to the combined bipy and phenanthroline ligands, and Y from branching nitrogen on the phenanthroline onwards.

	Moiety contribution to Orbital (%)			Orbital contribution to excited state	
**Orbital**	**Ir (5d)**	**L1**	**L2**	**Excited state**	**Contributing Transitions (> 10%)**
**LUMO +4**	0%	0%	100%	1 (505 nm f = 0.0346)	HOMO → LUMO (94.8%)
**LUMO +3**	2%	96%	2%	2 (476 nm f = 0.0259)	HOMO → LUMO +1 (89.9%)
**LUMO +2**	5%	95%	0%		
**LUMO +1**	1%	94%	5%		
**LUMO**	5%	95%	0%		
**HOMO**	32%	68%	0%	3 (462 nm f = 0.0182)	HOMO → LUMO +2 (87.9%)
**HOMO -1**	21%	79%	0%	4 (446 nm f = 0.0018)	HOMO -1 → LUMO (92.1%)
**HOMO -2**	11%	89%	0%	5 (430 nm f = 0.02300)	HOMO → LUMO +3 (91.6%)
**HOMO -3**	12%	87%	0%	6 (421 nm f = 0.0378)	HOMO -1 → LUMO +1 (93.3%)
**HOMO -4**	34%	63%	3%		

Table S7. A description of the calculated MO contributions, excited states descriptions and their associated transitions for $[Ir(tmq)_2(\mathbf{L^7})]^{2+}$, where X corresponds to the combined bipy and phenanthroline ligands, and Y from branching nitrogen on the phenanthroline onwards.

	Moiety contribution to Orbital (%)			Orbital contribution to excited state	
**Orbital**	**Ir (5d)**	**L1**	**L2**	**Excited state**	**Contributing Transitions (> 10%)**
**LUMO +4**	0%	2%	98%	1 (510 nm f = 0.0319)	HOMO → LUMO (94.5%)
**LUMO +3**	2%	96%	2%	2 (491 nm f = 0.0120)	HOMO → LUMO +1 (95,4%)
**LUMO +2**	5%	95%	0%		
**LUMO +1**	1%	96%	3%		
**LUMO**	5%	94%	1%		
**HOMO**	31%	69%	0%	3 (467 nm f = 0.0328)	HOMO → LUMO +2 (94.4%)
**HOMO -1**	23%	77%	0%	4 (452 nm f = 0.0032)	HOMO -1 → LUMO (87.0%)

**HOMO -2**	13%	87%	0%	5 (439 nm f = 0.0288)	HOMO → LUMO +3 (92.3%)
**HOMO -3**	12%	88%	0%	6 (435 nm f = 0.0304)	HOMO -1 → LUMO +1 (87.5%)
**HOMO -4**	25%	75%	0%		

Table S8. A description of the calculated MO contributions, excited states descriptions and their associated transitions for $[Ir(tmq)_2(\mathbf{L^8})]^{2+}$, where X corresponds to the combined bipy and phenanthroline ligands, and Y from branching nitrogen on the phenanthroline onwards.

	**Moiety contribution to Orbital (%)**			**Orbital contribution to excited state**	
**Orbital**	**Ir (5d)**	**L1**	**L2**	**Excited state**	**Contributing Transitions (> 10%)**
**LUMO +4**	0%	0%	100%	1 (493 nm f = 0.0378)	HOMO → LUMO (95.6%)
**LUMO +3**	1%	96%	3%	2 (473 nm f = 0.0407)	HOMO → LUMO +1 (82.6%)<br>HOMO → LUMO +2 (13.0%)
**LUMO +2**	4%	95%	1%		
**LUMO +1**	2%	97%	1%		
**LUMO**	5%	95%	0%		
**HOMO**	32%	67%	1%	3 (456 nm f = 0.0063)	HOMO → LUMO +2 (82.0%)<br>HOMO → LUMO +1 (13.5%)
**HOMO -1**	2%	59%	39%	4 (432 nm f = 0.0057)	HOMO -2 → LUMO (92.8%)
**HOMO -2**	23%	75%	2%	5 (430 nm f = 0.0426)	HOMO -1 → LUMO (79.7%)
**HOMO -3**	14%	85%	1%	6 (413 nm f = 0.0538)	HOMO → LUMO +3 (73.5%)<br>HOMO -2 → LUMO +1 (17.3%)
**HOMO -4**	11%	89%	0%		

Table S9. Superimposed DFT optimised S_0 (blue) and TD-DFT optimised S_1 (beige) geometries for all TPP^+ complexes, along with the root mean square deviation (RMSD) between the two structures.

$[Ru(bipy)_2(\mathbf{L^5})]^{3+}$ RMSD: 0.181 Å	$[Ru(bipy)_2(\mathbf{L^6})]^{3+}$ RMSD: 0.152 Å	$[Ru(bipy)_2(\mathbf{L^7})]^{3+}$ RMSD: 0.119 Å	$[Ru(bipy)_2(\mathbf{L^8})]^{3+}$ RMSD: 0.130 Å
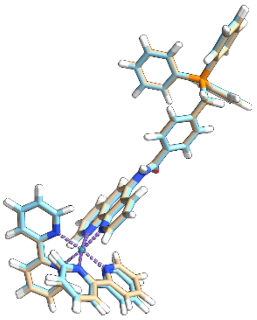	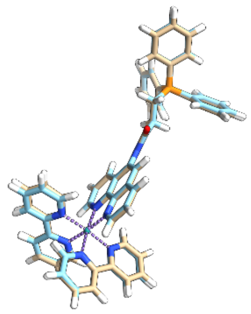	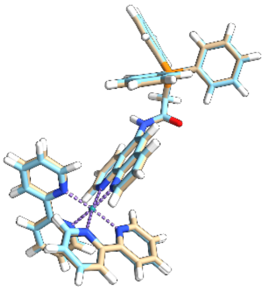	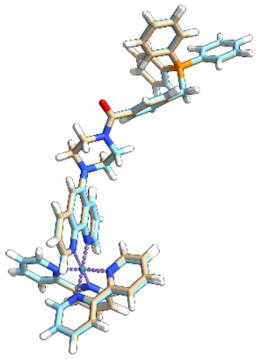

$[Ir(tmq)_2(\mathbf{L^5})]^{2+}$ RMSD: 0.898 Å	$[Ir(tmq)_2(\mathbf{L^6})]^{2+}$ RMSD: 0.557 Å	$[Ir(tmq)_2(\mathbf{L^7})]^{2+}$ RMSD: 0.163 Å	$[Ir(tmq)_2(\mathbf{L^8})]^{2+}$ RMSD: 0.931 Å
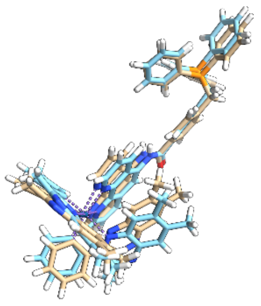	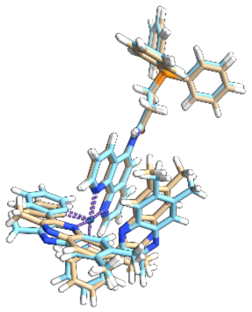	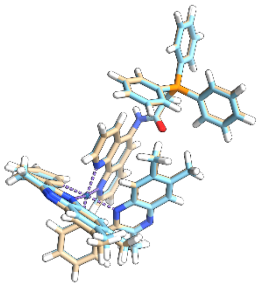	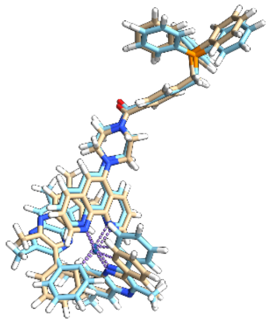
